# Mapping proton and carbon dioxide electrocatalytic reductions at a Rh complex by *in situ* spectroelectrochemical NMR

**DOI:** 10.1039/d5sc05744b

**Published:** 2025-11-10

**Authors:** A.-C. Kick, M. Schatz, C. Kahl, M. Hölscher, R.-A. Eichel, J. Granwehr, N. Kaeffer, W. Leitner

**Affiliations:** a Institute of Technical and Macromolecular Chemistry, RWTH Aachen University Worringerweg 2 52074 Aachen Germany; b Max-Planck-Institute for Chemical Energy Conversion Stiftstraße 34-36 45470 Mülheim an der Ruhr Germany walter.leitner@cec.mpg.de; c Institute of Energy Technologies, Fundamental Electrochemistry (IET-1), Forschungszentrum Jülich Wilhelm-Johnen-Straße 52428 Jülich Germany j.granwehr@fz-juelich.de; d Institute of Physical Chemistry, RWTH Aachen University Landoltweg 2 52074 Aachen Germany; e Faculty of Mechanical Engineering, RWTH Aachen University Eilfschornstraße 18 52062 Aachen Germany; f Université de Strasbourg, Université de Haute-Alsace, CNRS, LIMA, UMR 7042 67000 Strasbourg France nkaeffer@unistra.fr

## Abstract

Detailed molecular level understanding of organometallic electrocatalytic systems is required to fully exploit their technological potential to store, distribute, and utilise renewable energy in chemical form. However, *in situ* methods providing high resolution information on the structure and reactivity of transient intermediates remain challenging due to incompatible requirements for standard electrochemical and spectroscopic cell designs. Here, we demonstrate the use of spectroelectrochemical nuclear magnetic resonance (SEC-NMR) to enable *operando* characterisation of molecular species during organometallic electrocatalysis. The electroreduction of a prototypical molecular rhodium(+i) diphosphine complex was studied under aprotic conditions and in the presence of H_2_O and/or CO_2_. By combining multinuclear SEC-NMR, chemical reductions, modelling and simulations, we determine the involved species, their relative concentrations and the competing interconversions. The bielectronic reduction leading to the highly reactive low-valent rhodium(−i) intermediate and subsequent protonation of that species into a Rh–hydride complex was followed in a time-resolved manner. Deuterium labelling and *ex situ* NMR analysis after SEC-NMR electrolysis revealed that under aprotic conditions the proton source substantially arises from Hofmann elimination of the *n*Bu_4_NPF_6_ electrolyte in addition to the acetonitrile solvent. The reactivities of the Rh(−i) and the Rh–H complexes were further monitored under turnover conditions, providing direct molecular insights into bifurcating electrocatalytic pathways for hydrogen evolution and CO_2_ reduction.

## Abstract 2

Detailed molecular understanding on the electroreduction of a molecular rhodium complex and its electrocatalytic behaviour in H^+^ and CO_2_ reduction is obtained by multinuclear spectroelectrochemical NMR.

## Graphical abstract

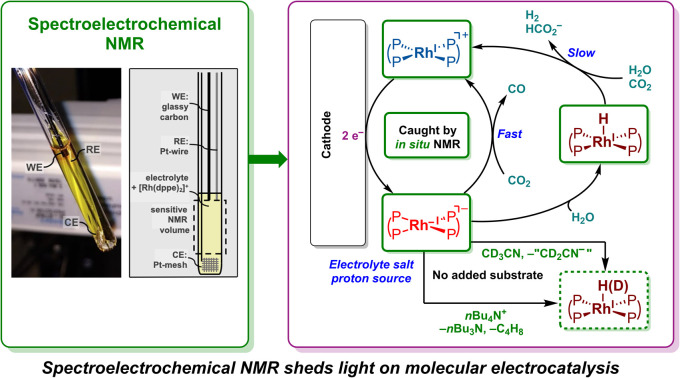

## Introduction

Facing the challenging transition from a linear to a circular economy requires the development of novel catalytic processes at the interface of energy and chemistry.^1^ The defossilisation of the chemical value chain may be advanced by the use of green electricity for the conversion of sustainable carbon sources, among which carbon dioxide (CO_2_) is a major feedstock.^2^ Electrocatalytic reaction systems that directly transform renewable electrical energy into chemical energy carriers and products represent a key technology in this field.^3–8^ Organometallic electrocatalysts provide promising approaches for selective CO_2_ reduction, especially towards C_1_ products such as CO or HCOO^−^.^9,10^ A fundamental parameter to control the bifurcation between these two products relates to CO_2_ reduction occurring either by direct electron transfer from the metal (*ET*_*M*_) or *via* insertion into a metal-hydride bond (*ET*_*H*_).^4^ The formation of metal-hydride species is also highly relevant for the competing hydrogen evolution reaction (HER). To map and eventually manipulate these reaction networks calls for the identification of key intermediates and their interconversion under catalytic conditions. These fundamental questions can be precisely addressed through *in situ* spectroscopic techniques, among which spectroelectrochemical nuclear magnetic resonance (SEC-NMR) has only been used to a limited extent until now despite its considerable potential.^3,11–14^ NMR experiments provide detailed information on the chemical environment of numerous nuclei, molecular compositions, exchange processes, spatial distributions or diffusion, just to mention the most commonly used protocols.^15^

Within the scope of organometallic electrocatalysis, we recently turned our attention to low-valent rhodium complexes. In particular, rhodium–phosphine complexes are known for high chemocatalytic activities and selectivities under thermal reaction conditions,^16–22^ which may transpose to or be complemented by electrochemical transformations. We were particularly drawn to the [Rh(dppe)_2_]^+^ (dppe: diphenylphosphinoethane) complex (noted Rh^I^ in the following) and the reduced congeners for which both *ET*_*H*_ and *ET*_*M*_ pathways have been inferred for chemo- and electrocatalytic CO_2_ reduction.^23,24^ With the availability of different NMR spectroscopic probes, namely ^103^Rh, ^31^P and ^1,2^H nuclei, we reasoned that SEC-NMR offers an ideal method to elucidate the nature of the involved reduced intermediates and their interconversion directly under electrocatalytic conditions.

**Fig. 1:**
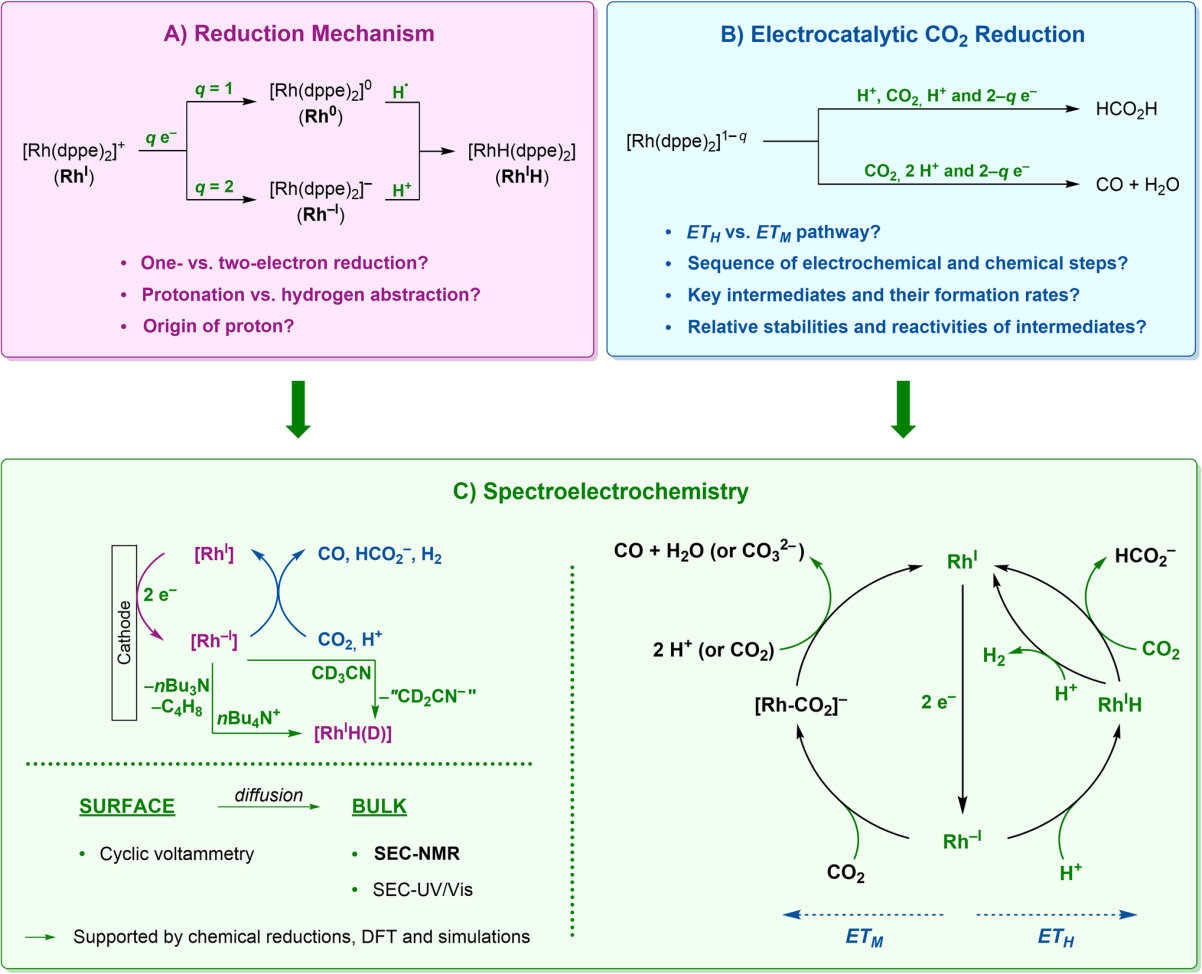
Use of (spectro)electrochemical methods for the elucidation of the reduction mechanism for [Rh(dppe)_2_]^+^ (A) and for the observation of intermediate species in the electrocatalytic reduction of CO_2_ and H^+^ (B). Intermediates, reactions and products marked in green are observed with spectroelectrochemical methods within this work (C).

The reduction of Rh^I^ to the corresponding [Rh(dppe)_2_] (Rh^0^) and [Rh(dppe)_2_]^−^ (Rh^−I^) congeners has been studied previously *via* chemical and electrochemical reactions.^25^ The chemical reduction of Rh^I^ using activated magnesium under strictly anhydrous conditions leads to Rh^−I^ resulting in the formation of CO under CO_2_ atmosphere.^24^ On the other hand, the reaction of the Rh(i) hydride [RhH(dppe)_2_]^0^ (Rh^I^H) with CO_2_ selectively yields the formate anion [Rh^I^][HCO_2_].^26^ From these and similar *ex situ* studies, the intermediates during electrocatalytic turnover have been plausibly inferred. However, the formation of the Rh^I^H hydride complex upon reduction of Rh^I^ has been the subject of insightful debates in the community.^27–30^ Pilloni and coll. stated a two-electron reduction of Rh^I^ into Rh^−I^, which was proposed to generate Rh^I^H by deprotonation of the solvent (EEC-mechanism; E and C: electrochemical and chemical steps, respectively; Fig. 1A, *q* = 2).^27,28^ Eisenberg and coll. hypothesised an alternative route involving a one-electron reduction to [Rh(dppe)_2_] (Rh^0^) followed by hydrogen atom abstraction from acetonitrile leading to the formation of Rh^I^H, while the second electron transfer converts the resulting solvent radical into the anion (ECE-mechanism; Fig. 1A, *q* = 1).^30^ The same group found later that the chemical reduction of Rh^I^ proceeds in two reduction steps leading to Rh^−I^ that subsequently slowly converts to Rh^I^H in the presence of acetonitrile,^25^ in line with the sequence initially reported by Pilloni and coll. In our recent systematic study on the bielectronic reduction of [Rh(dppe)_2_]^+^ and derivatives,^31^ we also found indications of irreversible reactions at the Rh^−I^ stage with electrophiles in the reaction media. While these works allow drafting first mechanistic hypotheses, the sequence of reduction and protonation steps, the nature of the proton source, and the competition between *ET*_*H*_ and *ET*_*M*_ pathways for CO_2_ reduction are still lacking a detailed picture (Fig. 1B).

In this regard, NMR spectroscopy offers a high chemical selectivity, but has resolution and quantitativity impeded by electrically conductive components present in the sample space that alter the static magnetic field *B*_0_ and radio frequency field of the excitation pulses resulting in spatial variations of flip angles. Therefore, NMR has remained underexploited for *operando* analysis of electrochemical processes.^32^ More recently, specifically adapted cell designs have mitigated these inherent challenges, to open up new possibilities of SEC-NMR.^32–42^ Complementary to other *in situ* techniques, which often focus on the electrode vicinity,^43–45^ SEC-NMR enables a survey of the bulk analyte. This feature is central to study secondary or slow catalytic processes^46^ or to image exchange processes or pH as a function of electrode distance, as demonstrated for CO_2_ electrolysis.^47,48^ Up to now, however, liquid state SEC-NMR has primarily been used for redox studies of organic molecules.^14,32,35^ Only few SEC-NMR studies trace the redox behaviour of metal complexes, namely ferrocene and potassium ferrocyanide,^11,12^ leaving this field of research underexplored in homogeneous electrocatalysis.

Here, we demonstrate the general qualitative potential of SEC-NMR to elucidate electrocatalytic reaction mechanisms by deciphering in detail the reaction network involving proton and carbon dioxide electroreduction using Rh^I^ (Fig. 1C).

## Results and discussion

### Basic reduction behavior in the absence of added proton source

Recent reinvestigations of the electrochemical behaviour of Rh^I^ by some of us showed a reversible reduction wave at a halfwave potential *E*_1/2_ = −2.12 V *vs.* Fc^+/0^ (noted V_Fc_). This reduction was assigned to the transfer of two electrons at inverted potentials (*E*^0^(Rh^I/0^) < *E*^0^(Rh^0/−I^)) with concomitant rearrangement of the coordination geometry from square planar in Rh^I^ to tetrahedral in Rh^−I^ (more background is provided in the reference).^31^ To obtain a more precise molecular picture of the processes underlying Rh^I^/Rh^−I^ reduction, we resorted to SEC-^31^P NMR experiments.

**Fig. 2:**
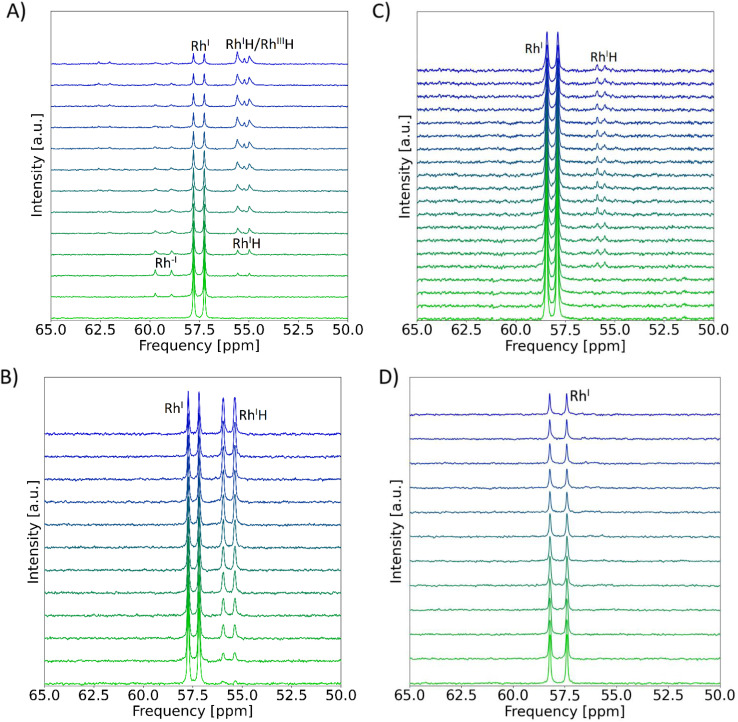
SEC-^31^P NMR measurements during electrolysis of [Rh(dppe)_2_]NTf_2_ at −0.4 mA; *n*Bu_4_NPF_6_, glassy carbon working electrode. The gradual colour change of the NMR spectra refers to measurements at proceeding time from *t* = 0 min (green) to *t* = 120 min (blue). Conditions: (A) Ar, MeCN-d_3_/THF-d_8_, spectra recorded every 10 minutes. (B) Ar, H_2_O (2 M), THF-d_8_, spectra recorded every 11 minutes. (C) CO_2_, MeCN, spectra recorded every 5 to 10 minutes. (D) CO_2_, H_2_O (2 M), THF-d_8_, spectra recorded every 7 to 15 minutes; the experiment was performed on a 400 MHz spectrometer. MeCN was used as solvent for comparison with CVs and preparative electrolysis. THF was added as co-solvent or used as solvent to prevent the precipitation of Rh^I^H in pure MeCN.

The experiments were performed in MeCN-d_3_, using *n*Bu_4_NPF_6_ as electrolyte salt and THF-d_8_ as co-solvent in a 1 : 1 ratio (Fig. 2A) to ensure good solubility of the Rh^I^H complex. Before electrolysis, the pristine solution exhibited an NMR doublet centered at *δ*(^31^P) = 57.47 ppm (^1^*J*_Rh,P_ = 133.2 Hz) as typical signature of the starting Rh^I^ complex. Applying a constant cathodic current (−0.4 mA) led to the gradual decrease of the integral of that signal, evidencing consumption of Rh^I^ (SI, Fig. S9). The build-up of a doublet at *δ*(^31^P) = 59.27 ppm with a characteristic ^1^*J*_Rh,P_-coupling constant of 203.2 Hz^25,31^ (Fig. 2A, S8 and 9) testifies to the formation of the Rh^−I^ complex as further corroborated by chemical reduction experiments (SI, Fig. S45(a and b)).

In subsequent spectra, the disappearance of this signal and the simultaneous evolution of a doublet at *δ*(^31^P) = 55.22 ppm with ^1^*J*_Rh,P_ = 142.7 Hz, diagnostic of the Rh^I^H complex,^25^ trace to the generation of this hydride species from the two-electron reduced Rh^−I^ complex (Fig. 2A, S8 and 9). *Ex situ* analysis of the isolated reaction mixture after 2 hours of electrolysis evidenced a ^1^H resonance at *δ*(^1^H) = −10.71 ppm assigned to the previously identified Rh^I^H (SI, Fig. S11 left).^25^ Over extended electrolysis time (after *ca.* 1 hour) an additional slightly shifted doublet at *δ*(^31^P) = 55.04 ppm with a much weaker Rh–P *J*-coupling (^1^*J*_Rh,P_ ≈ 103.4 Hz, SI Section 5.2.3) built up in the SEC-^31^P NMR, which can be assigned to [RhH(dppe)_2_(CD_3_CN)]^2+^ (Rh^III^H) based on literature data.^49^ This interpretation is consolidated by SEC-^1^H NMR spectra that revealed a signal in the hydride region at *δ*(^1^H) = −15.82 ppm, attributed to Rh^III^H (SI, Fig. S10) and by *ex situ* analysis of the isolated reaction mixture after 2 hours identifying Rh^III^H as predominant hydride species in the reaction mixture (SI, Fig. S11). We posit that the formation of Rh^III^H arises from the oxidation of Rh^I^H formed at the cathode by diffusion to the anode counter electrode of the SEC-NMR cell.

Assuming quantitativity to first approximation, the cumulative integrals of all ^31^P NMR resonances in the 43–63 ppm window after 2 hours amounted to 74% of the starting value. This decay in ^31^P NMR-active signals may be due to the formation of intermediates in low concentrations remaining under the detection limit, small amounts of paramagnetic compounds or to the electrodeposition of complex. A Faradaic efficiency (F. E.) of 11% for the evolution of Rh^−I^ is furthermore estimated from ^31^P NMR integration (SI, Section 5.3). While this value appears low, neither crossover nor catalytic turnover, *viz.* HER (*vide infra*), are accounted for.

Additional spectroelectrochemical analysis using SEC-UV/Vis at potentials negative to Rh^I^ reduction further corroborated the evolution of Rh^−I^ and Rh^I^H through partially convoluted signatures and the lack of detectable amounts of Rh^0^ (SI Section 5.1.2, Fig. S30 and Table S1).

**Fig. 3:**
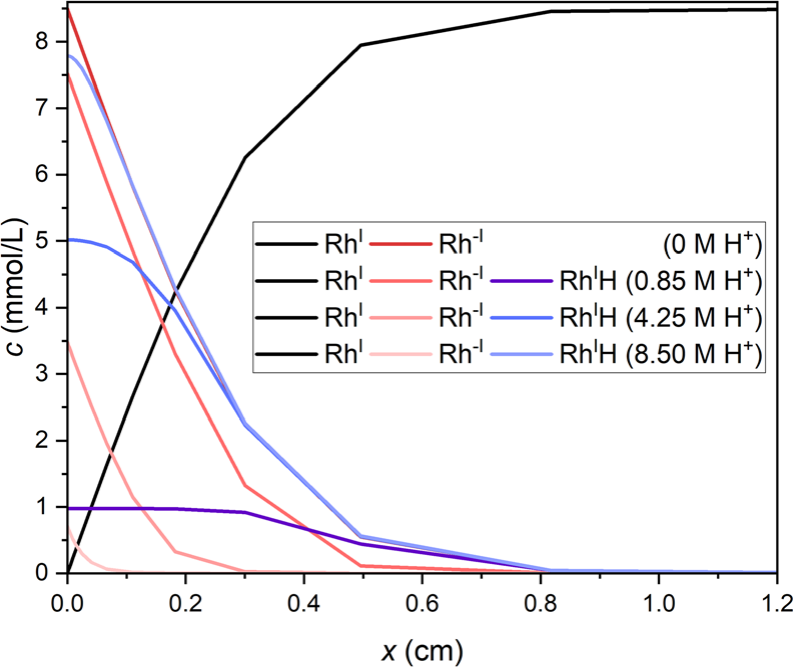
Simulated concentration profiles of Rh^I^ (black under all conditions), Rh^−I^ (red) and Rh^I^H (blue) extending from the electrode surface (*x* = 0) into the bulk during controlled potential electrolysis of Rh^I^ (8.5 mM) at −2.5 V_Fc_ after 120 min. Increasing proton concentrations are indicated by fading colour.

The concentration profiles for the Rh species evolving during controlled potential electrolysis were modeled using a software for electrochemical simulation (DigiElch from ElchSoft) to visualise concentration profiles in the bulk, as monitored by SEC-NMR. Alongside plausible reaction steps and concentrations of introduced species, kinetic and thermodynamic parameters obtained from previous estimations^50–53^ were fed as input (SI Section 8). Concentration profiles for Rh^I^, Rh^−I^ and Rh^I^H extending from the electrode surface (*x* = 0) into bulk (*x* > 0) after 120 minutes simulated electrolysis are shown in Fig. 3, assuming various possible concentrations of H^+^ for the protonation of Rh^−I^ to Rh^I^H.

First, the simulations indicate that the singly reduced Rh^0^ remains below relevant concentrations during electrolysis (SI, Fig. S50–57), corroborating results from SEC-UV/Vis. In the aprotic case ([H^+^] = 0 M), the doubly reduced Rh^−I^ complex is, as expected, the only species obtained from Rh^I^ at the electrode and diffusing into the bulk (Fig. 3 and S50). Simulations at increasing proton concentrations ([H^+^]/[Rh^I^] = 0.1 : 1, 0.5 : 1 and 1 : 1; Fig. 3 and S51–56) were performed using a rate constant for hydride formation of *k*_f_ = 2.3 s^−1^ derived from cyclic voltammetry (see SI Sections 4 and 10.1 for extraction of *k*_f_). Already with substoichiometric proton concentrations, the hydride complex Rh^I^H becomes clearly observable in the bulk of the medium after 20 minutes (SI, Fig. S51), in agreement with SEC-^31^P NMR spectra at reaction times >20 minutes. Experimentally, Rh^−I^ and Rh^I^H were detected in a concentration ratio of 1 : 5.6 in SEC-^31^P NMR spectra after two hours (SI, Fig. S9). Notably, Rh^I^H is not observed at short reaction times <10 minutes by SEC-^31^P NMR, thus suggesting protons gradually abstracted from the mixture components over the course of the electrolysis rather than initially present from protic impurities. Comparing the experimental ratio of Rh^−I^ and Rh^I^H integrals with the respective integrals in simulated concentration profiles at low [H^+^], we roughly estimate an apparent proton concentration in the electrolyte solution of 4 mM in our experimental conditions (SI, Fig. S54, 55 and Table S2). This concentration can be understood as a time-averaged proton concentration released and accumulating in the analyte over 120 min electrolysis. Within the first 10 minutes of electrolysis, the experimental proton concentration is lower than this estimated concentration, while it is expected to be higher after 120 minutes.

**Fig. 4:**
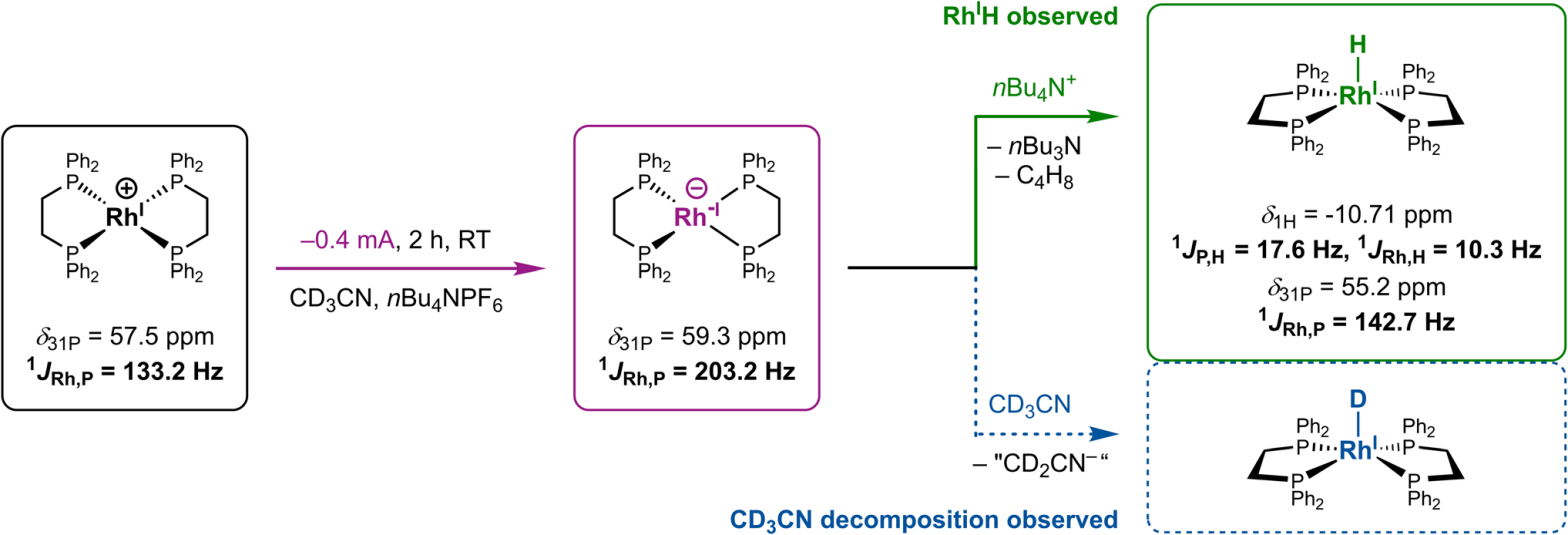
Formation of the rhodium hydride complex [RhH(dppe)_2_] during electrolysis of [Rh(dppe)_2_]NTf_2_ at −0.4 mA in a MeCN-d_3_/THF-d_8_/*n*Bu_4_NPF_6_ electrolyte as possible proton (*n*Bu_4_N^+^) and deuterium (CD_3_CN) source. Of note, although Rh^I^D could not directly be observed here, reductive decomposition of acetonitrile was traced by ^1^H NMR spectroscopy, presumably giving rise to deuteron liberation from CD_3_CN upon formation of a putative “CD_2_CN^−^” species and subsequent deuteration of Rh^−I^ into Rh^I^D (dotted blue).

We then investigated the origin of the protons involved in Rh^I^H formation by deuterium labelling in SEC-NMR. Electrolysis performed with deuterated acetonitrile, CD_3_CN, led to the observation of hydride signals in the ^1^H NMR spectrum (*vide supra*). In addition, the corresponding ^2^H NMR spectrum only shows solvent signatures and no detectable peaks in the hydride region (SI, Fig. S12). These observations suggest ^1^H-protonation of Rh^−I^ to [RhH(dppe)_2_] instead of formation of a [RhD(dppe)_2_] complex by putative deprotonation of CD_3_CN under our electrochemical conditions (Fig. 4, green reaction). Despite efforts to increase the detection limit of ^2^H NMR experiments (see SI, Section 5.2.1), we can yet not unambiguously exclude the presence of a deuteride complex and thus the role of CD_3_CN as a proton source purely based on ^2^H NMR.

At the same time, the *ex situ*^1^H NMR spectrum recorded after electrolysis features characteristic olefinic signals assigned to 1-butene, (*E*)/(*Z*)-2-butenes and not further specified species presumably forming upon reductive degradation of CD_3_CN (SI, Fig. S13, S41 and Section 5.2.3).^54,55^ The earlier species most plausibly result from the Hofmann elimination of the ammonium cation *n*Bu_4_N^+^ of the electrolyte, yielding tributylamine, butene and a proton, while the later species are suspected, but not evidenced within this work, to liberate protons and thus contribute to hydride formation (Fig. 4, blue reaction; SI, Fig. S41–44 and Section 5.2.3).

These data collectively support that, under our electroreductive conditions, Rh^I^H forms by reaction of the basic Rh^−I^ with protons originating from cathodic degradation of both *n*Bu_4_NPF_6_ and MeCN. Anodic processes providing additional protons were not further considered here. This result corroborates a previous study by Sofranko *et al.* showing that protons liberated by the decomposition of *n*Bu_4_N^+^ in benzonitrile react with Rh^−I^ to form Rh^I^H and aligns with reports on MeCN serving as proton source particularly when used with perchlorate-based electrolytes (SI Section 5.2.3).^25,30,56,59,60^ As a side note, despite carefully anhydrous conditions, residual water (<30 ppm, H_2_O/Rh^I^ < 1 : 5) can marginally contribute to the formation of Rh^I^H.

Spontaneous Hofmann elimination of the quaternary ammonium cation is a well-documented proton source under electrochemical conditions.^57,58^ However, in the absence of the Rh^I^ complex, olefinic butene signals are not detectable in the corresponding *ex situ*^1^H NMR spectrum (SI, Fig. S29). Thus, direct attack by Rh^−I^ at a β-H of *n*Bu_4_N^+^ appears to be an operating pathway for Rh^I^H formation under these conditions.

### Reactivity with added water

We next investigated the effect of water on the electroreductive behaviour of Rh^I^ in THF-d_8_. Upon electrolysis with added water (2 M), SEC-^31^P NMR spectra show the evolution of the Rh^I^H complex at 55.67 ppm aside from the signature of the Rh^I^ starting complex at 57.45 ppm (Fig. 2B, S16 and S19). SEC-^1^H NMR and *ex situ*^1^H NMR spectra confirm the presence of Rh^I^H with the diagnostic resonance at −10.56 ppm (SI, Fig. S17 and S18). Of note, Rh^−I^ is not observed here likely due to a fast protonation of this highly reactive intermediate into Rh^I^H in the protic environment. The transient nature of Rh^−I^ under these reaction conditions is also supported by simulated concentration profiles showing no detectable concentrations in bulk at high H^+^ concentrations (SI, Fig. S57). In turn, substantial conversion of the initial complex leads to a 1 : 1 Rh^I^/Rh^I^H ratio at extended SEC-NMR electrolysis time (SI, Fig. S19).

**Fig. 5:**
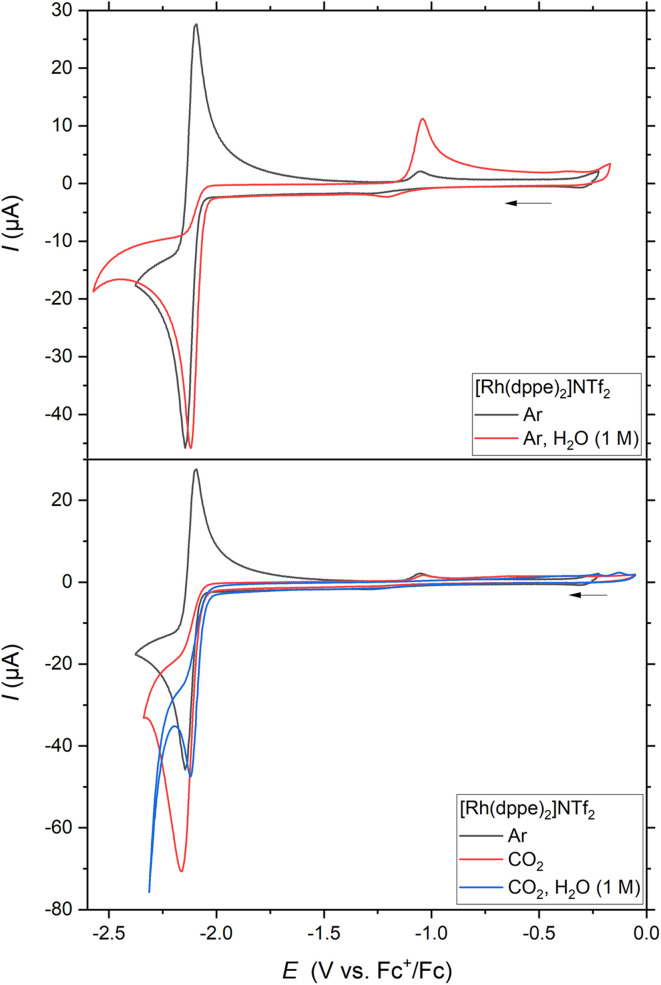
Cyclic voltammograms of [Rh(dppe)_2_]NTf_2_. Conditions: 1 mM Rh^I^ in MeCN, 0.2 M *n*Bu_4_NPF_6_, glassy carbon working electrode, 100 mV s^−1^.

The fast formation of Rh^I^H from the reduced Rh^−I^ complex is also corroborated by a complete loss of reversibility for the voltammetric reduction of Rh^I^ and the positive shift of the cathodic peak potential by 26 mV in the presence of water (Fig. 5, top). The anodic wave at −1.04 V_Fc_ in the backward scan is further attributed to the oxidation of Rh^I^H most likely to Rh^III^H as detected above in SEC-NMR (see Fig. 2A, S10 and 11).

We as well diagnosed hydrogen evolution reaction (HER) from the generated H_2_ signal at 4.54 ppm in the SEC-^1^H NMR spectra (SI, Fig. S17, right).^61–64^ Likely, HER arises from the protonation of Rh^I^H. However, the accumulation of Rh^I^H in the bulk of the SEC-cell indicates that this protonation is not very fast. In agreement, the lack of electrocatalytic current enhancement and the observation of Rh^I^H oxidation (Fig. 5, top) both suggest a relatively high stability of the hydride species *vs.* water as the proton source. Fitting the experimental CV with simulated models allows for an estimation of the reaction rate constant for HER of *k*_HER_ ≈ 0.06 s^−1^ (SI, Section 8, Fig. S59 and S60), substantially slower than hydride formation (*k*_f_ = 2.3 s^−1^; SI, Sections 4 and 10.1).

### Reactivity *versus* CO_2_ in the absence of added proton source

Exploring the high nucleophilicity of the reduced Rh^−I^ species in electrocatalysis, we engaged SEC-NMR measurements during the electrolysis of Rh^I^ under CO_2_ atmosphere. In the absence of added water (Fig. 2C and S20, left), SEC-^31^P NMR shows that the Rh^I^ d^8^ complex is the prevalent species in the bulk solution, whereas the reduced Rh^−I^ complex is not detected. CO_2_ is converted during the electrolysis, as supported by the decreasing integral of the corresponding ^13^C NMR signal (SI, Fig. S23). These observations point to a fast reaction of the reduced Rh species with CO_2_ under these conditions. Cyclic voltammetry supports this hypothesis, as the presence of CO_2_ turns the wave attributed to the Rh^I/−I^ couple into an irreversible event of catalytic character (Fig. 5, bottom), matching literature.^23^ The swift reductive disproportionation (RD) of CO_2_ to CO_3_^2−^ and CO reported at Rh^−I^ also consolidates this interpretation.^24^

After *ca.* 20 minutes, the formation of trace Rh^I^H is indicated by a tenuous doublet at 55.68 ppm, likely resulting from the reaction of Rh^−I^ with protons of the electrolyte (*vide supra*). The presence of this compound can here either lead to HER as previously observed or may yield HCOO^−^ by CO_2_ reduction *via* a hydride pathway.^3,4,65^ Competing conversions of CO_2_ and H^+^ are indeed observed by headspace gas chromatography (GC) analysis of a bulk electrolysis that shows the evolution of CO together with H_2_ (F.E. 16.2 ± 5.7% and 51.4 ± 2.0%, respectively).

### Reactivity *versus* CO_2_ with added water

When H_2_O is added to the reaction mixture under CO_2_ atmosphere, the evolution of Rh^I^H aside Rh^I^ is not apparent anymore in SEC-^31^P NMR spectra over 2 hours (Fig. 2D). *Ex situ* analysis with improved signal-to-noise ratio after electrolysis only traces the presence of Rh^I^H in low concentrations (Fig. S20, right and S22). This high Rh^I^/Rh^I^H ratio relative to the experiment performed under Ar indicates that the reactivity of Rh^I^H is exacerbated in presence of CO_2_, as also inferred from the loss of the voltammetric oxidation wave of Rh^I^H in CV (Fig. 5, bottom).

Two hypotheses can be raised to account for this behaviour that likely overlap: (a) Rh^I^H is more reactive towards CO_2_ insertion than towards protonation by H_2_O, leading to HCOO^−^ as previously reported^65^ and observed under purely chemical conditions (Fig. S47); (b) the presence of CO_2_ generates a more acidic proton source in the form of H_2_CO_3_, speeding up protonation of Rh^I^H and hence HER. These competing reactivities of Rh^I^H are apparent from formate signals in e*x situ*^1^H and ^13^C NMR spectra after SEC-electrolysis (SI Fig. S25) and from H_2_ detection in the gas phase of a bulk electrolysis experiment (F.E. 69.7%). Only small amounts of CO (F. E. 6.2%) were also detected in the gas phase, while formate formation was assumed but could not unambiguously be confirmed in the performed bulk experiments.

The redirection of the reactivity *versus* CO_2_ in the presence of water is also reflected by the loss of cathodic peak current enhancement in CV and the anodic shift of the reduction wave by 26 mV. The effect of added water likely arises from the preference of protonation over CO_2_ coordination at the Rh^−I^ complex. However, subsequent reaction steps in the *ET*_*H*_ pathway can overall decelerate kinetics for the electrocatalytic cycles.

### Discussion of mechanistic pathways

**Fig. 6:**
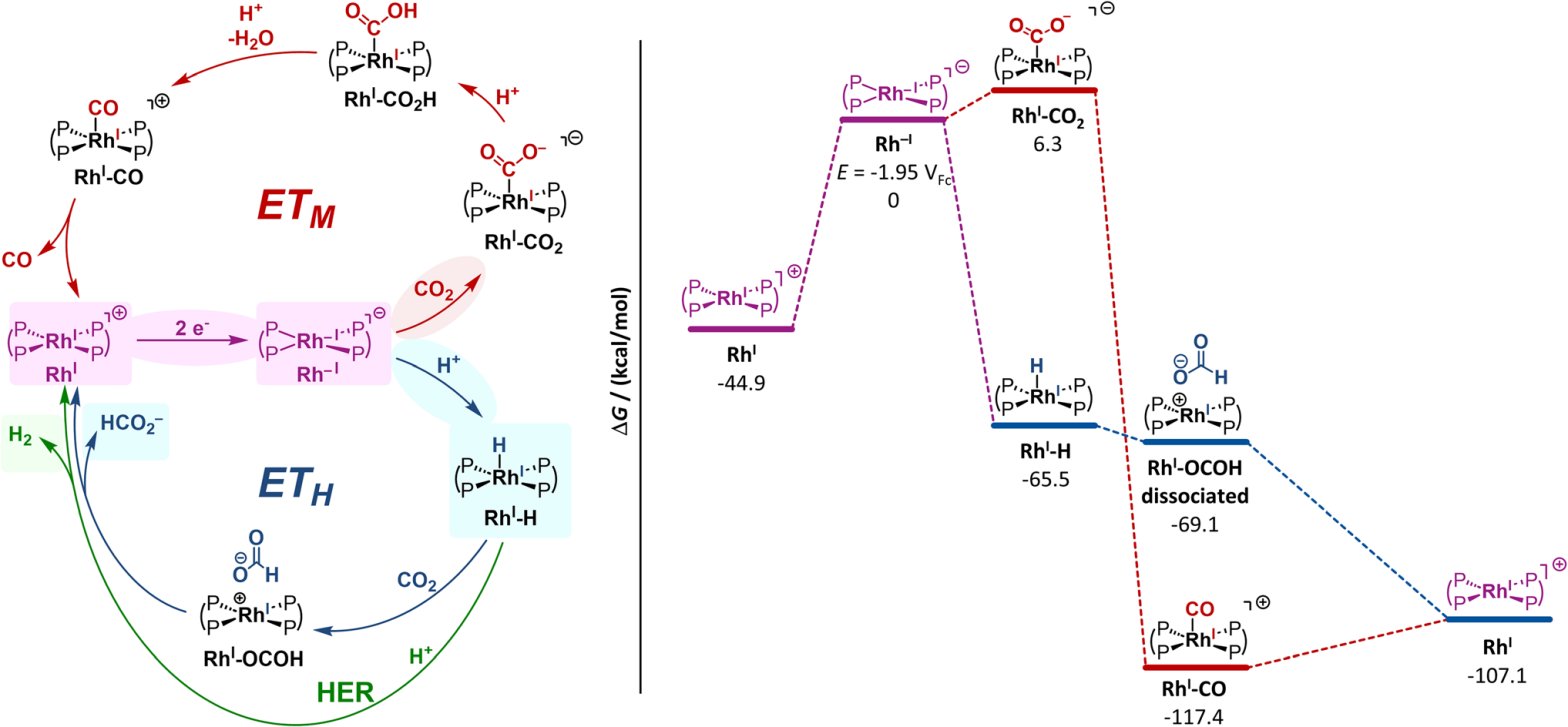
Left: Possible reaction pathways for electrocatalytic reactions from Rh^I^ with H^+^ and CO_2_ following general *ET*_*M*_, *ET*_*H*_ and HER mechanisms. Highlighted intermediates, reactions and products were identified by (spectro)electrochemical experiments. Right: Energy profiles for the electrocatalytic reduction of CO_2_ following the *ET*_*H*_ (blue) or the *ET*_*M*_ (red, water as byproduct is not depicted here) pathways. Only energies of intermediates are depicted. Energies were computed with uMN12L/def2-TZVP in acetonitrile and are given in kcal mol^−1^.

Based on our experimental observations, we further documented possible *ET*_*H*_ and *ET*_*M*_ reaction pathways for the electrocatalytic activation of CO_2_ at Rh^−I^ with energy profiles computed by density functional theory (DFT) methods (Fig. 6, S48 and 49).^4^ As the observed electrocatalytic turnover at room temperature already indicates reasonably accessible energy barriers, we did not perform time-consuming transition state computations within this work, but only focused on intermediate state energies to retrace the thermodynamic driving force of this reactivity. We also excluded, as a first approximation, pathways involving the singly reduced Rh^0^ congener, due to the lack of experimental evidence for the formation of this species and the reported low reactivity of Rh^0^ contrasting the high one noticed for Rh^−I^.^25^

In the *ET*_*M*_ pathway, the initial attack of CO_2_ by Rh^−I^ resulting in the η^1^-CO_2_ complex Rh^I^-CO_2_ is slightly endergonic by 6.3 kcal mol^−1^. Under protic conditions, the protonations following Rh^I^-CO_2_ and yielding water and the carbonyl complex Rh^I^-CO are computed as strongly driven. Yet, whether or not water is added, Rh^I^-CO_2_, Rh^I^-CO or other intermediates of the *ET*_*M*_ sequence are not detected in the SEC-NMR. This fact suggests that either *ET*_*M*_ is not operative or, if this pathway is operative, CO readily dissociates from the pentacoordinated complex into the gas phase forming the square planar 16-electron complex Rh^I^ as most abundant reaction intermediate. Our data, in agreement with previous reports,^24^ yet indicate that a reductive disproportionation leading to CO and CO_3_^2−^ is favoured in aprotic conditions and the cumulated rates of the *ET*_*M*_/RD mechanism are high enough to lead to the marked electrocatalytic behaviour observed in voltammetry. In protic conditions, involvement of [Rh^I^(dppe)_2_(CO_2_)]^−^ as entry to the *ET*_*M*_ mechanism cannot be discarded but is less favoured as apparent from smaller amounts of CO as reduction product of CO_2_ detected after electrolysis.

In the alternative *ET*_*H*_ mechanistic cycle, the initial formation of the rhodium hydride Rh^I^H from Rh^−I^ is highly exergonic (−65.5 kcal mol^−1^) in line with experimental observations (*vide supra*). The reaction of this intermediate with CO_2_ or H^+^ would then lead respectively to HCOO^−^ (in a dissociated complex) or H_2_ in competing HER and eventually to Rh^I^ in both cases. These downstream reactions are all computed exergonic and pinpoint to fast and driven chemical reaction steps, while the electrochemical reduction step requires higher energy input. Thus, DFT computations support Rh^I^ as resting state in agreement with SEC-NMR experiments showing this complex as major species during electrolysis in the presence of H_2_O and CO_2_.

Under experimental aprotic reaction conditions, Rh^I^H, which SEC-NMR reveals to be generated in low concentrations likely from Hofmann elimination of the electrolyte salt and deprotonation of MeCN (*vide supra*), can give rise to an operative *ET*_*H*_ route. Under protic conditions, the substantial change in the CV response indicates *ET*_*H*_ as the most favoured route. While the hydride complex readily forms, the kinetics for Rh^I^H conversion becomes fast enough that this intermediate does not accumulate in the bulk but slow enough that electrocatalysis is not observed at the voltammetric timescale. A switch from a predominant faster *ET*_*M*_/RD to slower *ET*_*H*_ as the concentration in proton source is increased also matches the loss in activity observed upon titrating water in the CV experiments (Fig. S33). Although turning over at much slower rates, the *ET*_*H*_ route diverts from or blocks the *ET*_*M*_/RD in virtue of the strong driving force to the Rh^I^H intermediate.

As a note, ECEC pathways involving further reduction of Rh^I^-CO_2_H, Rh^I^-CO and Rh^I^H have been discarded in a first approximation, due to the quite negative values computed for the corresponding potentials (*E*^0^(Rh^I^-CO_2_H/Rh^0^-CO_2_H) = −2.61 V_Fc_; *E*^0^(Rh^I^-CO/Rh^0^-CO) = −2.32 V_Fc_; *E*^0^(Rh^I^H/Rh^0^H) = −2.84 V_Fc_; SI, Fig. S48, 49 and Section 7).

## Conclusions

By SEC-NMR, we were able to characterise and monitor the two-electron reduction of [Rh(dppe)_2_]^+^Rh^I^ into [Rh(dppe)_2_]^−^Rh^−I^ followed by protonation to the hydride complex Rh^I^H under electrolysis conditions, according to an overall EEC mechanism. Isotope labelling experiments and *ex situ*^1^H NMR analysis after SEC-NMR revealed that the proton results from Hofmann elimination of the cation in *n*Bu_4_NPF_6_ electrolyte and from MeCN degradation under aprotic conditions. Under electrocatalytic CO_2_ reduction, Rh^I^ is the only observable species indicating fast reductive disproportionation at Rh^−I^ leading to CO as the preferred C_1_ product. In the presence of added water, formate is formed from CO_2_ in competition with H_2_ evolution. The inferred Rh^I^H as key intermediate can be detected by *ex situ* NMR and as main species under SEC NMR in presence of only water. Hydrogen evolution outperforms CO_2_ reduction reaching Faradaic efficiency of 69.7%, indicating that protonation of the Rh–H moiety is favoured over CO_2_ insertion. These results come in perspective of the high reactivity of rhodium hydride complexes towards CO_2_ insertion as a crucial step in thermo-catalytic CO_2_ hydrogenation towards formic acid/formates.

Our study hence highlights the analytical power of SEC-NMR to elucidate intermediates and mechanisms in organometallic electrocatalysis. The resulting molecular understanding can form the basis of further investigations aiming at integration of the *ET*_*M*_ pathway under electrochemical conditions with other organometallic catalytic cycles beyond C_1_ products.

## Author contributions

A.-C. K., M. S., J. G., N. K. and W. L. designed the project. A.-C. K. performed synthetic works, spectroscopic characterisations, CV studies, chemical reduction experiments and DFT computations. M. S. conducted spectroelectrochemical NMR measurements. C. K. performed spectroelectrochemical UV/Vis experiments and electrolysis. N. K. initiated and A.-C. K. completed DigiElch simulations. A.-C. K, M. S. and N. K. analysed the data. M. H., R.-A. E., J. G., N. K. and W. L. supervised the project. A.-C. K. and N. K. drafted the manuscript, which was edited by contributions of all authors.

## Conflicts of interest

There are no conflicts to declare.

## Supplementary Material

SC-017-D5SC05744B-s001

## Data Availability

UV/Vis and TopSpin raw data, DFT *xyz* and output files and GC data are available on the Jülich DATA repository (https://doi.org/10.26165/JUELICH-DATA/KJN10U). All other data that support the findings of this study are available from the corresponding authors upon reasonable request. Supplementary information: experimental procedures, (spectroelectrochemical) NMR and UV/Vis spectra, cyclic voltammograms and related plots for extraction of kinetic data, DFT computed energy profiles and simulated CV and electrolysis data are available in the SI. The authors have cited additional references within the SI.^25,28,30,31,40,50–53,56,59,60,66–80^ See DOI: https://doi.org/10.1039/d5sc05744b.
