# Supplementary material for: Mapping proton and carbon dioxide electrocatalytic reductions at a Rh complex by in situ spectroelectrochemical NMR
Source: Chem Sci. 2025 Nov 10;17(3):1637–46. doi: 10.1039/d5sc05744b (PMC12666655; doi:10.1039/d5sc05744b)
Supplement: SC-017-D5SC05744B-s001 [file SC-017-D5SC05744B-s001.pdf]

Supplementary Information (SI) for Chemical Science.
This journal is © The Royal Society of Chemistry 2025

Supporting Information

Mapping Proton and Carbon Dioxide Electrocatalytic Reductions at a Rh Complex by In Situ Spectroelectrochemical NMR

A.-C. Kick,[a,b] M. Schatz,[c,a] C. Kahl,[b] M. Hölscher,[a] R.-A. Eichel,[c,d,e] J. Granwehr,*[c,a] N. Kaeffer,*[b,f] W. Leitner*[a,b]

a) Institute of Technical und Macromolecular Chemistry, RWTH Aachen University, Worringerweg 2, 52074 Aachen, Germany.

b) Max-Planck-Institute for Chemical Energy Conversion, Stiftstraße 34-36, 45470 Mülheim an der Ruhr, Germany.

c) Institute of Energy Technologies, Fundamental Electrochemistry (IET-1), Forschungszentrum Jülich, Wilhelm-Johnen-Straße, 52428 Jülich, Germany.

d) Institute of Physical Chemistry, RWTH Aachen University, Landoltweg 2, 52074 Aachen, Germany.

e) Faculty of Mechanical Engineering, RWTH Aachen University, Eilfschornstraße 18, 52062 Aachen, Germany.

f) Université de Strasbourg, Université de Haute-Alsace, CNRS, LIMA, UMR 7042, 67000 Strasbourg, France.

Corresponding Authors:

*E-Mail for J.G.: j.granwehr@fz-juelich.de

*E-Mail for N.K.: nkaeffer@unistra.fr

*E-Mail for W.L.: walter.leitner@cec.mpg.de

Contents

1. GENERAL CONSIDERATIONS 3
2. CYCLIC VOLTAMMETRY 4
3. ELECTROLYSIS EXPERIMENTS 5
4. EXTRACTION OF CHEMICAL RATE CONSTANTS FROM CYCLIC VOLTAMMETRY 7
5. SPECTROELECTROCHEMICAL (SEC) EXPERIMENTS 8
   - 5.1 SEC-UV/VIS 8
     - *5.1.1 Experimental Procedure 8*
     - *5.1.2 Additional Discussion 8*
   - 5.2 SEC-NMR 9
     - *5.2.1 Experimental Set-up and Procedure 9*
     - *5.2.2 Notes on the Comparibility between SEC-NMR and Bulk Electrolysis 11*
     - *5.2.3 Additional Information and Discussion 12*
   - 5.3 FARADAIC EFFICIENCY 14
6. CHEMICAL REDUCTION EXPERIMENTS 16
7. DENSITY FUNCTIONAL THEORY METHODS 16
8. ELECTROCHEMICAL SIMULATIONS WITH *DIGIELCH* 18
9. SUPPORTING FIGURES: SPECTROELECTROCHEMISTRY 22
   - 9.1 POTENTIAL PROFILES FOR CONTROLLED CURRENT ELECTROLYSIS 22
   - 9.2 ELECTROLYSIS OF $[RH(DPPE)_2]^+$ UNDER INERT REACTION CONDITIONS 23
   - 9.3 ELECTROLYSIS OF $[RH(DPPE)_2]^+$ UNDER PROTIC CONDITIONS IN ARGON ATMOSPHERE 27
   - 9.4 ELECTROLYSIS OF $[RH(DPPE)_2]^+$ UNDER CO_2 ATMOSPHERE 29
   - 9.5 ELECTROLYSIS OF NEAT ELECTROLYTE 33
   - 9.6 SPECTROELECTROCHEMICAL UV/VIS 35
10. SUPPORTING FIGURES: CYCLIC VOLTAMMOGRAMS AND KINETIC PLOTS 36
   - 10.1 $[RH(DPPE)_2]NTF_2$ WITH H_2O (1 M) UNDER AR 36
   - 10.2 $[RH(DPPE)_2]NTF_2$ WITH H_2O (1 M) UNDER CO_2 37
   - 10.3 $[RH(DPPE)_2]NTF_2$ UNDER INERT CONDITIONS WITH VARYING ELECTROLYTES 38
11. SUPPORTING FIGURES: BULK ELECTROLYSIS 40
12. SUPPORTING FIGURES: UV/VIS SPECTRUM OF $[RHH(DPPE)_2]$ 41
13. SUPPORTING FIGURES: CHEMICAL REDUCTION EXPERIMENTS 42
14. SUPPORTING FIGURES: DENSITY FUNCTIONAL THEORY COMPUTATIONS 49
15. SUPPORTING FIGURES: DIGIELCH SIMULATION DATA 51
16. REFERENCES 58

1. General Considerations

All manipulations were performed under a dry argon atmosphere in a GS MEGA, a LABmaster PRO SP ECO and UNIlab plus ECO by MBraun gloveboxes or on a Schlenk line using standard techniques. Gases were purchased from AirLiquide, rhodium precursors were purchased from Johnson Matthey, and all further chemicals were purchased from Roth, Sigma-Aldrich and TCI, dried over molecular sieves (liquids) or by heating to 50 °C in high vacuum for at least 3 hours ($1\cdot10^{-3}$ mbar, solids) and degassed. No further purification was performed. Organic solvents were degassed by either purging with argon for at least one hour or by the freeze-pump-thaw method and were subsequently dried and stored over molecular sieve (4 Å). The water content after the drying procedure was checked by Karl Fischer titration (Metrohm KF756) and only solvents with a residual water content ≤ 30 ppm were used. Water was degassed by purging with argon for 2 hours. For chemicals purchased in dry and inert conditions, no further drying or degassing procedure was followed. Electrode materials for NMR spectroelectrochemical measurements and electrolysis experiments were purchased from HTW and Goodfellow as indicated below, electrodes and cells for spectroelectrochemical UV/vis experiments were purchased from BioLogic and electrodes for cyclic voltammetry were purchased from BioLogic (working and counter electrodes), ALS (non-aqueous reference electrode) and Innovative Instruments (leak-free reference electrode). The complexes di(1,2-bis(diphenylphosphino)ethane)rhodium(I)triflimid, hydridotetrakis(triphenyl-phosphine)rhodium(I) and hydridobis(1,2-(diphenylphosphino)ethane)rhodium(I) were synthesized according to reported procedures.[1-3] Unless stated differently, *in situ* and *ex situ* spectroelectrochemical NMR data are acquired as described in section 5.2. NMR spectroscopy data for chemical reduction experiments were obtained on a Bruker AS400-spectrometer or 500 MHz Bruker Avance III HD spectrometer with a Bruker Prodigy probe. All ^{1}H and ^{13}C NMR spectra were referenced to the residual proton of the NMR solvent, ^{31}P-spectra are relative to 85% phosphoric acid. Ultraviolet-visible measurements of [RhH(dppe)$_2$] recorded for reference were performed on an Autolab Spectrometer UB using a D/HAL light source. The program AvaSoft 8 was used for recording. The acquisition of spectroelectrochemical UV-Vis data is described in section 5.1.

2. Cyclic Voltammetry

Cyclic voltammograms (CVs) were conducted with an Autolab PGSTAT203 potentiostat using a three-electrode setup with a glassy carbon working electrode (3 mm diameter, polished with 0.05 µm alumina slurry), a platinum wire counter electrode and a $AgNO_3$/Ag reference electrode (0.01 M $AgNO_3$ in 0.1 M $n$$Bu_4NPF_6$/$CH_3CN$) mounted with a Vycor frit. The electrolyte solution was 0.2 M $n$$Bu_4NPF_6$ in MeCN unless otherwise noted. The electrolyte was purged with argon or CO_2 for 10 minutes. Background recordings confirmed the absence of oxygen impurities. After addition of $[Rh(dppe)_2]NTf_2$ (1 mM) to the purged electrolyte, the solution (total volume 6 mL) was purged for two more minutes. During the measurements, an Ar, or alternatively a CO_2 flow was maintained in the headspace of the cell. The CV measurements were started from the open-circuit potential in cathodic direction at a scan rate of 100 $mV{\cdot}s^{-1}$. After experiments under aprotic conditions, water (2 M) was added to the analyte solution and the CV recording was repeated. For potential referencing (Volt vs. the $Fc^{+/0}$ couple, abbreviated as V_{Fc}), ferrocene was added to the analyte solution at the end of each measurement. During CV measurements the ohmic drop was compensated by a value of 90 Ω. This value was determined by the positive feedback method implemented in the Autolab Nova Software.

Of note, one minor impurity with an oxidation potential of –0.18 V_{Fc} at low peak currents was detected in CV measurements of the blank electrolyte (Figure S 37).

3. Electrolysis Experiments

Controlled potential electrolysis experiments were conducted in a custom-made (Adams & Chittenden Scientific Glass) gas tight H-cell with two compartments separated by a P3 glass frit and connected headspaces (Figure S 1). The cell was assembled hot by inserting a hot reticulated vitreous carbon foam working electrode while purging with a flow of acetonitrile-saturated Ar. A platinum mesh counter electrode and a AgCl/Ag leak-free reference electrode were rinsed with ultrapure water and ethanol, dried in an Ar flush and inserted into the cell. Ace thread PTFE sleeves with O-rings for gas-tight electrode mounting and GL14 screw caps were used to seal the cell.

After installation of the electrodes, the cell was flushed with argon for 20 minutes prior to addition of the electrolyte solution. The catholyte (0.2 M *n*Bu_4NPF_6, 1 mM $[Rh(dppe)_2]NTf_2$ in 12 mL MeCN) and anolyte (0.2 M *n*Bu_4NPF_6 in 7 mL MeCN) were filled simultaneously in the respective compartments. The electrolyte solution was sparged with CO_2 for another 20 minutes. A cyclic voltammogram was recorded at a scan rate of 100 $mV \cdot s^{-1}$ to derive the non-referenced reduction potential of the rhodium complex for electrolysis.

The cell was sealed gas tight and the initial gas composition was determined by headspace gas chromatography by autosampled (Shimadzu AOC-6000) puncturing of a PTFE septum at the cathodic cell compartment with a gas-tight syringe and injection (400 µL, split ratio of 5.0, 250 °C) of the sample into a Shimadzu NEXIS GC-2030 with a CARBOXEN-1010 PLOT GC column (30 m x 0.32 mm) and flushed with Helium at 30 $cm \cdot s^{-1}$. The column temperature was maintained isothermal at 40 °C for 2 min, ramped to 200 °C at a rate of 10 $°C \cdot min^{-1}$ and maintained at 200 °C for 8 min. The thermal conductivity detector was operated at 100 °C and 100 mA. A Biologic SP-300 potentiostat with ±1 A/±48 V booster card was used for electrolysis. Controlled potential chronoamperometry with ohmic drop compensation (90 Ω, value determined from a ZIR measurement before electrolysis and compensated by 85% during electrolysis as implemented in the EC-lab software) were recorded for 4 hours. The catholyte headspace was analyzed by gas chromatography every 30 min. After completion of the electrolysis, the anolyte and catholyte solutions were removed from the cell and analyzed by NMR spectroscopy. To reference the potential, the cell was filled with

fresh electrolyte solution containing ferrocene (1 mM) and the equilibrium potential of ferrocene determined by CV.

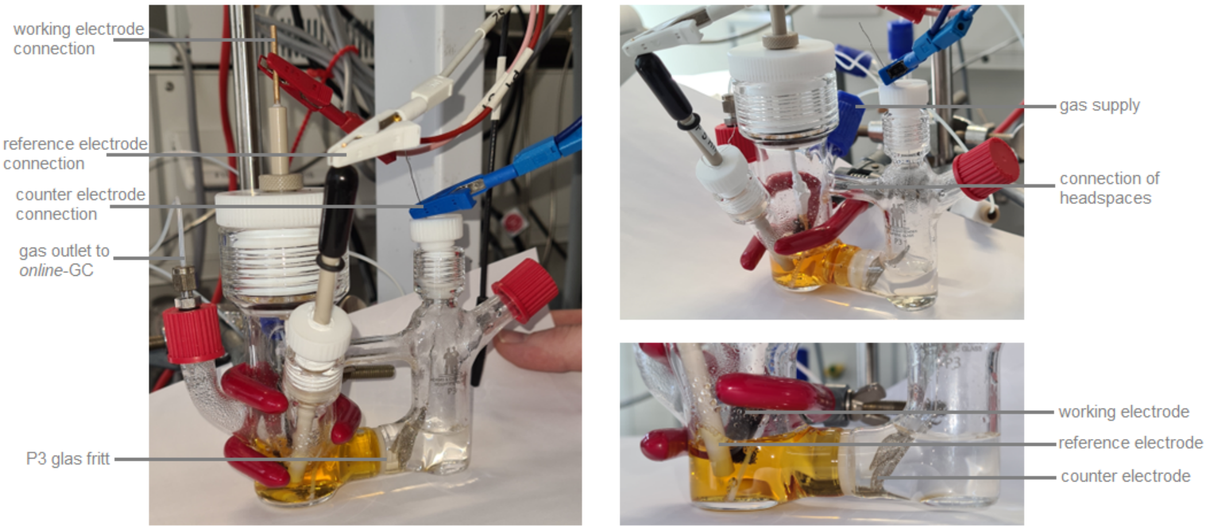

Figure S 1. Gas tight electrolysis cell with divided compartments used for controlled potential electrolysis of [Rh(dppe)$_2$]NTf$_2$ under CO_2 atmosphere.

4. Extraction of Chemical Rate Constants from Cyclic Voltammetry

Reaction kinetics for the reaction of **Rh**$^{-I}$ with H^+ were determined for solutions of **Rh**^{I} (1 mM) in acetonitrile with added water (1 M).

Considering reactions in the purely kinetic KP regime, reaction kinetics are defined by eq. 1 with n electrons transferred during the redox event at an equilibrium potential E^0 and at a temperature T. The corresponding rate constants k_f can be extracted from the shift of the cathodic peak potential E_p in by varying scan rate ν in cyclic voltammetry experiments. A plot of E_p vs. ln(ν) and application of a linear regression delivers the y-axis intercept b, from which k_f (= k_f'[H_2O]) can be determined according to eq. 2 using the faraday constant F and the universal gas constant R. Of note, two different kinetic regimes are apparent from the E_p vs. ln(ν) plot, where electron transfer kinetics dominates at high scan rates and chemical reaction kinetics rules at lower scan rates. Thus, k_f was determined for scan rates < 1 V·s^{-1}. Corresponding plots can be found in section 10.1.

$$E_p = E^0 - 0.78\frac{RT}{nF} + \frac{RT}{2nF}\ln\left(\frac{RTk_f}{nF\nu}\right) \quad \text{(eq. 1)}$$

$$\Rightarrow k_f = \frac{nF}{RT}\exp\left(1.56 + \frac{2nF}{RT}(b - E^0)\right) \quad \text{(eq. 2)}$$

5. Spectroelectrochemical (SEC) Experiments

5.1 SEC-UV/Vis

5.1.1 Experimental Procedure

Spectroelectrochemical UV/Vis measurements were carried out with an Agilent Technologies Cary 8454 UV/Vis spectrometer and a BioLogic SP-300 potentiostat.

The experiments were performed in a thin-film (light path: 1 mm) quartz cuvette, using a three-electrode set-up with a gold grid working electrode, a platinum wire counter electrode and $AgNO_3$/Ag reference electrode. The cuvette was filled with an MeCN analyte containing [Rh(dppe)$_2$]NTf$_2$ (0.25 mM) and *n*Bu$_4$NPF$_6$ (0.1 M) under argon sparging and the electrodes placed inside. The cuvette was then positioned in the UV/Vis device. Before electrolysis, argon sparging was moved to the headspace and a UV/Vis spectrum of the solution was measured for reference. Controlled potential electrolyses of the thin film with the gold grid electrode were then carried out at –2.28 V_{Fc} and UV-Vis spectra recorded every 0.5 seconds over a wavelength range of 200 nm to 1100 nm.

The SEC-UV/Vis spectra were subtracted from the reference absorbance obtained at resting conditions for [Rh(dppe)$_2$]NTf$_2$, $A_{Rh(I)}$, to obtain relative differential absorbance A_{rel}.

For UV/Vis measurements of [RhH(dppe)$_2$] (**Rh^{I}H**) recorded for reference (see section 11), cuvettes by Hellma (1 cm x 1 cm in size) were filled with a solution of **Rh^{I}H** (0.5 mg·mL^{-1}) in THF under argon atmosphere and sealed with a plug to maintain an inert atmosphere.

5.1.2 Additional Discussion

An additional insight into the processes related to the reduction of **Rh^{I}** was gained by SEC-UV/Vis measurements during electrolysis of **Rh^{I}** at a potential cathodic to the reduction of this complex in MeCN (E_{app} = –2.28 V_{Fc}; Figure S 30). First, a net decrease of the absorbance at 239 nm confirms the consumption of **Rh^{I}**. The co-evolution of bands at 283 nm and 345 nm, which are well matching specific signatures of **Rh^{I}H** as confirmed by reference measurements on the isolated complex, indicates the concomitant formation of that hydride species (Figure S 40; Table S 1). The differential

spectra are also marked by local minima at 317 nm and 403 nm. We attribute this observation to the fact that both disappearing **Rh^{I}** and forming **Rh^{I}H** exhibit absorbance maxima very close to these wavelengths. Additionally, a broad band at 444 nm suggests the formation of the two-electron reduced **Rh^{-I}** complex.[4] At contrast, no absorbance is observed at wavelengths longer than 600 nm, thusly discarding the substantial accumulation of the neutral blue [Rh(dppe)$_2$] complex.[4] Therefore, these results are in agreement with the two-electron reduction of **Rh^{I}** to **Rh^{-I}** and the formation of a rhodium(I) hydride complex.[4] The lack of **Rh^{0}** traces corroborates potential inversion in the reduction sequence,[1] but a fast H-atom abstraction from MeCN at the transient **Rh^{0}** (ECE mechanism) cannot be fully excluded.[5]

5.2 SEC-NMR

5.2.1 Experimental Set-up and Procedure

Unless stated differently, SEC-NMR experiments were conducted using a Bruker DiffBB broadband gradient probe on a Bruker Avance III HD spectrometer with a 14.1 T wide-bore magnet, corresponding to a ^{1}H resonance frequency of 600.3 MHz. SEC-NMR experiments conducted on a Bruker DiffBB broadband gradient probe on a Bruker Avance III HD spectrometer with a 9.4 T wide-bore magnet, corresponding to a ^{1}H resonance frequency of 400.2 MHz are indicated. The electrochemical cell was designed to fit into a standard 5 mm NMR tube for which this probe was designed (Figure S 2). A SIGRADUR® glassy carbon rod with 1 mm outer diameter (HTW Hochtemperatur-Werkstoffe GmbH, Thierhaupten, Germany) served as working electrode (WE). An isolated platinum wire (GoodFellow GmbH, Hamburg, Germany) stripped at a length of ca. 4 mm and placed close to the WE served as reference electrode (RE). The counter electrode (CE) was a 3x4 mm platinum mesh (GoodFellow GmbH, Hamburg, Germany) connected to the stripped end of another platinum wire. All electrodes were placed outside of the sensitive NMR volume, with WE and RE placed above and the CE underneath it (Figure S 2).

500 µL of the analytic solution containing [Rh(dppe)$_2$]NTf$_2$ (8.5 mM) and *n*Bu$_4$NPF$_6$ (0.1 M) in acetonitrile and/or tetrahydrofuran was filled into an NMR tube, the electrodes were introduced and the cell was closed and sealed under Ar atmosphere

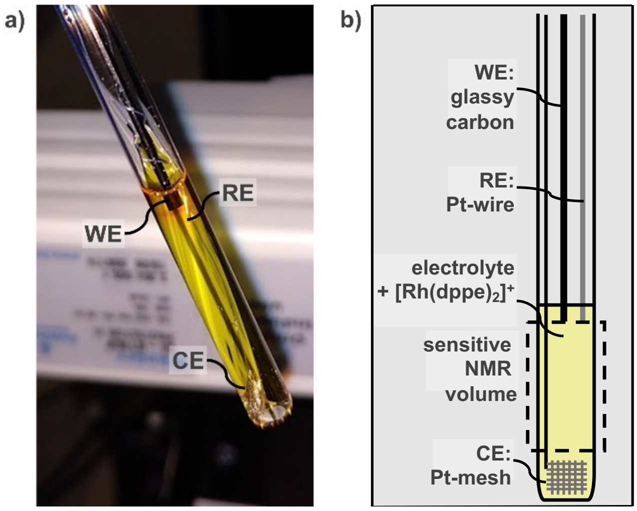

Figure S 2. A) Photograph of SEC-NMR cell in NMR tube under reductive conditions. B) Schematic of the cell.

In a glove box. For reactions under CO_2 atmosphere, the NMR tube was positioned in a Schlenk tube and the argon atmosphere exchanged against a $^{13}CO_2$ atmosphere by bubbling the gas into the solution. If required for the respective experiment, H_2O (20 µL, corresponding to 2 M as a common acid concentration in organometallic electrocatalysis) was added to the solution using a microliter syringe, while the sample was in the Schlenk tube. The NMR tube was inserted into and connected to a cell holder that connects the three electrodes to a BioLogic SP-200 potentiostat (BioLogic Science Instruments, Seyssinet-Pariset, France). The design of the cell holder as well as the measures to minimize the influence of environmental noise and conductive parts in the sensitive NMR volume are described in detail in a previous publication.[6]

The cell was inserted into the NMR probe and the electrolysis was initiated by chronopotentiometry at a current of –0.4 mA. Simultaneously, the ^{31}P resonance was continuously recorded. The 30°-pulses had a length of 6 µs at 39 W pulse power. For ^{1}H decoupling a WALTZ-16 sequence was used. 128 repetitions with a delay of 2 s resulted in a temporal resolution of approximately 5 mins. Resulting spectra were processed with 5 Hz line broadening. ^{1}H and ^{13}C spectra were recorded using 90°-pulse experiments without decoupling. ^{2}H spectra were recorded using 30°-pulse experiments to increase the excitation bandwidth, covering the hydride region. For improved detectability of a putative deuteride complex in ^{2}H NMR spectra, additional experiments with 90°-pulse experiments with low bandwidth in the hydride region and multiple-scan experiments were performed. For more details, please refer to the NMR data files on the Jülich DATA repository.

As the working electrode potential could not reliably be measured in the SEC-NMR cell, electrolysis was performed at controlled current using a Pt pseudo-reference electrode instead of a genuine reference electrode. The minimal current demand to initiate reduction of the rhodium complex was estimated from initial CV experiments at 200 $mV \cdot s^{-1}$. Fine-tuning of the required current for electrolysis adjusting reaction kinetics to the detectability of Rh species was then performed in preliminary SEC-NMR electrolysis experiments.

5.2.2 Notes on the Comparibility between SEC-NMR and Bulk Electrolysis

The magnetohydrodynamic effect is expected to enhance solution mixing during SEC-NMR resulting in transport of species formed from electrode processes into the sensitive NMR volume during electrolysis. Along this convective mixing, the comparability of SEC-NMR experiments to bulk electrolysis is increased, but still remains impeded by significant differences in cell design and mixing.[7]

Although changes in viscosity and diffusion during SEC-NMR were not evaluated in this work, the effect of chemical inhomogeneities, that can additionally result from improper mixing inside the NMR tube, is considered minor compared to the inhomogeneity caused by introduction of electronically conductive materials into the NMR sensitive volume.

Finally, we want to address the aspect of electrolysis operation mode and electrode potentials. In SEC-NMR electrolysis operated at controlled current, working electrode potentials negative to the potential chosen for bulk electrolysis are generally accessible possibly giving rise to additional reductions of intermediates, that are not reducible in bulk conditions. As a consequence, additional reductions and intermediates, which are avoided in bulk electrolysis could generally be observed in SEC-NMR electrolysis. However, in our case, additional reduction processes cannot be observed and we thus anticipate a good transferability between SEC-NMR and bulk electrolysis.

Within these limitations, the comparability between SEC-NMR and bulk electrolysis is of qualitative rather than quantitative nature, particularly with respect to reaction kinetics.

5.2.3 Additional Information and Discussion

a) Ligand Oxidation and Gas Tightness of the SEC-NMR cell

Of note, most experiments show a minor singlet at 30 ppm in ^{31}P NMR spectra arising only after starting the electrolysis. This signal is assigned to the oxidized phosphine ligand and likely originates from the decomposition of highly reactive intermediates by trace oxygen potentially present in the NMR-electrolysis cell due to limited gas tightness.

The loss of CO_2 due to limited gas tightness was monitored by ^{13}C NMR over one hour without running an electrolysis. In this time, the $^{13}CO_2$ signal decreases by ca. 7% (Figure S 26). Comparison of this value to the decrease of the $^{13}CO_2$ integral by 30-40% during two hours of electrolysis accordingly reflects CO_2 consumption instead of major CO_2 leakage. Further, equilibration between dissolved and gaseous CO_2 was not quantitatively considered. However, as dissolved CO_2 is consumed during electrolysis, gaseous CO_2, which is not detected in initial ^{13}C SEC-NMR experiments, is expected to dissolve in the supporting electrolyte over the course of the reaction, thereby contributing to the detected $^{13}CO_2$ integral and counteracting the effect of CO_2-loss due to limited gas tightness.

b) ^{31}P NMR Signal of $[RhH(dppe)_2(CD_3CN)]^{2+}$

Measurement of the $^1J_{Rh,P}$ coupling constant for $[RhH(dppe)_2(CD_3CN)]^{2+}$ (**$Rh^{III}H$**) was rather imprecise due to a strong overlap with the doublet of the **$Rh^{I}H$** congener. Estimating more exact peak positions of the **$Rh^{III}H$** doublet from various NMR spectra indicated a $^1J_{Rh,P}$ coupling constant in the 80-100 Hz range.

c) Proton Source under Inert Conditions

From SEC-NMR experiments under inert conditions, we concluded that $n\mathrm{Bu}_4\mathrm{N}^+$ acts as proton source leading to protonation of **Rh^{-I}** into **$Rh^{I}H$**, in our conditions. Butene signals evolving after addition of $n\mathrm{Bu}_4\mathrm{NPF}_6$ to a solution of chemically reduced **Rh^{-I}** provide additional proof for Hofmann elimination of $n\mathrm{Bu}_4\mathrm{N}^+$ thereby liberating protons (Figure S 41 a-c).

Previous studies had yet suggested acetonitrile solvent as the proton source.[4-5, 8] Although we could not corroborate the formation of **Rh^{I}D** from CD_3CN by ^2H NMR spectroscopy (Figure S 12), chemical reduction experiments show the evolution of new ^1H NMR signals in the 4.5 ppm range after addition of acetonitrile to a solution containing **Rh^{-I}** and excess sodium naphtalide (Figure S 41 c-d). These signals point to reductive degradation of MeCN by reaction with either the reduced complex or sodium naphthalide (representing the electrode in electrochemical conditions). Concomitant putative proton/deuteron release from the decomposing acetonitrile solvent appears likely and we thus suspect additional protonation/deuteration of **Rh^{-I}** into **Rh^{I}H**/**Rh^{I}D** by acetonitrile. Notably, under chemical and electrochemical conditions protonated rather than (or in addition to undetected) deuterated degradation products of CD_3CN are apparent from ^1H NMR spectroscopy and likely arise from H/D-exchange within the electrolyte solution induced by the conjugated bases of MeCN and $n Bu_4N^+$ degradation products forming upon protonation of **Rh^{-I}** (Figure S 41 and S13).

The deuteration of **Rh^{-I}** in addition to the protonation of this species is further evident from integration of the ^1H hydride signal at –10.59 ppm and the CH_2 signal of the ligand backbone in **Rh^{I}H**/**Rh^{I}D** at 2.12 ppm indicating roughly a **Rh^{I}H**/**Rh^{I}D** 1:1 ratio (Figure S 44; the theoretical stoichiometry of CH_2/"H^-" is 8:1 in the absence of deuteride, while 8:0.4 was observed indicating deuteride incorporation of ca 60%). Interestingly, the protonation of **Rh^{-I}** into **Rh^{I}H**/**Rh^{I}D** is not yet at full conversion under purely chemical conditions after 24 hours, indicating enhancement for the formation of the hydride complex under our electrochemical conditions. We note that the reaction of **Rh^{-I}** with small concentrations of MeCN (1-5 eq relative to **Rh**) was reported as slow under purely chemical reaction conditions (longer than 2 hours; glassware surface protection to exclude silanols as proton source was not reported)[4]. With ca 14% **Rh^{-I}** still present in our chemical control experiment with a THF-d_8/CD_3CN (10:1) solvent mixture after 24 hours (Figure S 42 - S 43), we conclude the protonation of **Rh^{-I}** by acetonitrile to be even slower than reported, while a faster reaction rate is expected at higher MeCN concentration chosen in our SEC-NMR conditions with THF-d_8/CD_3CN (1:1). The remarkably higher protonation rate under electrochemical conditions likely results from cathodic decomposition of the electrolyte solution directly at the electrode yielding more concentrated or acidic proton source.

The formation of the corresponding deuterium hydride complex **Rh^{I}D** was also reported for labeling experiments with MeCN-d_3/nBu$_4$NClO$_4$ electrolyte.[5] Yet, Pilloni *et al.* suggested inconsistent deuterium labelling experiments as a result from fast H/D-exchange reactions upon formation of hydroxide species in trace amounts.[8] Additionally, if electrolysis is conducted in a one-chamber cell, the MeCN(-d_3)/nBu$_4$NClO$_4$ mixture gives rise to anodic evolution of (deuterated) perchloric acid that can readily act as proton (deuteron) source.[9-11] We further note that cyclic voltammetry indicates enhanced formation of **Rh^{I}H(D)** in MeCN/nBu$_4$NClO$_4$ compared to MeCN/nBu$_4$NPF$_6$, as shown from a partial loss of the reoxidation wave of **Rh^{-I}** and the build-up of the oxidation wave of **Rh^{I}H** at –1.04 V_{Fc} (Figures S34-35). Thus, the ClO_4^- anion seems to induce more reactivity under reductive conditions, which further complexify the analysis on the origin of the proton source.

Finally, the species evolving at 5.66 ppm during SEC-electrolysis under inert conditions (Figure S 13) is not apparent from control experiments in the presence of a chemical reducing agent (Figure S 41). Thus, we posit this signal to originate from anodic (follow-up) products, that are only accessible in the SEC-NMR cell. Possibly, the coordinating solvent in evolving **Rh^{III}H** that is required for coordinative saturation and stabilization can exchange for butene giving rise to olefinic ^{1}H NMR signals in this range. Alternatively, this signal might arise directly from oxidation of acetonitrile or products thereof. However, these hypotheses were neither further substantiated nor the respective species identified.

d) SEC-NMR Electrolysis of Blank Electrolyte

In SEC-NMR electrolysis in the absence of the **Rh^{I}** complex, new species in minor concentration with resonances at ca. 4.9 ppm and 8.4 ppm, which were not further assigned, likely arise from electrolyte solution decomposition (Figure S29). However, the concentration of these compounds is quite low (compare intensity scale in Figures S13 and S29) and in the range of olefinic impurities of the analyte that are already present before electrolysis (Figure S29 right).

5.3 Faradaic Efficiency

The Faradaic efficiency for the electrochemical reduction of **Rh^{I}** under inert reaction conditions was estimated from the sum of integrals for species observed in the final

SEC-^{31}P NMR spectrum according to eq. 4, where z is the number of electrons involved in the reaction (*i.e.* 2), F is the Faraday constant, n_{start} and c_{start} are the initial molar amount and concentration of **Rh^{I}**, V is the solution volume, $A_{products}$ is the sum of integrals of all P-containing complexes except for **Rh^{I}**, $A_{Rh,init}$ is the integral of **Rh^{I}** in the initial ^{31}P NMR spectrum before electrolysis and Q_{total} is the total charge consumed over electrolysis time.

$$FE_{Rh(-I)} = \frac{z \cdot F \cdot n_{start} \cdot A_{products}}{Q_{total} \cdot A_{Rh,init}} \quad \text{(eq. 3)}$$

$$= \frac{z \cdot F \cdot c_{start} \cdot \mathrm{V} \cdot A_{products}}{Q_{total} \cdot A_{Rh,init}} \quad \text{(eq. 4)}$$

For the determination of $FE_{Rh(-I)}$ is was assumed that all signals arising at chemical shifts between 43 ppm and 63 ppm originate in the two-electron reduced **Rh^{-I}** complex and all contain the identical number of P-atoms, thus, two diphosphine ligands.

6. Chemical Reduction Experiments

For NMR-scale reduction experiments, a solution of $[Rh(dppe)_2]NTf_2$ (6 µmol, 1 equiv.) in THF-d_8 (0.2 mL) was treated with an excess solution of sodium naphthalide (NaNp, 0.06 mmol, 10 equiv.) in THF-d_8 (0.3 mL). The reduction to the $Na[Rh(dppe)_2]$ congener was confirmed by ^{31}P NMR spectroscopy. Excess H_2O (0.1 mL) was added to the reduced complex upon which gas formation was observed.

Of note, the surface of the NMR tubes was silylated with Me_3SiCl prior to use to prevent adventitious protonation of the reduced complex by protic groups at the surface of the glass and increase the stability of the solution of reduced complex. For this, a Schlenk tube, that contained NMR tubes filled with Me_3SiCl, was set under light vacuum before the tube was isolated from the Schlenk line. This vessel was warmed up with a heat-gun on low power to the boiling of Me_3SiCl and let cool down to room temperature. This procedure was repeated at least three times, until all liquid was evaporated out of the NMR tubes into the surrounding Schlenk tube.

7. Density Functional Theory Methods

Gaussian 16, Revision B.01[12] was used for density functional theory (DFT) computations. All computations were performed in the unrestricted mode. Spin contamination was excluded by $S2$ values of 0 for the singlet spin state, of 0.75 ± 4.5% for the doublet spin state and of 2 ± 1% for the triplet spin state. Preoptimization of all structures was performed in the gas phase with the MN12L density functional[13] on a split valence basis with polarization functions (def2-SVP).[14-16] The preoptimized structures were refined in acetonitrile (smd-model,[17] p = 468 atm[18] on a triple-zeta basis set with polarization functions and effective core potentials for transition metals (def2-TZVP(ECP))[14, 19] using the density functional MN12L.[13] Subsequent frequency calculations on the optimized structures were performed to confirm these structures as minima by the absence of imaginary frequencies. Reduction potentials were computed from the free energies ΔG (def2-TZVP in acetonitrile) for the formal reaction of the respectively oxidized [Rh]-species ($[Rh]^q$) with ferrocene (Fc) as reference yielding the reduced [Rh]-congener ($[Rh]^{q-1}$) and the ferrocenium cation (Fc^+) in acetonitrile:

$$[Rh]^q + Fc \rightarrow [Rh]^{q-1} + Fc^+$$

$$\Delta G = G(Fc^+) + G([Rh]^{q-1}) - G(Fc) - G([Rh]^q)$$

$$E^0(Rh^{q/q-1}) = \Delta G \cdot n^{-1} \cdot F^{-1}$$

The choice of the MN12L functional and the def2-TZVP basis set is based on previously observed satisfactory correlations of computational energy span values for late transition metal complexes in chemocatalysis[20] and of computational reduction potentials of rhodium phosphine complexes[21] with corresponding experimental data.

8. Electrochemical Simulations with *DigiElch*

Simulations of CVs and chronoamperometry (CA) concentration profiles were carried out with *DigiElch Professional 8.0*. Settings are exemplarily shown in Figure S 3- S6.

A planar electrode geometry (A_{CV} = 0.07 cm^2) and semi-infinite one-dimensional diffusion was assumed for CV simulations. CA simulations were operated at a band electrode (A_{CA} = 1 cm^2) and semi-infinite two-dimensional diffusion. The smart pre-equilibrium option was enabled for all simulations to compute analytical concentrations of all species with respect to the applied potential. Room temperature (298.2 K) was set for all simulations.

CVs were simulated at a scan rate of 100 mV·s^{-1} and CAs at an applied potential of –2.5 V_{Fc} operating in stationary state. Resulting current densities in simulated CAs were in the range of 50 µA·cm^{-2}.

The diffusion coefficients for all Rh containing species were set to 5.3·10^{-6} cm^2·s^{-1}, as experimentally derived for **Rh^{I}**. For protons (in the form of water) and hydrogen, diffusion coefficients of 1·10^{-5} cm^2·s^{-1} were assumed.[22-25]

As charge-transfer reactions, the redox couples for **Rh$^{I/0}$** and **Rh$^{0/-I}$** were approximated both at a potential of E^0 = –2.12 V_{Fc} with an electron transfer rate k_{ET} = 0.0053 cm·s^{-1} and transfer coefficient α = 0.5, based on previous work.[1] The redox couple for **Rh$^{III/I}$H** was assumed at a potential of E^0 = –1.07 V_{Fc} and the same k_{ET} and α were used (k_{ET} = 0.0053 cm·s^{-1}; α = 0.5). The Marcus-Hush model was used to describe electron transfer kinetics.

In CV, respectively CA, simulations, initial concentrations were set to 1 – 1.6 mM, resp. 8.5 mM for **Rh^{I}** and 1 M, resp. 2 M for protons for comparison to experiments in the presence of water, corresponding to experimental conditions. These concentrations were kept constant at the right-hand boundary in bulk (corresponding to infinite distance from electrode surface).

Aside the protonation of **Rh^{-I}** yielding **Rh^{I}H** at an experimental rate constant of k_f = 2.3 s^{-1} under argon (see sections 4 and 10.1 in SI), hydrogen formation from the reaction of **Rh^{I}H** with a proton was simulated at different rate constants (k_{HER}),

assuming an equilibrium strongly on the product side ($K_{eq} = 1 \cdot 10^6$).[26-27] Further chemical reactions were not considered.

Fitting the simulated anodic current of the oxidation wave of **Rh^{I}H** to the experimentally observed one allows to deliver a rough estimate of k_{HER}.

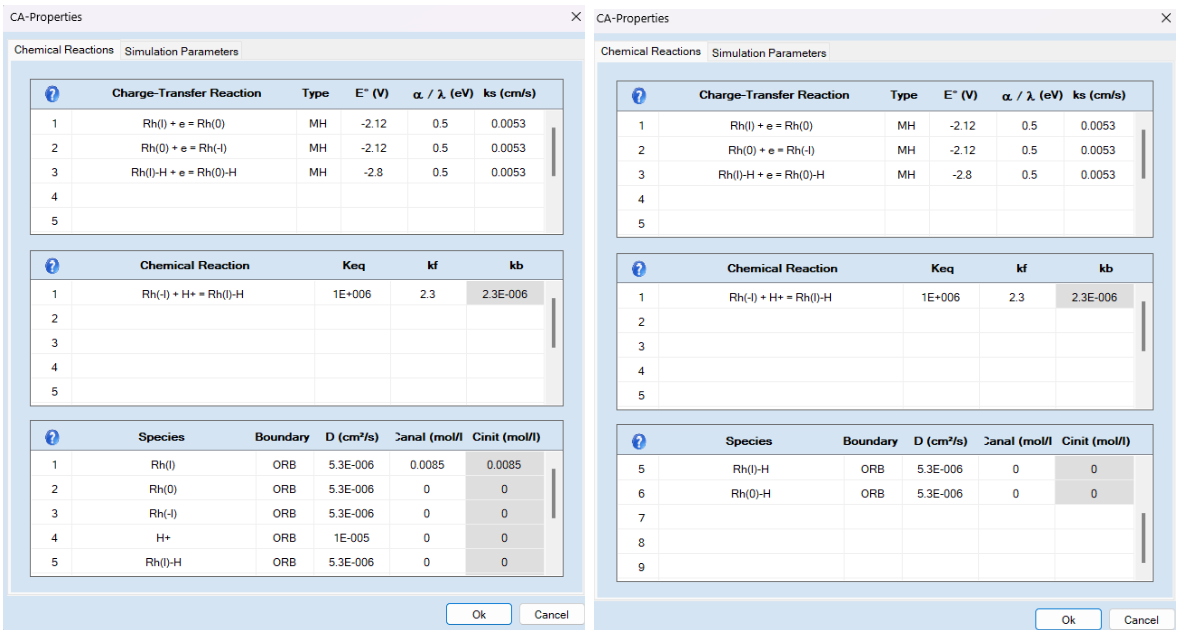

Figure S 3. Screenshot of “Chemical Reactions” Settings in DigiElch for chronoamperometric simulations.

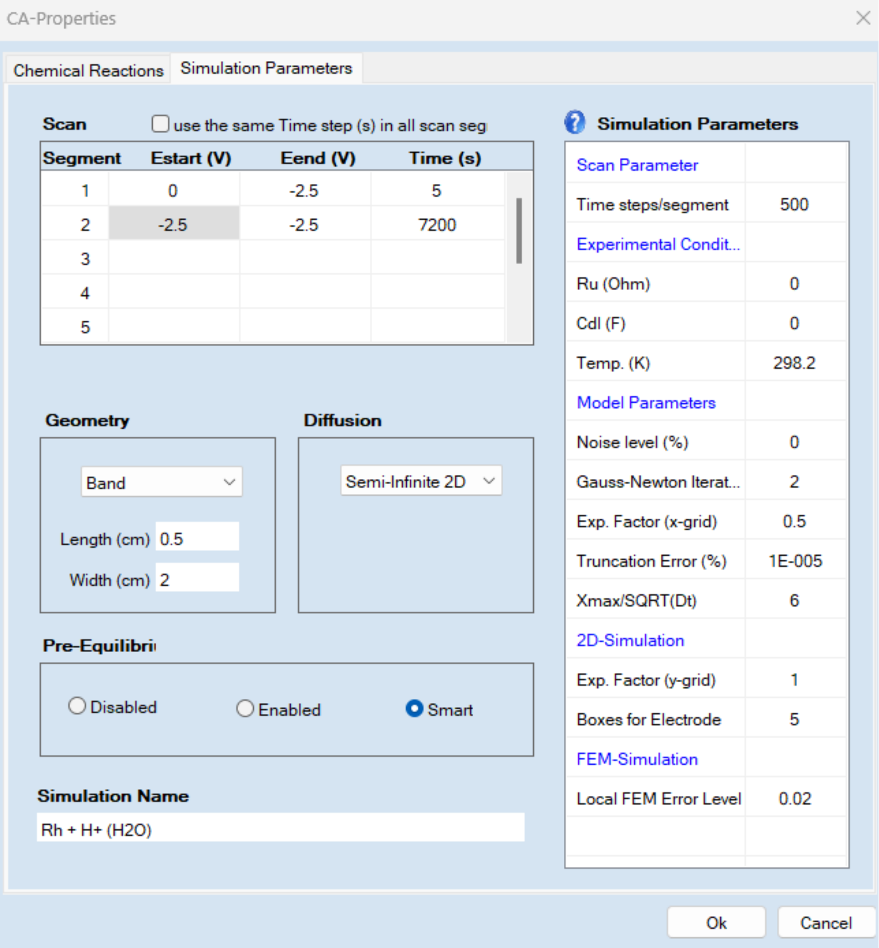

Figure S 4. Screenshot of “Simulation Parameters” Settings in *DigiElch* for chronoamperometric simulations.

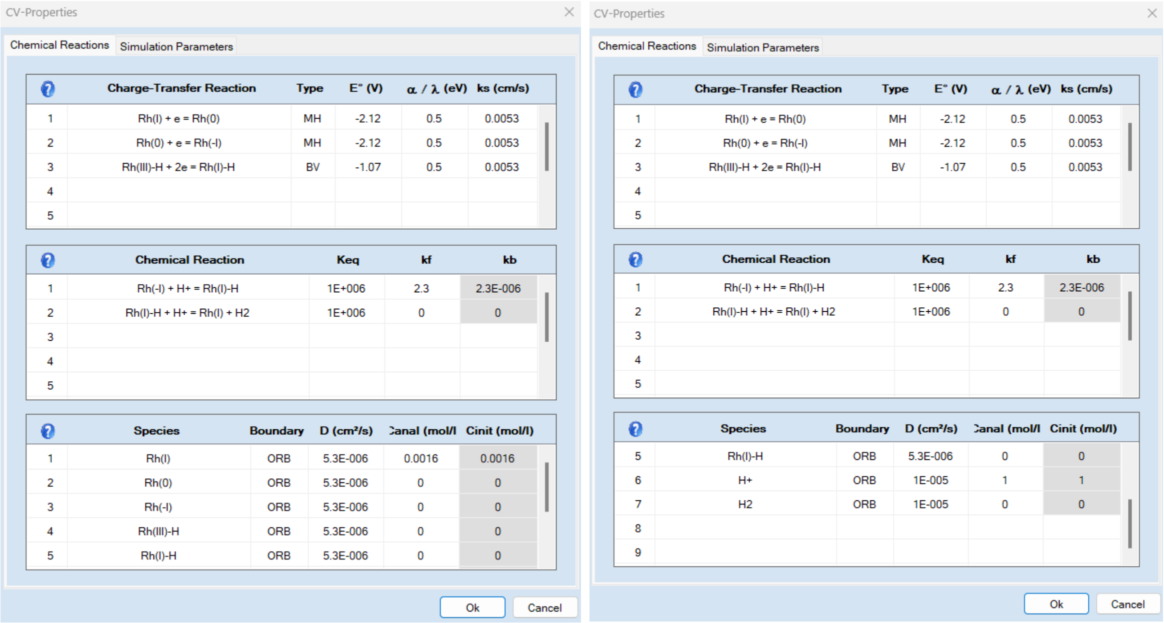

Figure S 5. Screenshot of “Chemical Reactions” Settings in *DigiElch* for simulations of cyclic voltammograms.

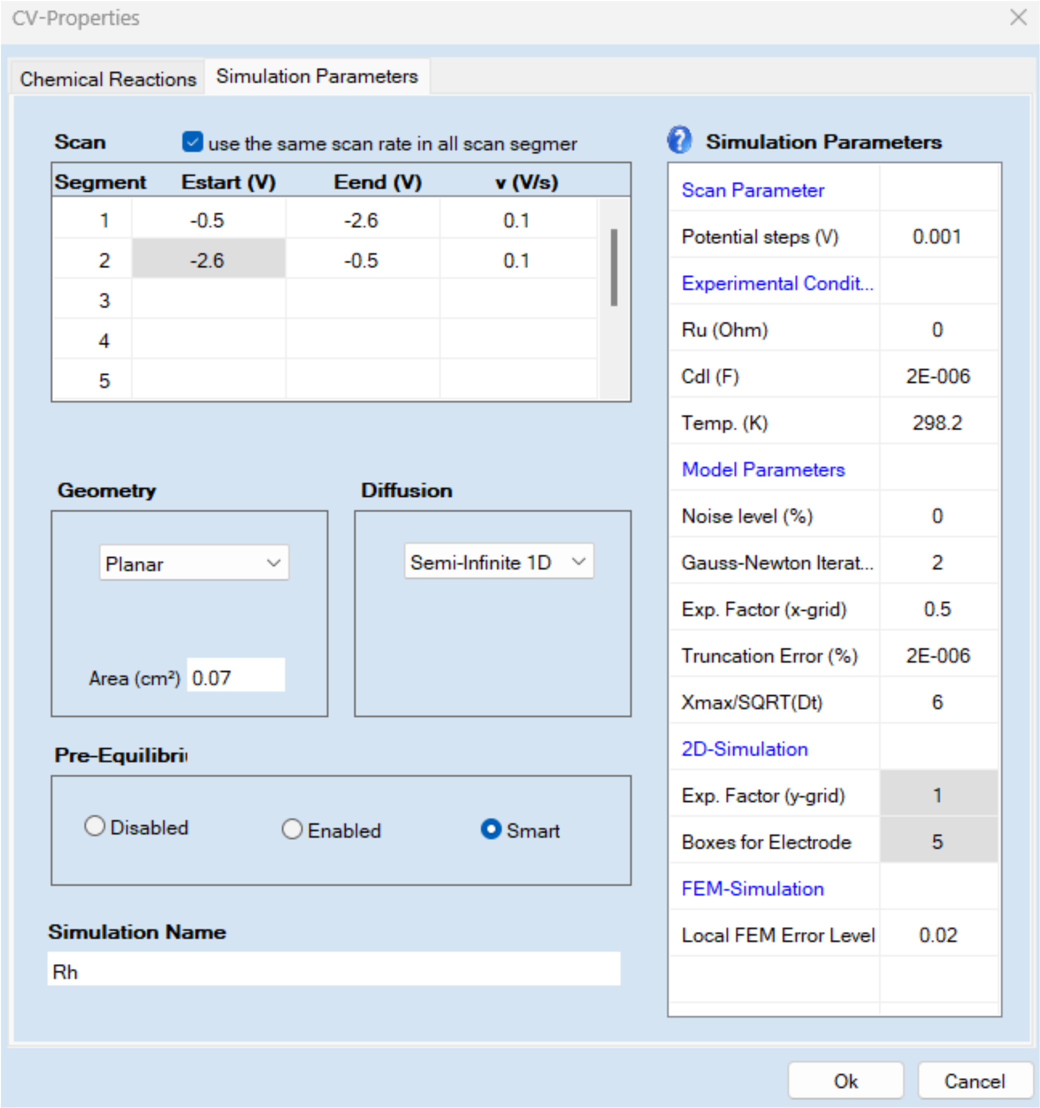

Figure S 6. Screenshot of “Simulation Parameters” Settings in *DigiElch* for simulations of cyclic voltammograms.

9. Supporting Figures: Spectroelectrochemistry

9.1 Potential Profiles for Controlled Current Electrolysis

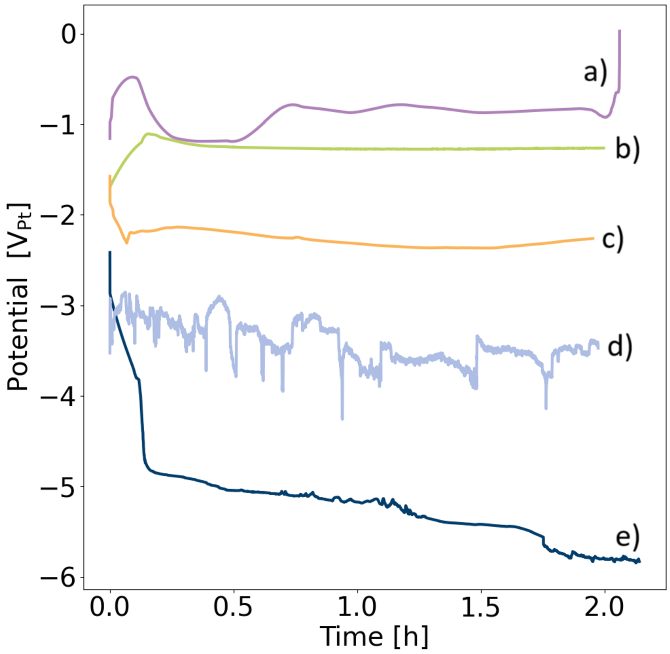

Figure S 7. Potential-time curves pseudoreferenced against Pt for controlled current electrolysis at –0.4 mA. Conditions: a) [Rh(dppe)$_2$]NTf$_2$, Ar, MeCN-*d*$_3$/THF-*d*$_8$. b) [Rh(dppe)$_2$]NTf$_2$, Ar, H_2O (2 M), THF-*d*$_8$. c) no [Rh(dppe)$_2$]NTf$_2$, Ar, MeCN. d) [Rh(dppe)$_2$]NTf$_2$, CO_2, H_2O (2 M), THF-*d*$_8$. e) [Rh(dppe)$_2$]NTf$_2$, CO_2, MeCN. Notes: The potential of e) strongly deviates from a)-d) due to a deficient contact in the used set-up bringing about additional resistance in the system.

9.2 Electrolysis of $[Rh(dppe)_2]^+$ under Inert Reaction Conditions

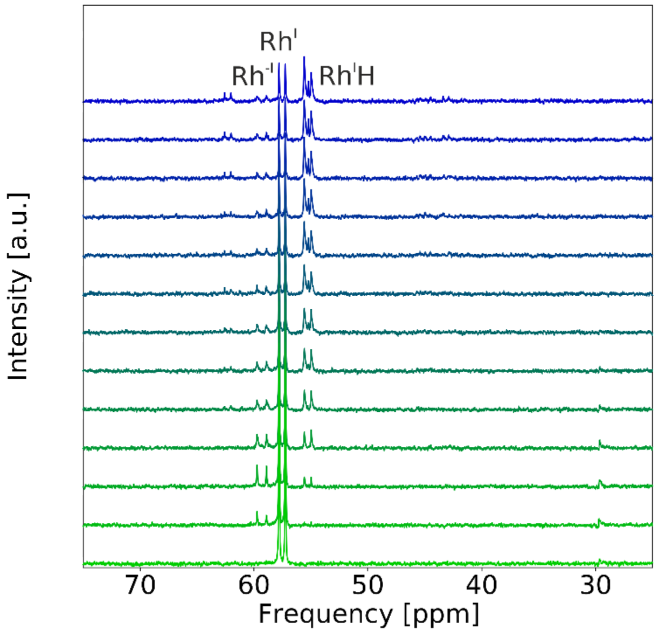

Figure S 8. SEC-^{31}P NMR measurements during an electrolysis of $[Rh(dppe)_2]NTf_2$ at –0.4 mA in MeCN-d_3/THF-d_8 under Ar atmosphere. The gradual colour change of the NMR spectra refers to measurements at proceeding time from t = 0 (green) to 120 (blue) min.

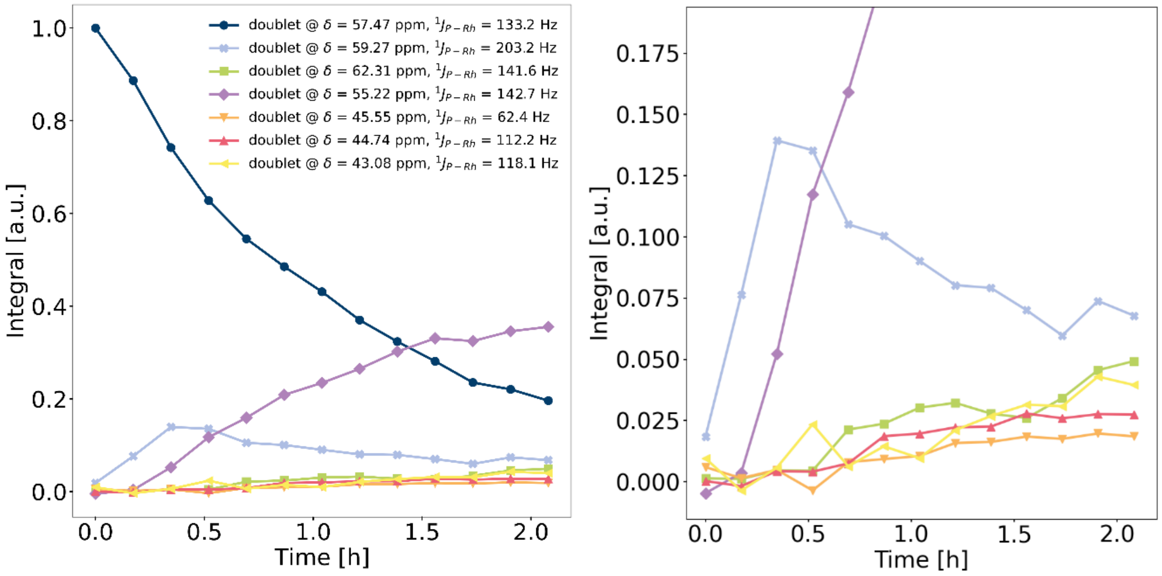

Figure S 9. Change of integrals of SEC-^{31}P NMR signals in Figure S8. Left: full axis. Right: zoom-in. Dots are data points, while lines are added as optical guide of the trend.

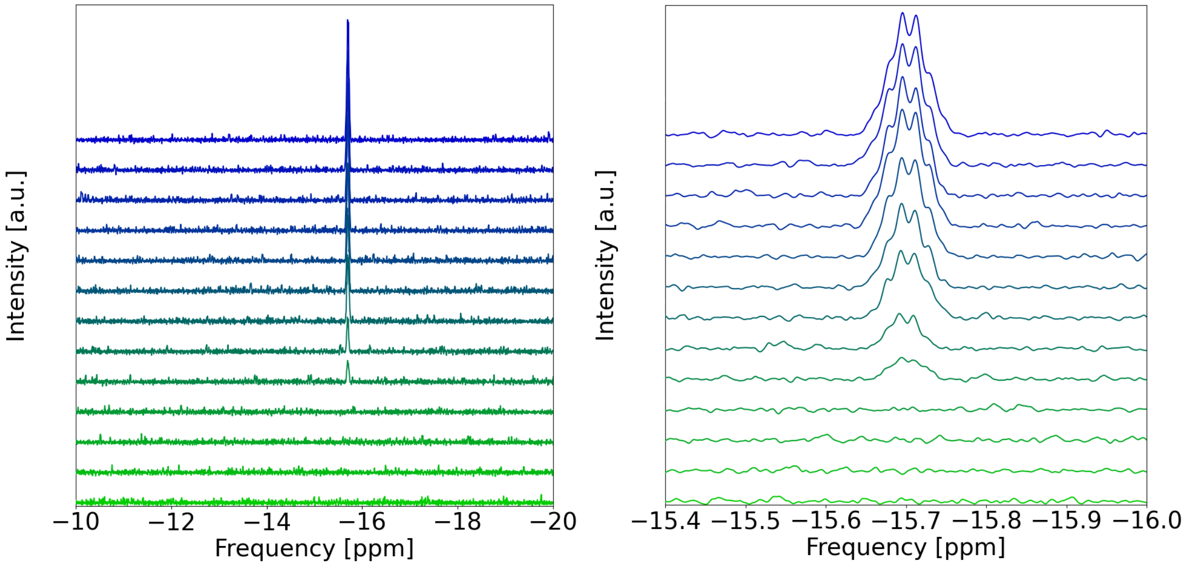

Figure S 10. SEC-^{1}H NMR measurements in the hydride region during constant current electrolysis of $[Rh(dppe)_2]NTf_2$ at –0.4 mA in MeCN-d_3/THF-d_8 under Ar atmosphere. Left: full spectrum. Right: zoom-in. The gradual colour change of the NMR spectra refers to measurements at proceeding time from t = 0 (green) to 120 (blue) min.

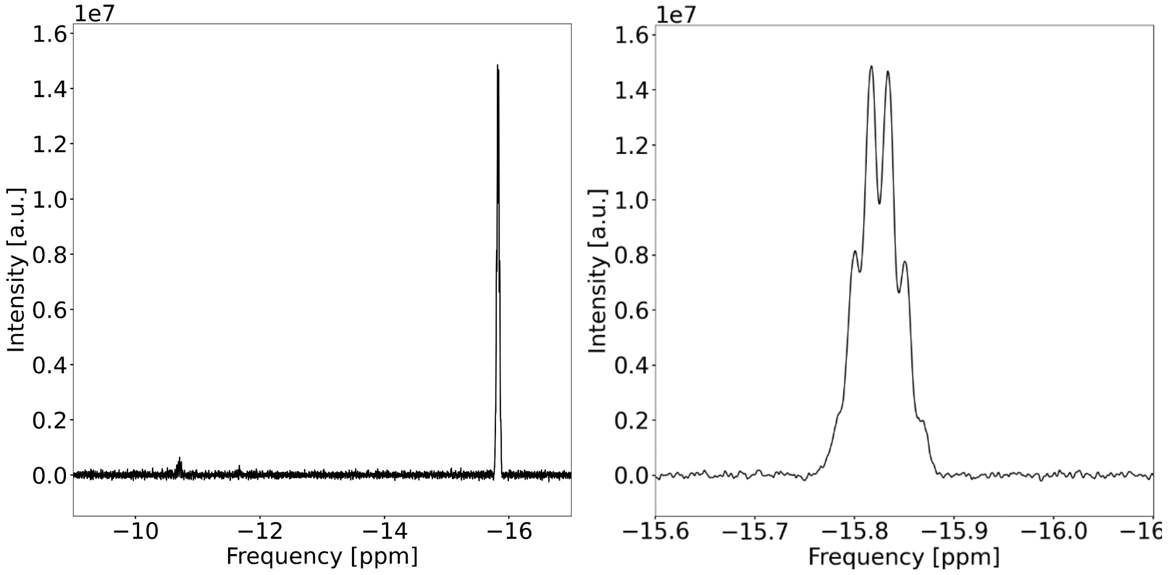

Figure S 11. Ex situ measured ^{1}H NMR spectrum of the hydride region after an electrolysis of $[Rh(dppe)_2]NTf_2$ at –0.4 mA in MeCN-d_3/THF-d_8 under Ar atmosphere. Left: full spectrum. Right: zoom-in.

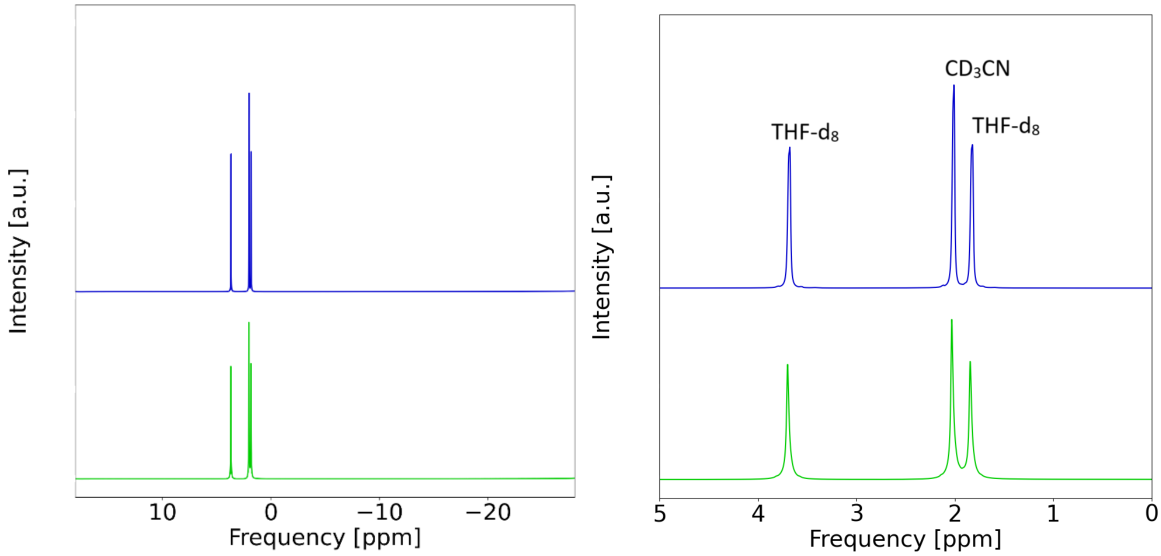

Figure S 12. Ex situ recorded ^{2}H NMR spectrum after electrolysis of $[Rh(dppe)_2]NTf_2$ at –0.4 mA in MeCN-d_3/THF-d_8 under Ar atmosphere. Left: full spectrum. Right: zoom-in. The bottom green spectrum was recorded before electrolysis was started, the blue top spectrum was recorded after electrolysis was finished.

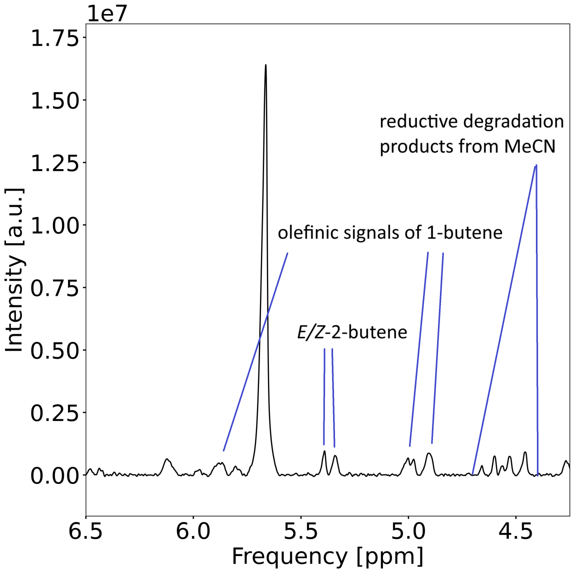

Figure S 13. Ex situ measured ^{1}H NMR spectrum of the olefinic region after an electrolysis of $[Rh(dppe)_2]NTf_2$ at –0.4 mA in MeCN-d_3/THF-d_8 under Ar atmosphere.

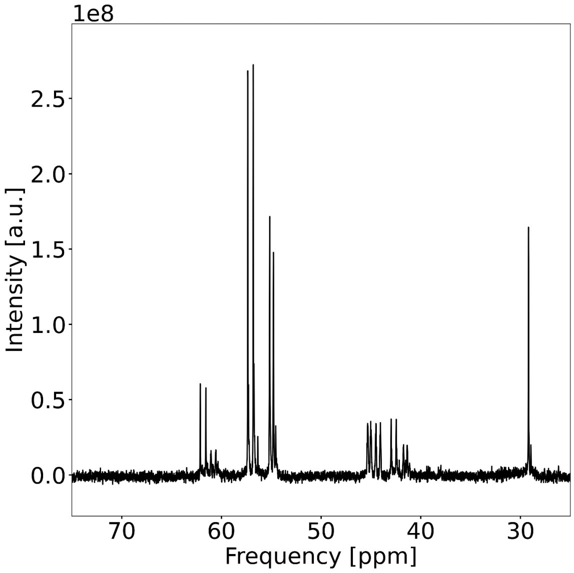

Figure S 14. Ex situ measured ^{31}P NMR spectrum after electrolysis of $[Rh(dppe)_2]NTf_2$ at –0.4 mA in MeCN-d_3/THF-d_8 under Ar atmosphere.

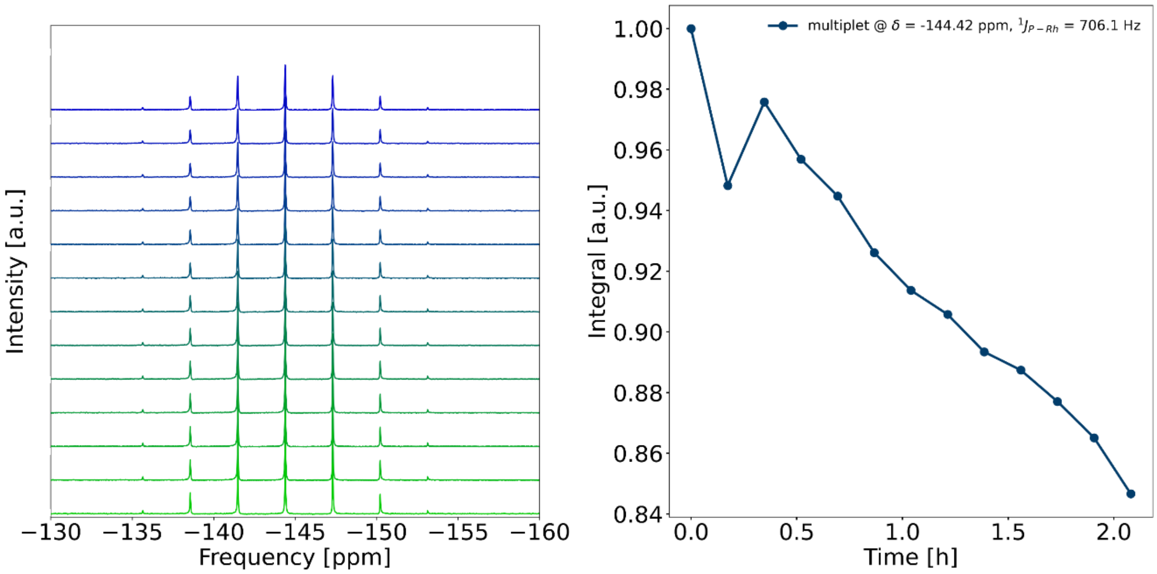

Figure S 15. SEC-^{31}P NMR signal for the PF_6^- region (144.4 ppm) during constant current electrolysis of $[Rh(dppe)_2]NTf_2$ at –0.4 mA in MeCN-d_3/THF-d_8 under Ar atmosphere (left) and corresponding integral of this signal (right). Dots are data points, while lines are added as optical guide of the trend. The gradual colour change of the NMR spectra refers to measurements at proceeding time from t = 0 (green) to 120 (blue) min. The ^{31}P integral of the PF_6^- signal measured in the second NMR spectrum is considered an outlier.

9.3 Electrolysis of $[Rh(dppe)_2]^+$ under Protic Conditions in Argon Atmosphere

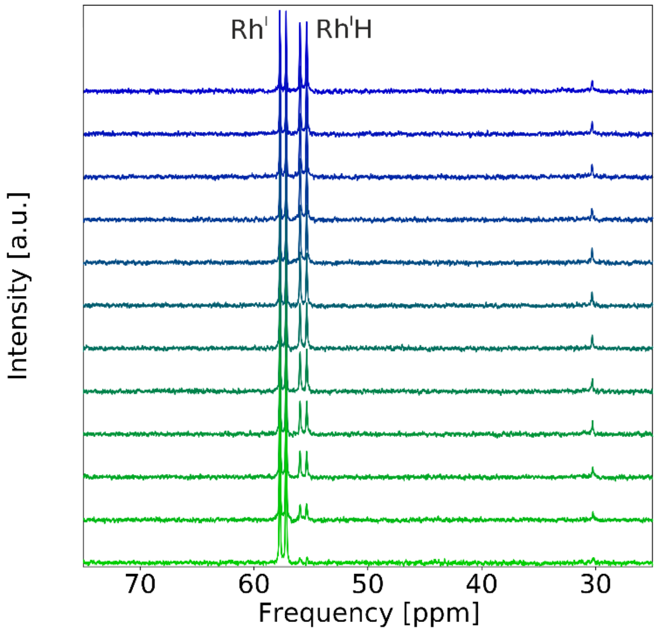

Figure S 16. SEC-^{31}P NMR measurements during an electrolysis of $[Rh(dppe)_2]NTf_2$ at –0.4 mA in THF-d_8 with H_2O (2 M) under Ar atmosphere. The gradual colour change of the NMR spectra refers to measurements at proceeding time from t = 0 (green) to 120 (blue) min.

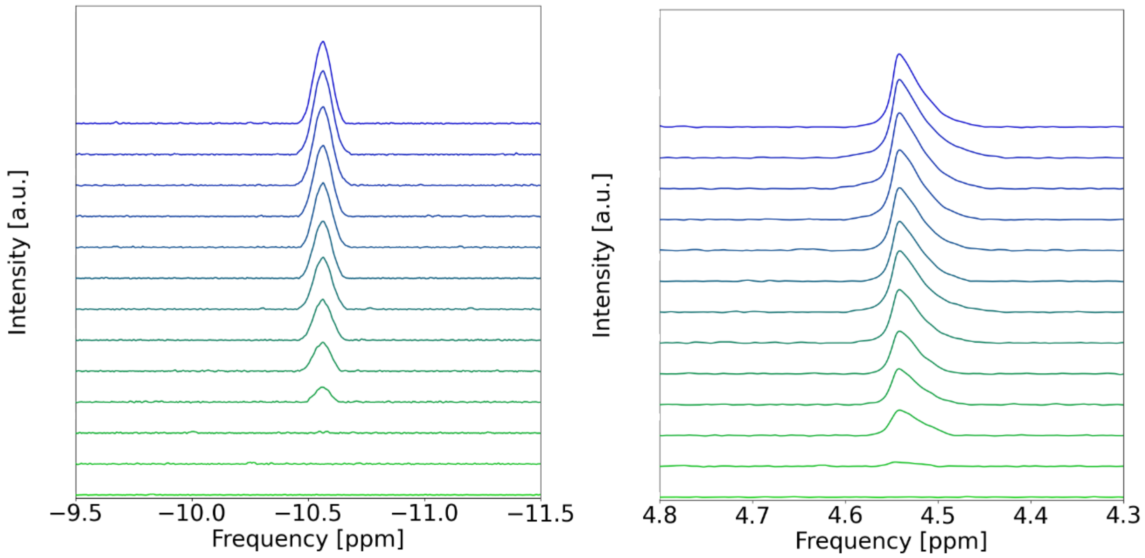

Figure S 17. SEC-^{1}H NMR spectra recorded during electrolysis of $[Rh(dppe)_2]NTf_2$ at –0.4 mA in THF-d_8 with H_2O (2.1 M) under Ar atmosphere. Left: hydride region. Right: H_2 region (4.6 ppm). The gradual colour change of the NMR spectra refers to measurements at proceeding time from t = 0 (green) to 120 (blue) min. The asymmetry of the H_2 signal might originate from a concentration gradient of dissolved H_2 towards the working electrode within the sensitive volume or from exchange reactions and was not further analyzed.

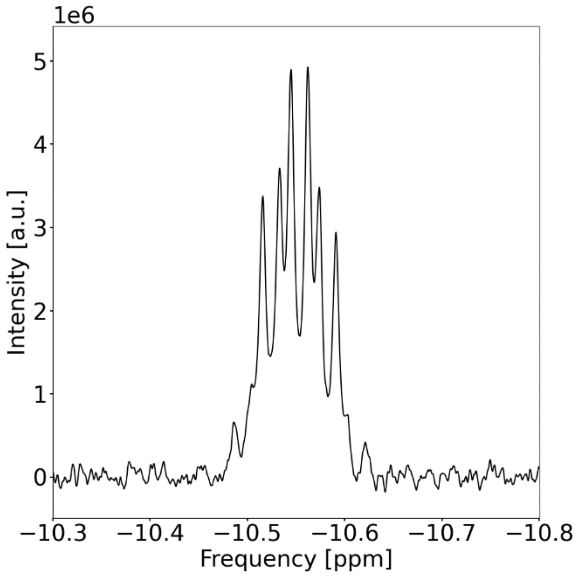

Figure S 18. Ex situ measured ^{1}H NMR spectrum after electrolysis of $[Rh(dppe)_2]NTf_2$ electrolysis of $[Rh(dppe)_2]NTf_2$ at –0.4 mA in THF-d_8 with H_2O (2.1 M) under Ar atmosphere. The $\mathbf{Rh^{I}H}$ signal is observed. The multiplicity of a pentet of doublets with coupling constants of $^{1}J_{P,H}$ = 17.6 Hz and $^{1}J_{Rh,H}$ = 10.3 Hz is in excellent agreement with reported data.[4]

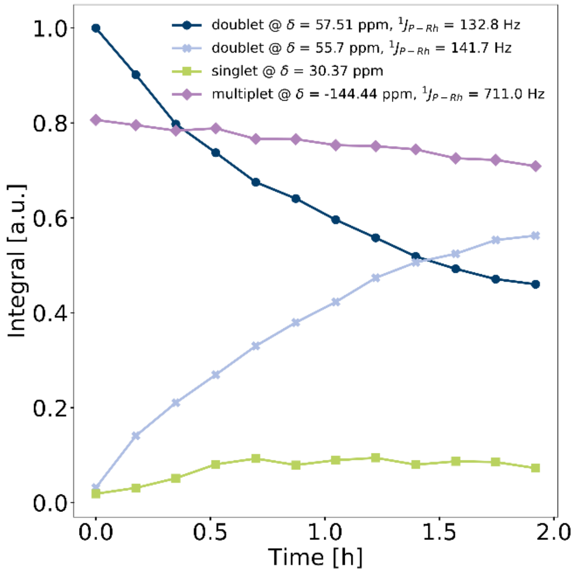

Figure S 19. Integrals of ^{31}P NMR signals in Figure S16. Dots are data points, while lines are added as optical guide of the trend.

9.4 Electrolysis of $[Rh(dppe)_2]^+$ under CO_2 Atmosphere

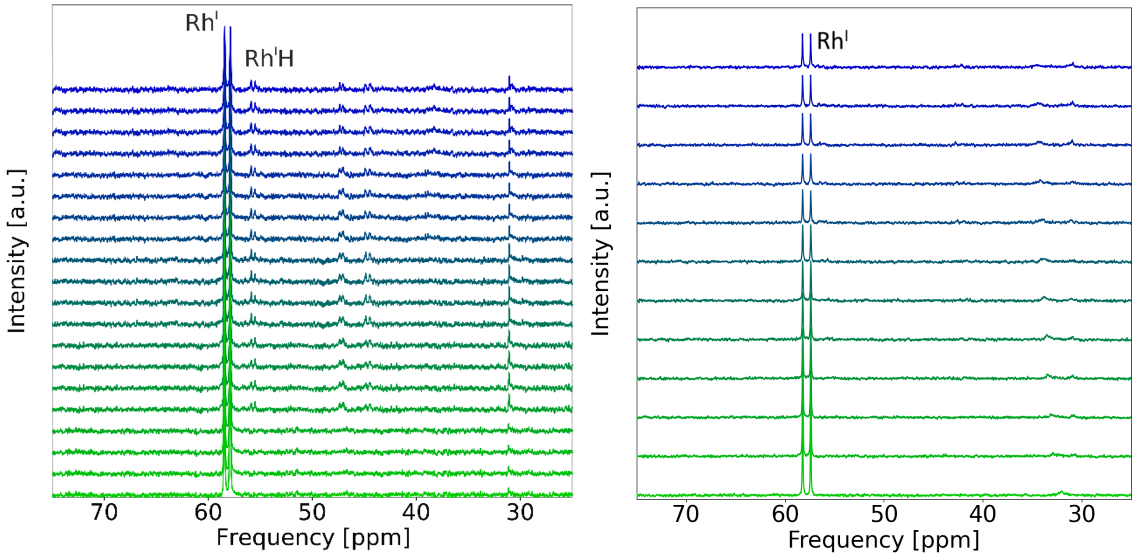

Figure S 20. SEC-^{31}P NMR measurements during electrolysis of $[Rh(dppe)_2]NTf_2$ at –0.4 mA. Left: CO_2, CH_3CN. Right: CO_2, H_2O (2 M), THF-d_8; experiment was performed on a 400 MHz device. The gradual colour change of the NMR spectra refers to measurements at proceeding time from t = 0 (green) to 120 (blue) min.

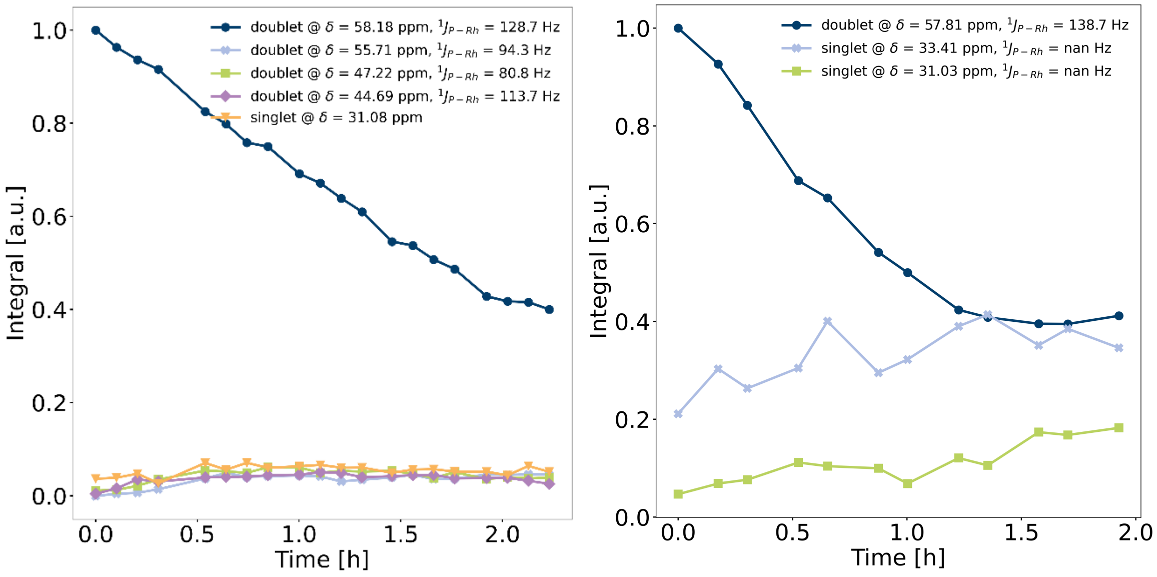

Figure S 21. Integrals of ^{31}P NMR signals in Figure S20. Left: CO_2, CH_3CN. Right: CO_2, H_2O (2 M), THF-d_8; experiment was performed on a 400 MHz device. Dots are data points, while lines are added as optical guide of the trend.

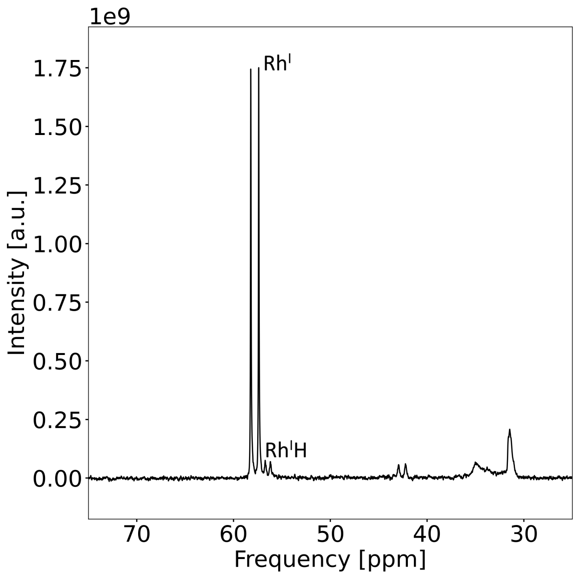

Figure S 22. Ex situ measured ^{31}P NMR spectrum after electrolysis of [Rh(dppe)$_2$]NTf$_2$ at –0.4 mA in THF-d_8 under CO_2 atmosphere with H_2O (2 M). The experiment was performed on a 400 MHz device.

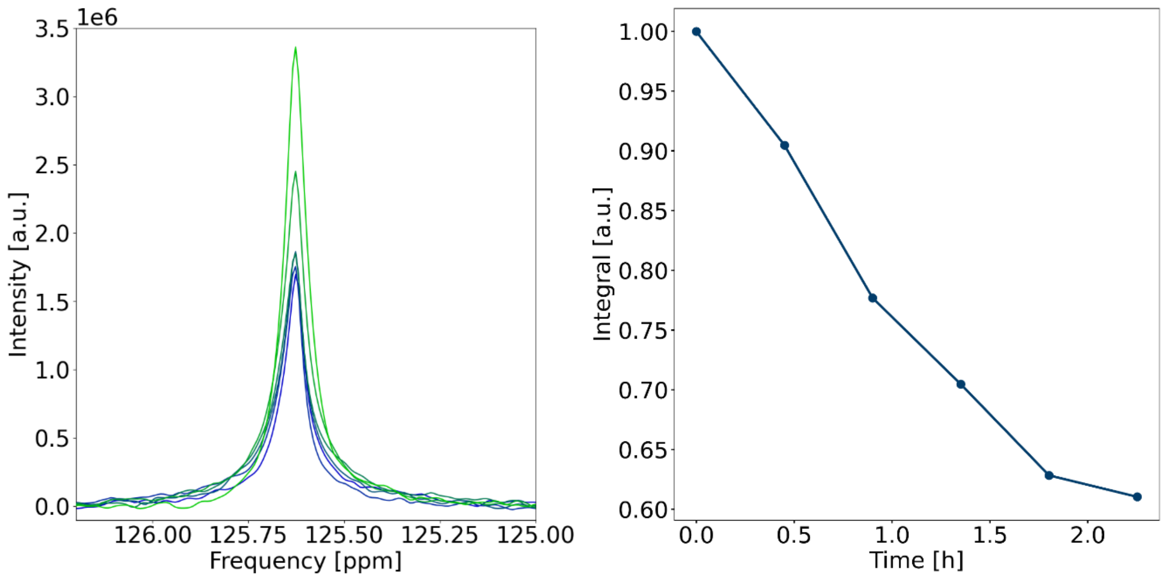

Figure S 23. SEC-^{13}C NMR signals (left) of CO_2 and corresponding integrals (right) indicating CO_2 consumption during electrolysis of [Rh(dppe)$_2$]NTf$_2$ at –0.4 mA in CH_3CN under CO_2 atmosphere. The gradual colour change of the NMR spectra refers to measurements at proceeding time from t = 0 (green) to 120 (blue) min. Dots are data points, while lines are added as optical guide of the trend.

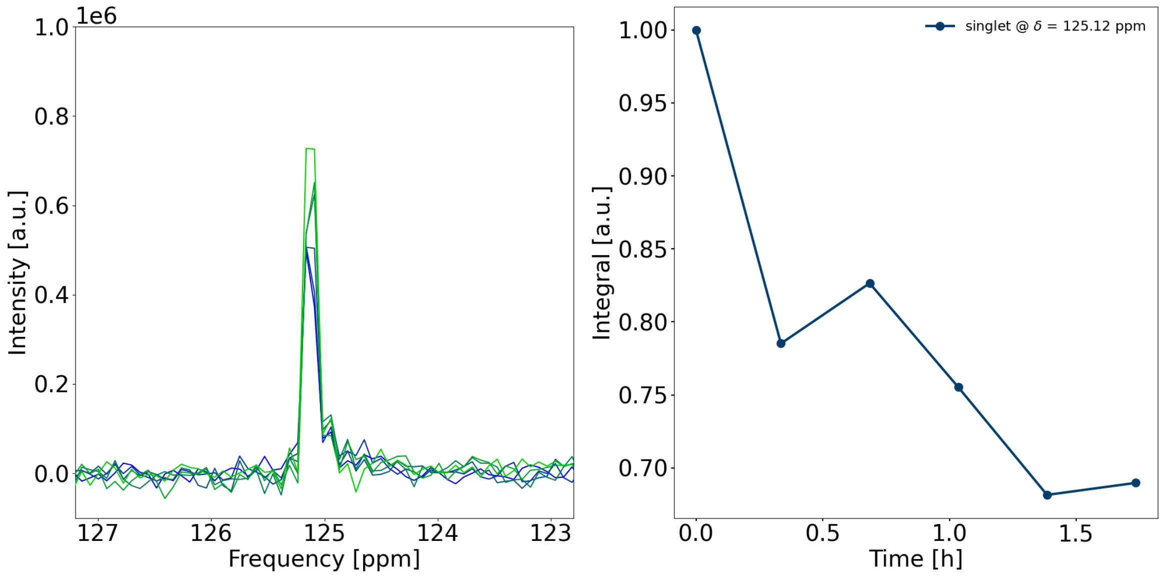

Figure S 24. SEC-^{13}C NMR signals (left) of CO_2 (125 ppm) and corresponding integrals (right) during electrolysis of $[Rh(dppe)_2]NTf_2$ at –0.4 mA in THF-d_8 with H_2O (2 M) under CO_2 atmosphere. The experiment was performed on a 400 MHz device. The gradual colour change of the NMR spectra refers to measurements at proceeding time from *t* = 0 (green) to 120 (blue) min. Dots are data points, while lines are added as optical guide of the trend. The ^{13}C integral of CO_2 signal measured in the second NMR spectrum is considered an outlier.

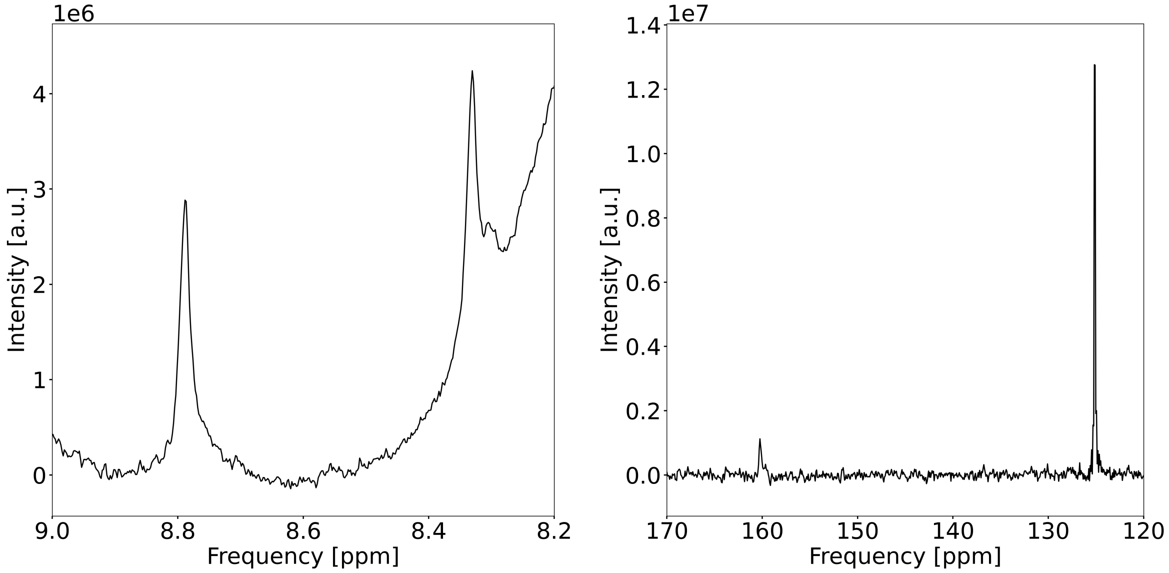

Figure S 25. Ex situ measured ^{1}H (left) and ^{13}C (right) NMR spectra of the formate regions after an electrolysis of $[Rh(dppe)_2]NTf_2$ at –0.4 mA in THF-d_8 with H_2O (2 M) under CO_2 showing a doublet for $^{1}H\text{-}^{13}CO_2^{-}$ in the ^{1}H NMR centered at 8.53 ppm ($^{1}J_{H,C}$ = 183 Hz) and the ^{13}C signal at 159 ppm. Of note, the trapezoidal-like geometry of the ^{1}H-doublet results from an overlapping signal.

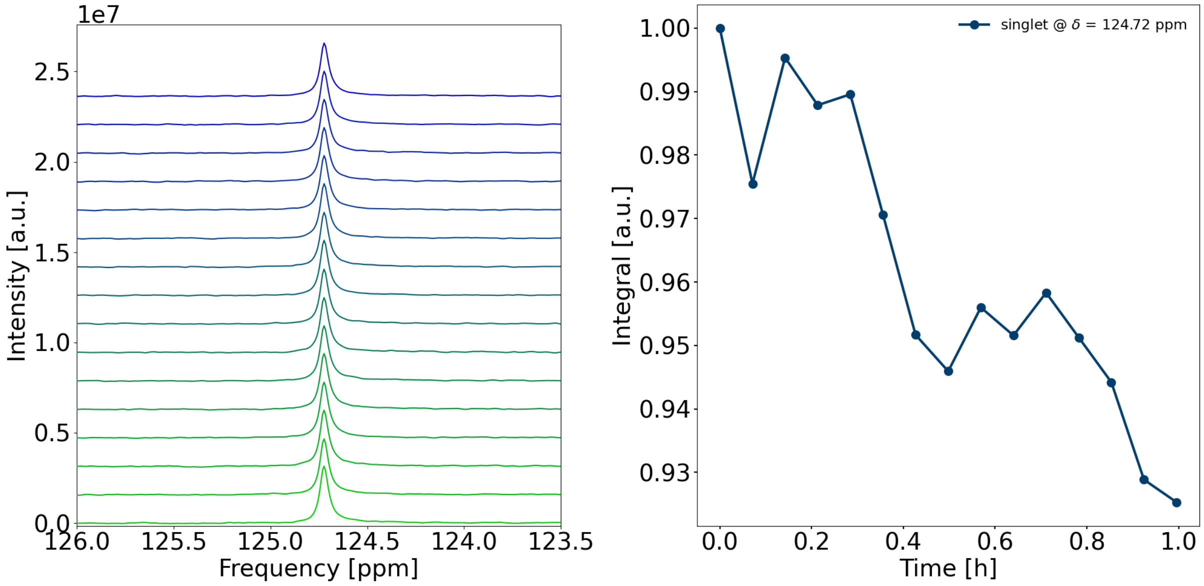

Figure S 26. ^{13}C NMR signal of $^{13}CO_2$ (left) and the corresponding integral (right) during an OCV measurement over one hour showing ca. 7% loss of CO_2 due to limited gas tightness of the cell. Conditions: $[Rh(dppe)_2]NTf_2$ in THF with D_2O (2 M) under CO_2 atmosphere.

9.5 Electrolysis of Neat Electrolyte

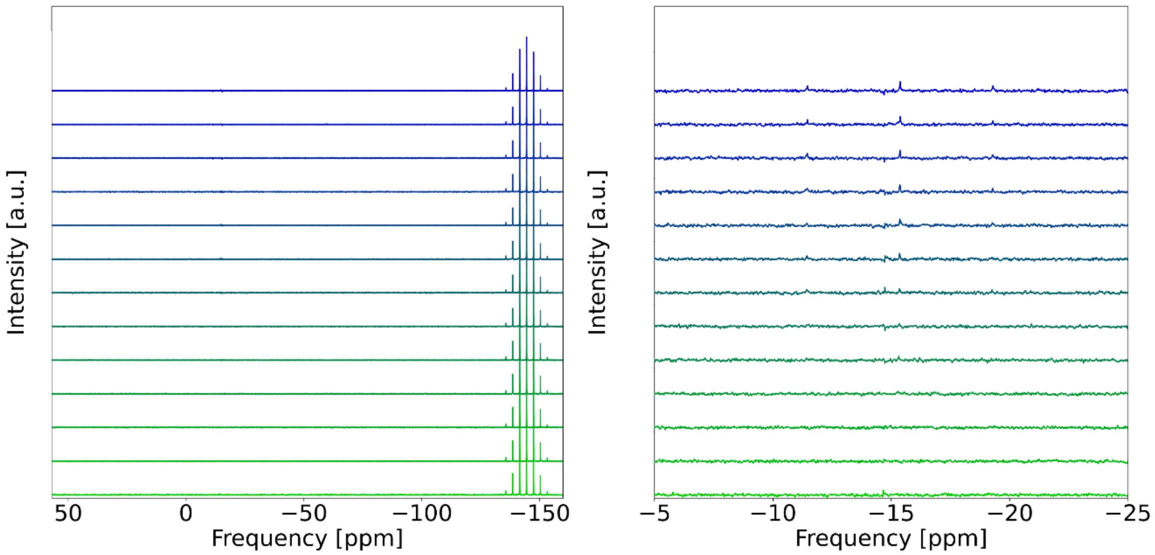

Figure S 27. SEC-^{31}P NMR measurements during electrolysis at –0.4 mA of neat THF electrolyte under Ar atmosphere. Left: full spectra. Right: Zoom-in. The gradual colour change of the NMR spectra refers to measurements at proceeding time from *t* = 0 (green) to 120 (blue) min.

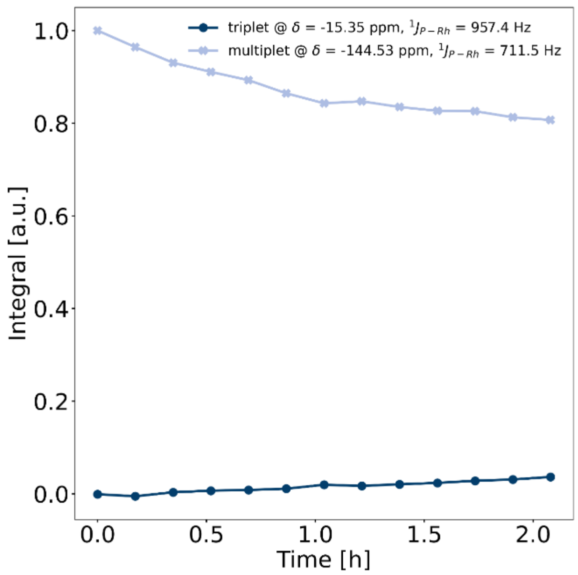

Figure S 28. Integrals of ^{31}P NMR signals in Figure S27. Dots are data points, while lines are added as optical guide of the trend.

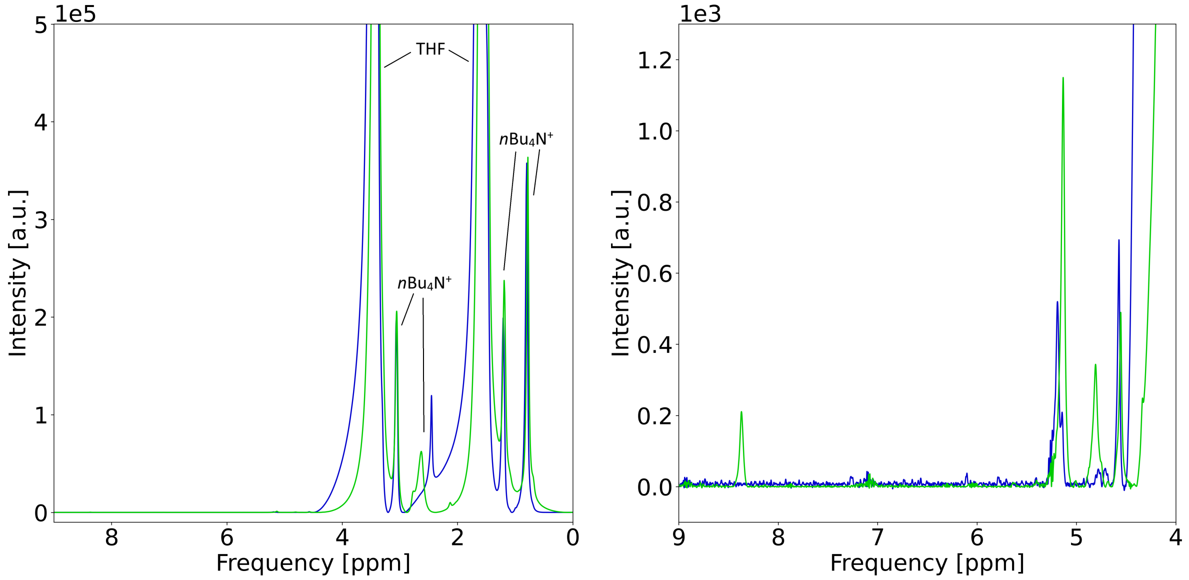

Figure S 29. Ex situ measured ^{1}H NMR spectra of the olefinic region before (blue) and after (green) an electrolysis of neat electrolyte *n*Bu_4NPF_6/THF at –0.4 mA under Ar atmosphere. Left: full spectrum. Right: intensity and chemical shifts zoomed-in.

9.6 Spectroelectrochemical UV/Vis

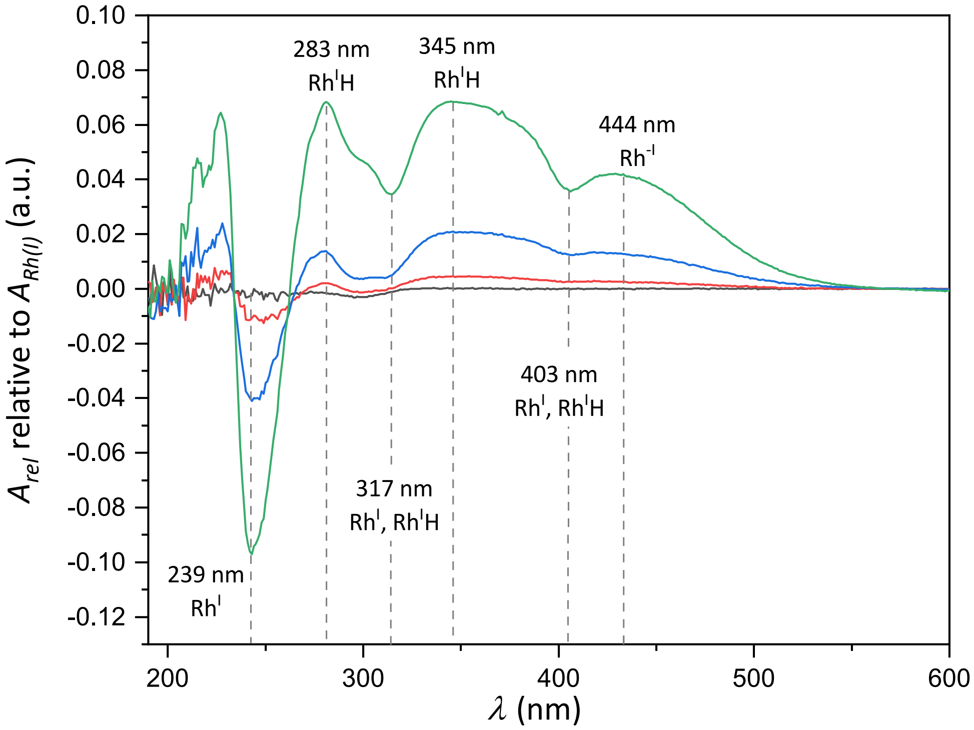

Figure S 30. SEC-UV/Vis differential spectra during electrolysis of a solution of $[Rh(dppe)_2]NTf_2$ (0.25 mM) at E_{app} = –2.28 V_{Fc}. Black: pristine solution before start, red: after 1s, blue: after 5 s, green: after 30 s.

Table S 1. UV/Vis absorbance maxima of the rhodium complexes in isolated form and under spectro-electrochemical conditions. Measured in [a)]MeCN, [b)]benzene/toluene crude reaction mixture, [c)]THF; the use of different solvents is required due to the solubilities of the different compounds.[4]

Complex	λ (nm)	
	Isolated	SEC-UV/Vis[a)]
$[Rh^{I}(dppe)_2]^+$	238, 316, 405[a)]	239, 317, 403
$[Rh^{0}(dppe)_2]$	615[b)4, 28]	-
$[Rh^{-I}(dppe)_2]^-$	425, 462[c)4]	444
$[Rh^{I}H(dppe)_2]$	294, 314, 346, 407[c)]	283, 317, 345, 403

10. Supporting Figures: Cyclic Voltammograms and Kinetic Plots

10.1 [Rh(dppe)$_2$]NTf$_2$ with H_2O (1 M) under Ar

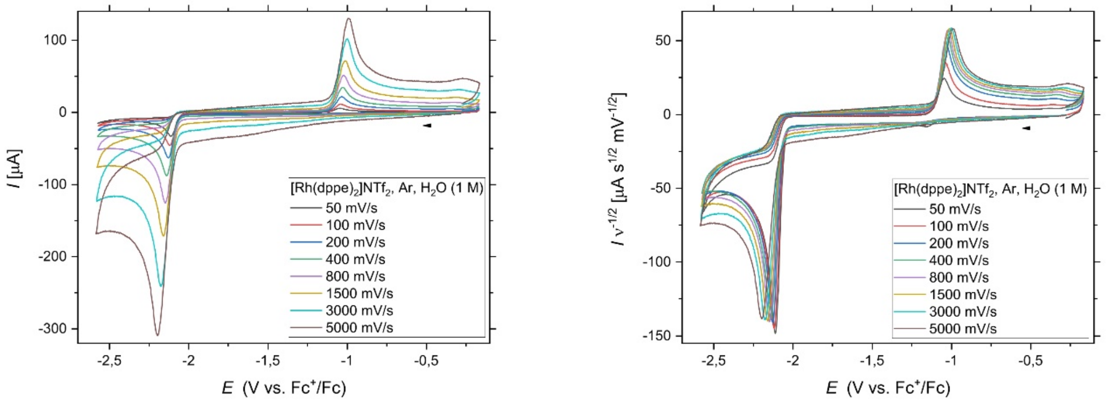

Figure S 31. Cyclic voltammograms of [Rh(dppe)$_2$]NTf$_2$ at varied scan rates. Conditions: Ar, H_2O (1 M).

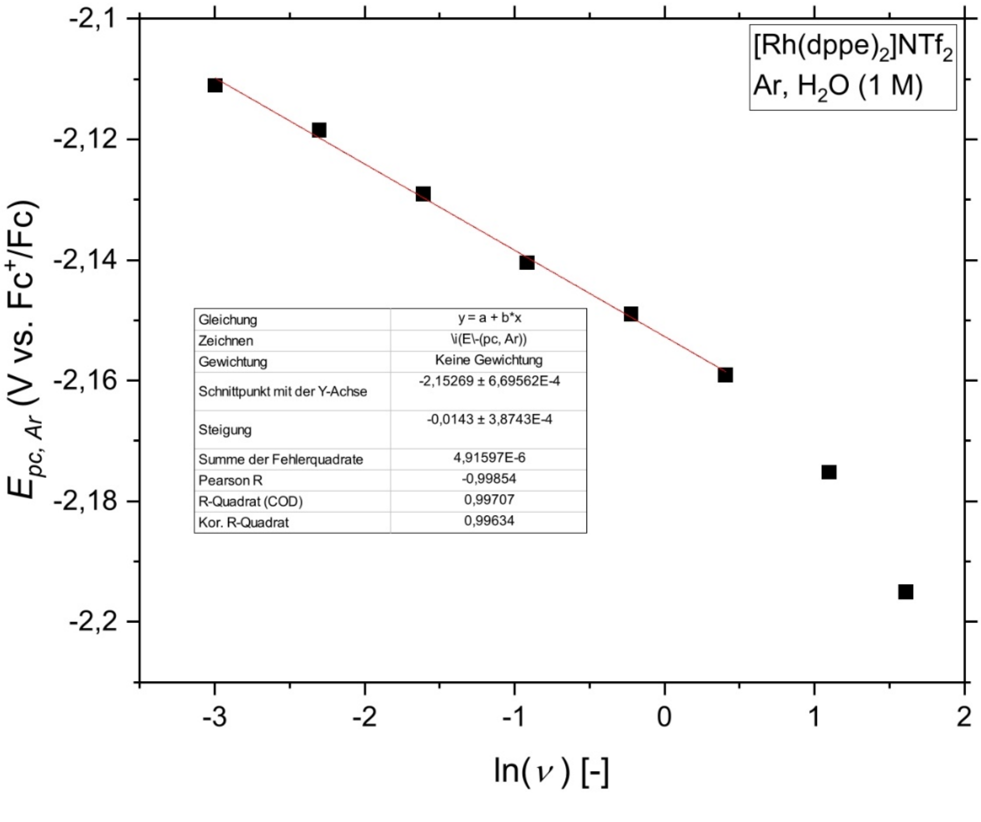

Figure S 32. Linear regression of cathodic peak potential variation in scanrate dependent experiments for the determination of the chemical reaction rate constant. Conditions: Ar, H_2O (1 M).

10.2 [Rh(dppe)$_2$]NTf$_2$ with H_2O (1 M) under CO_2

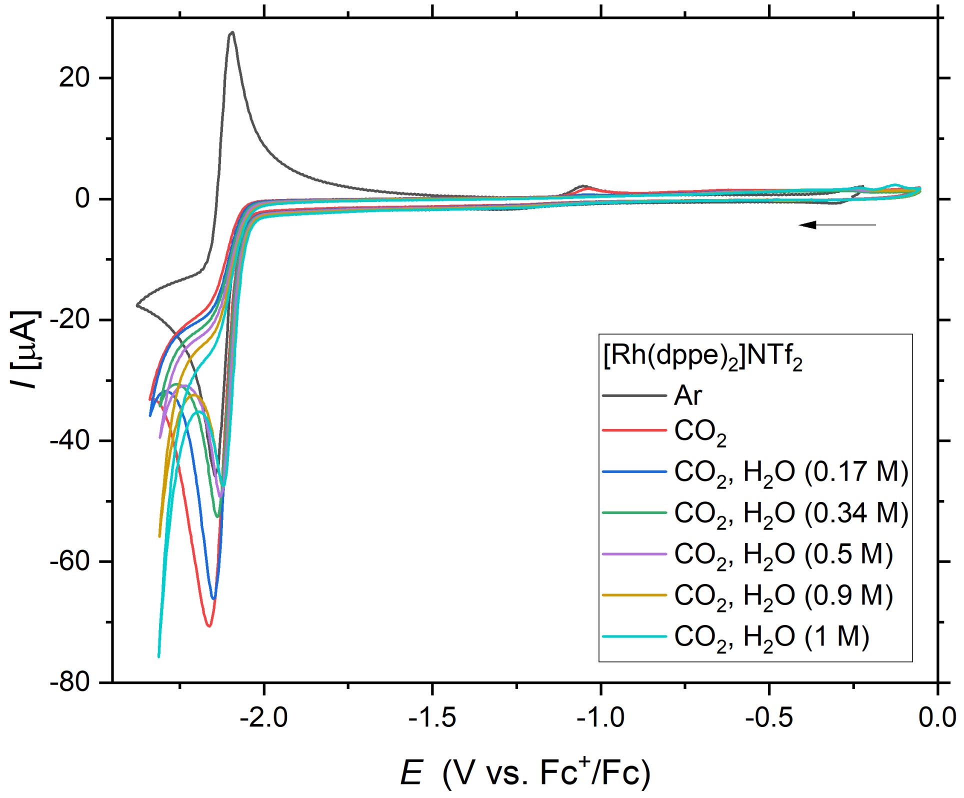

Figure S 33. Cyclic voltammograms of [Rh(dppe)$_2$]NTf$_2$ at varied H_2O concentrations. Conditions: CO_2, H_2O.

10.3 [Rh(dppe)$_2$]NTf$_2$ under Inert Conditions with Varying Electrolytes

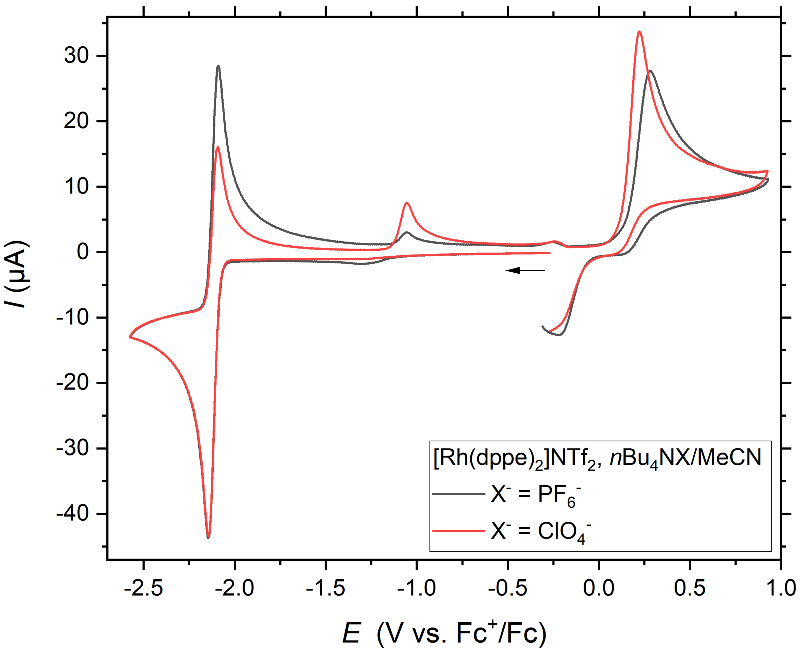

Figure S 34. Cyclic voltammograms of [Rh(dppe)$_2$]NTf$_2$ in nBu$_4$NX (0.2 M) MeCN electrolyte with X^- = PF_6^- (black) or ClO_4^- (red). Conditions: Ar.

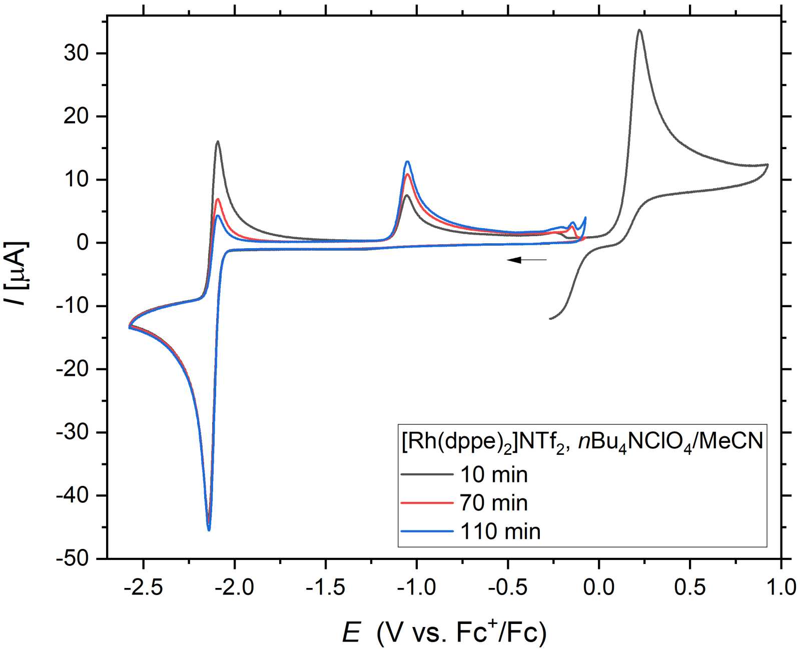

Figure S 35. Cyclic voltammograms of [Rh(dppe)$_2$]NTf$_2$ in nBu$_4$NClO$_4$ (0.2 M) MeCN electrolyte recorded over time. Conditions: Ar.

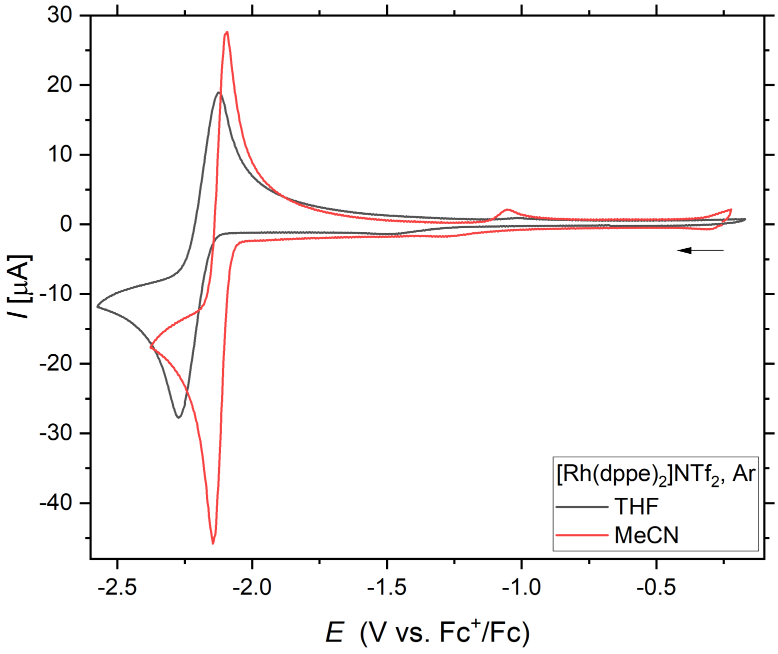

Figure S 36. Cyclic voltammograms of $[Rh(dppe)_2]NTf_2$ in 0.2 M nBu$_4$NPF$_6$ THF (black) and MeCN (red). Conditions: Ar.

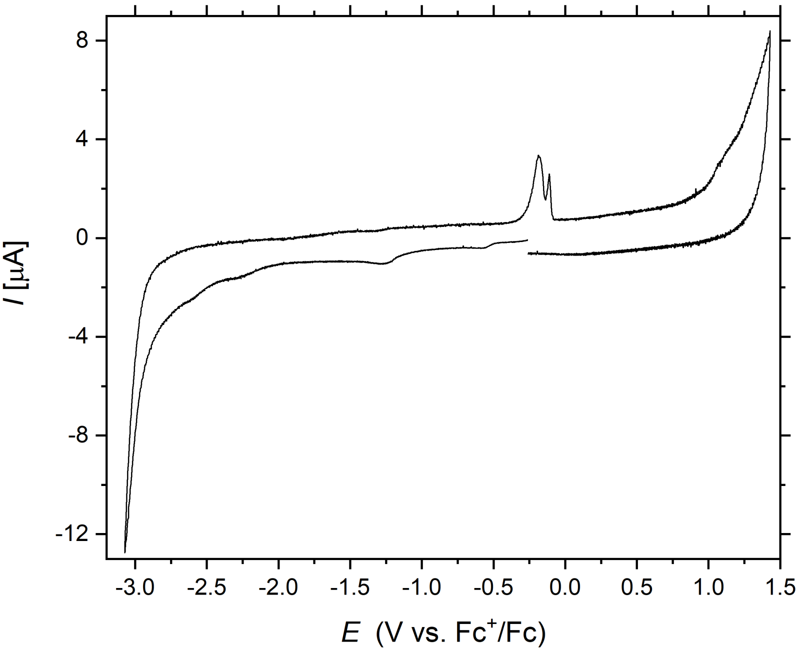

Figure S 37. Background cyclic voltammogram of 0.2 M nBu$_4$NPF$_6$/MeCN under Ar.

11. Supporting Figures: Bulk Electrolysis

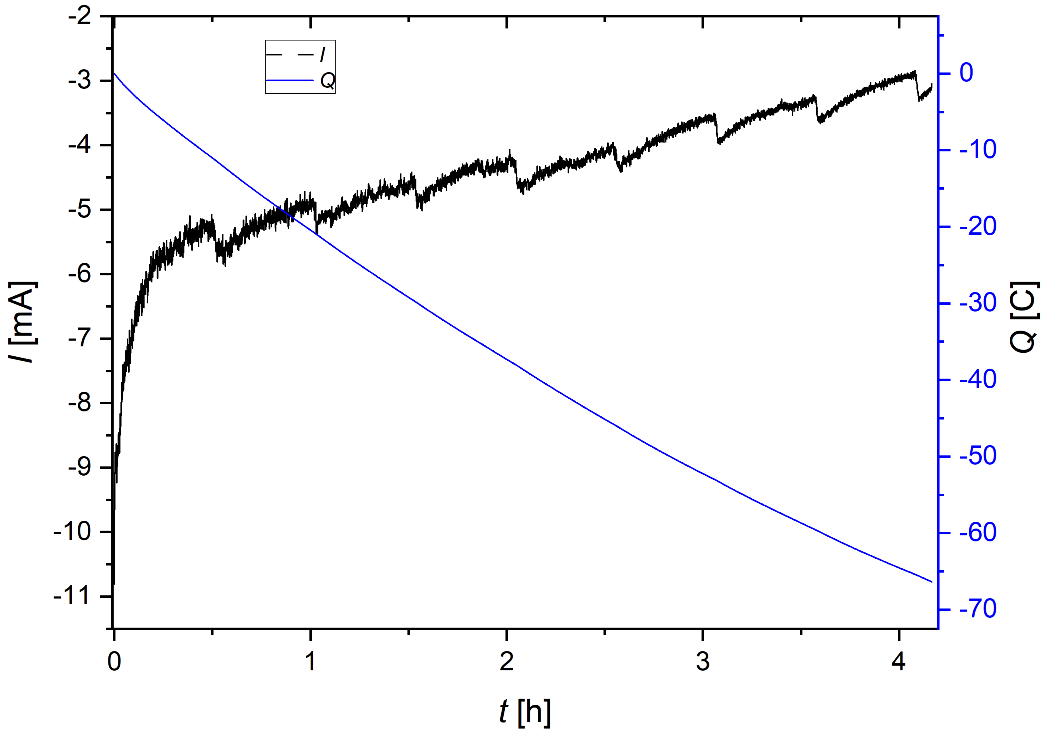

Figure S 38. Current (*I*) and charge (*Q*) time profiles of a bulk electrolysis of [Rh(dppe)$_2$]NTf$_2$ in the presence of CO_2.

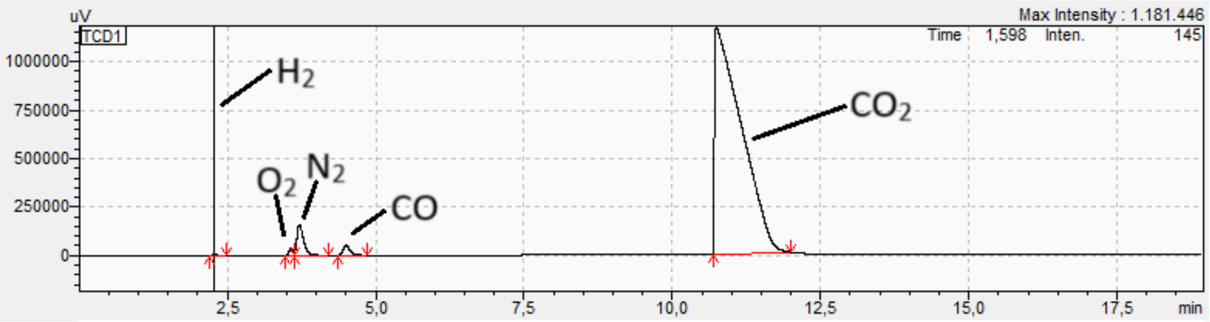

Figure S 39. Gas chromatogram collected during a bulk electrolysis of [Rh(dppe)$_2$]NTf$_2$ in the presence of CO_2 showing signals for H_2, O_2, N_2, CO and CO_2.

12. Supporting Figures: UV/Vis spectrum of $[RhH(dppe)_2]$

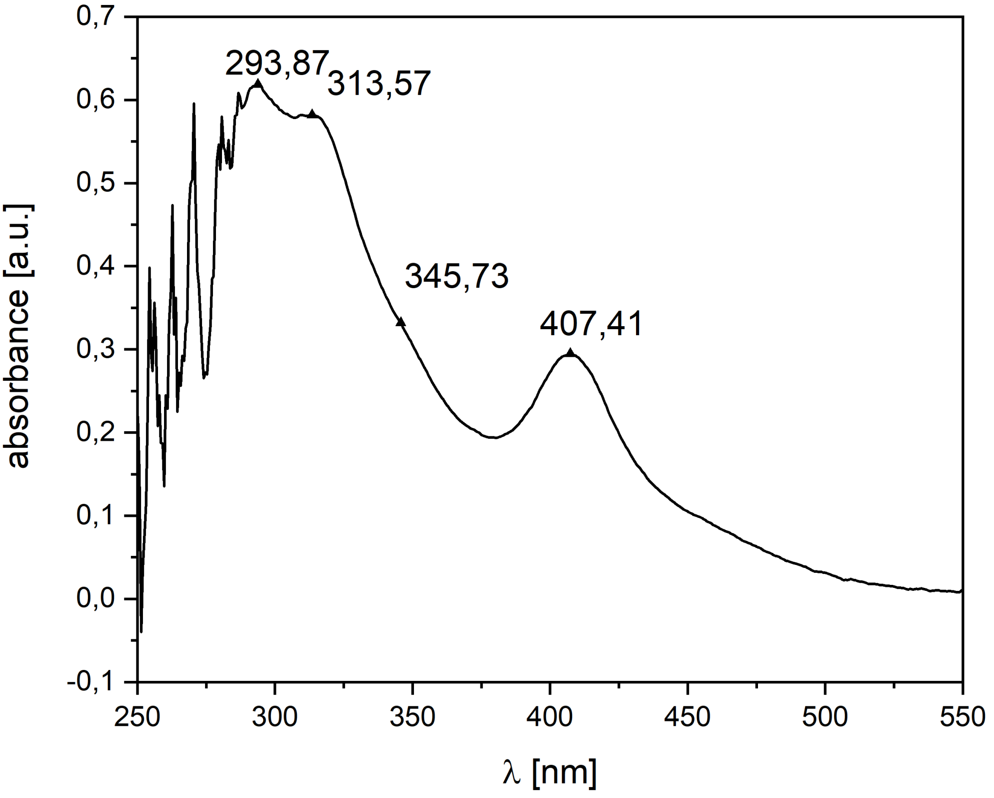

Figure S 40. UV/Vis spectrum of $[RhH(dppe)_2]$ in THF.

13. Supporting Figures: Chemical Reduction Experiments

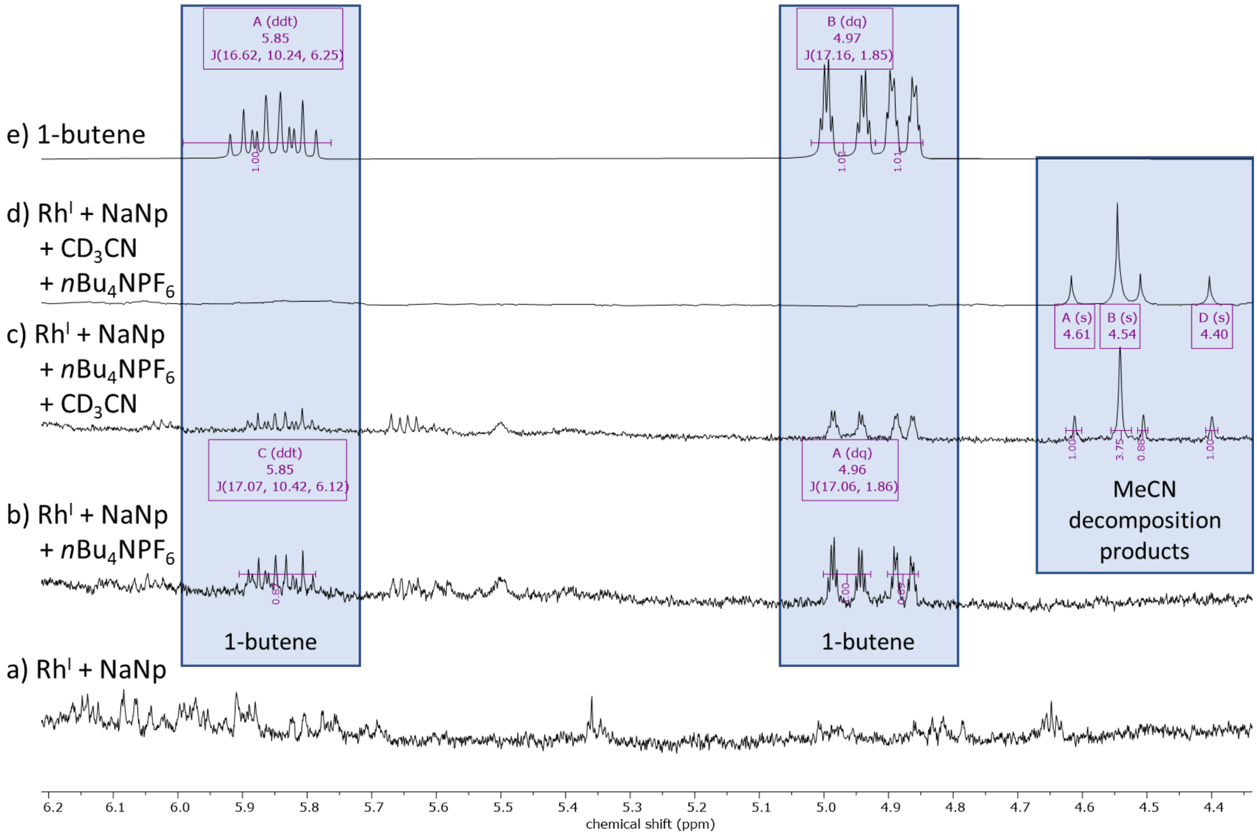

Figure S 41. Olefinic chemical shift range in ^{1}H NMR spectra (400 MHz, THF-d_8) of a) the reduction of **Rh**^{I} with excess sodium naphthalene, b) addition of $n$$Bu_4NPF_6$ to solution a, c) addition of CD_3CN to solution b, d) dissolution of dried residue from solution a in CD_3CN with added $n$$Bu_4NPF_6$, e) dissolved 1-butene. Notably, ^{31}P NMR spectra of solutions b-d show that the protonation of **Rh**$^{-I}$ is not at full conversion (see also Figures S42-S43).

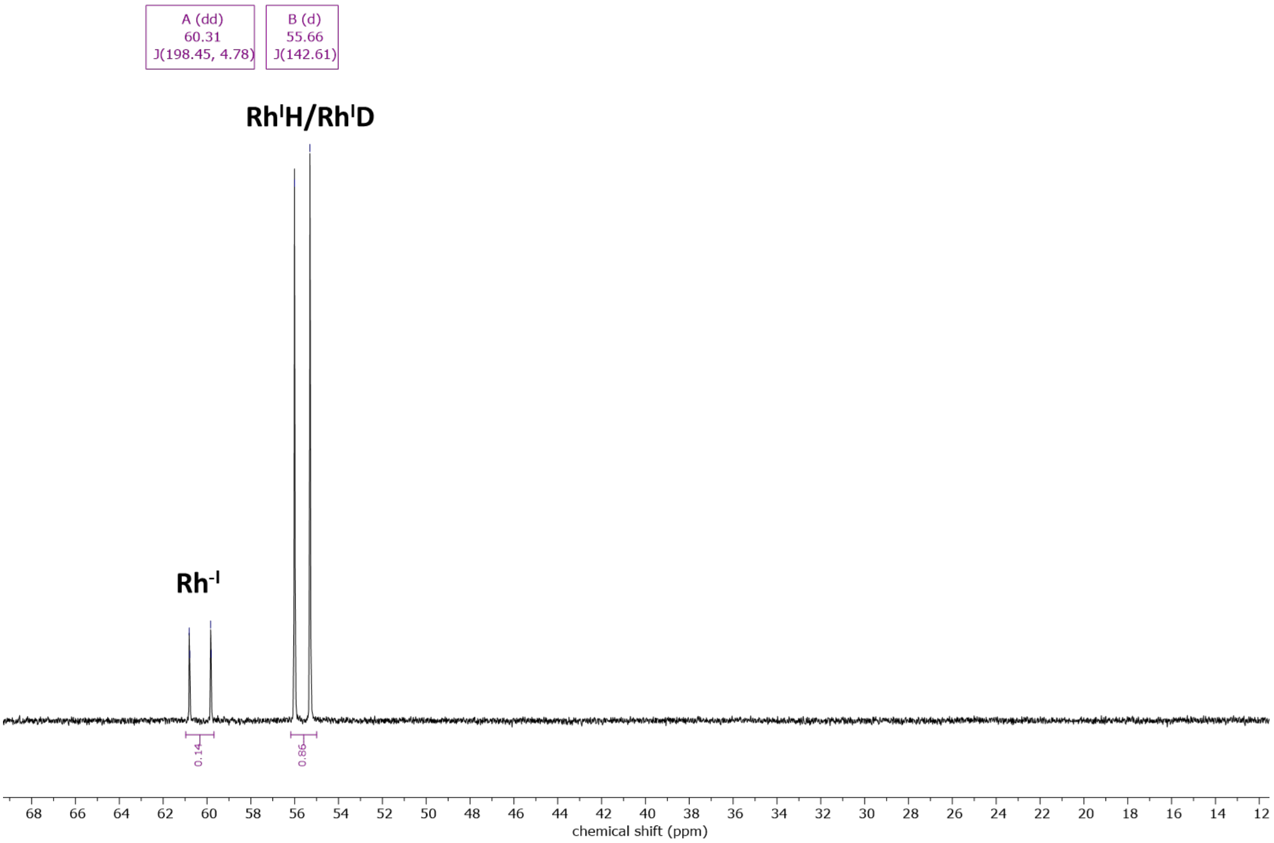

Figure S 42. ^{31}P NMR spectrum (202.4 MHz, THF-d_8) of the reaction solution c from Figure S 41 after 24 hours showing ca. 14% of residual **Rh$^{–I}$** and 86% of **Rh^{I}H**/**Rh^{I}D** resulting from protonation of **Rh$^{–I}$**.

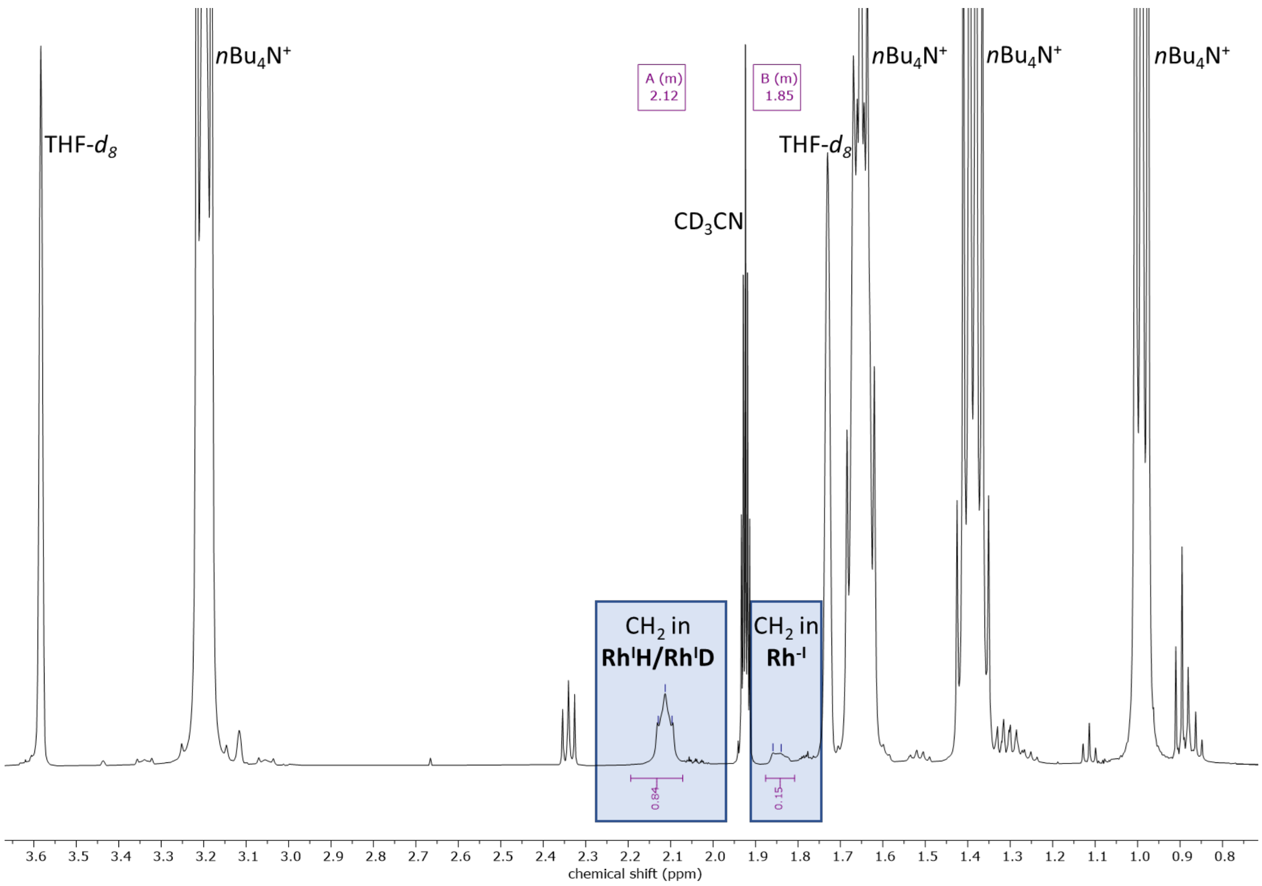

Figure S 43. Aliphatic region in the ^{1}H NMR spectrum (500 MHz, THF-d_8) of the reaction solution c from Figure S 41 after 24 hours showing ca. 15% of residual **Rh$^{–I}$** and 84% of **Rh^{I}H**/**Rh^{I}D** as indicated by integration of the CH$_2$-signals of the ligand backbone.

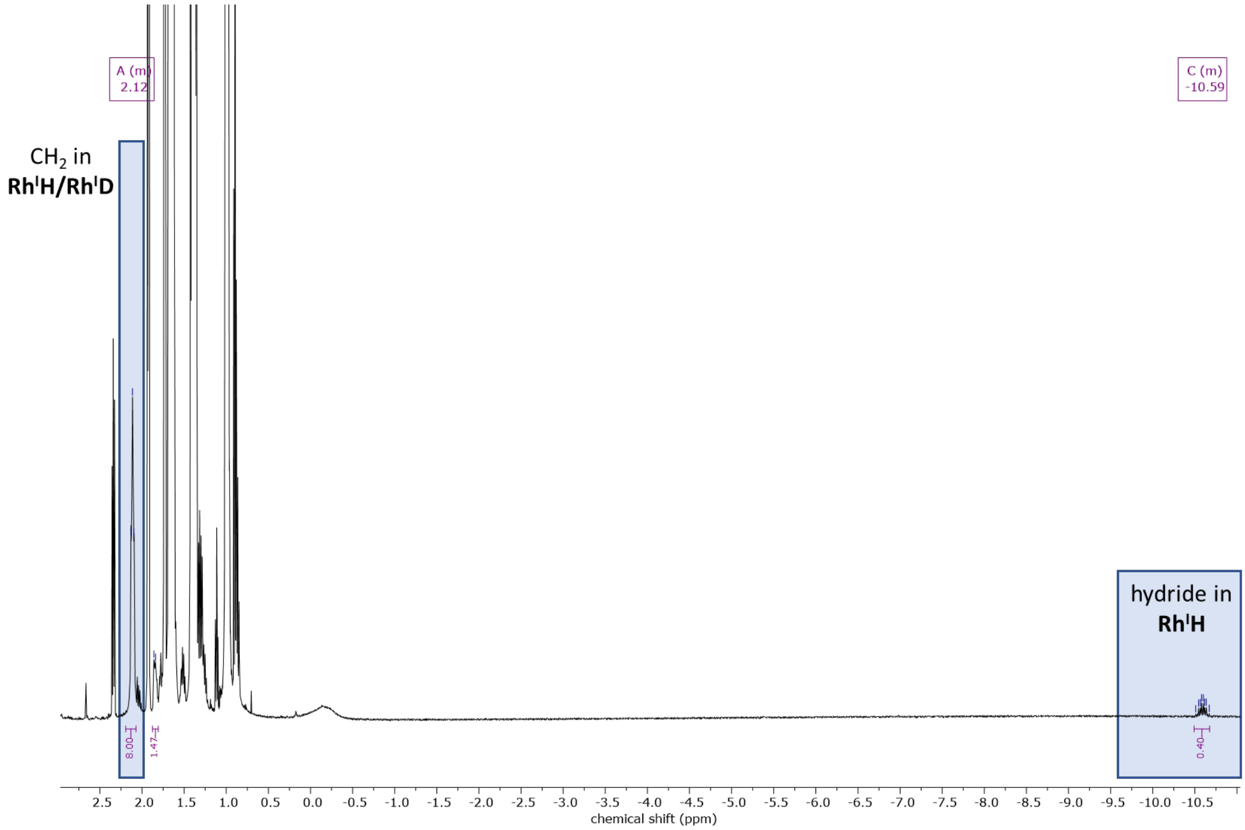

Figure S 44. Aliphatic and hydride region in the ^{1}H NMR spectrum (500 MHz, THF-d_8) of the reaction solution c from Figure S 41 after 24 hours (see also Figure S 43). Integration of the ^{1}H-hydride signal (1 H) and comparison with the ligand backbone CH_2-signals (8 H) reveals that ca 40 % of the **Rh^{I}H**/**Rh^{I}D** content is from protonation (H^+) of **Rh$^{–I}$** forming the hydride complex **Rh^{I}H**. Presumably, deuteration (D^+) of **Rh$^{–I}$** forming the deuteride complex **Rh^{I}D** is expected to account for another ca 60%, but could not be detected in ^{2}H NMR spectra.

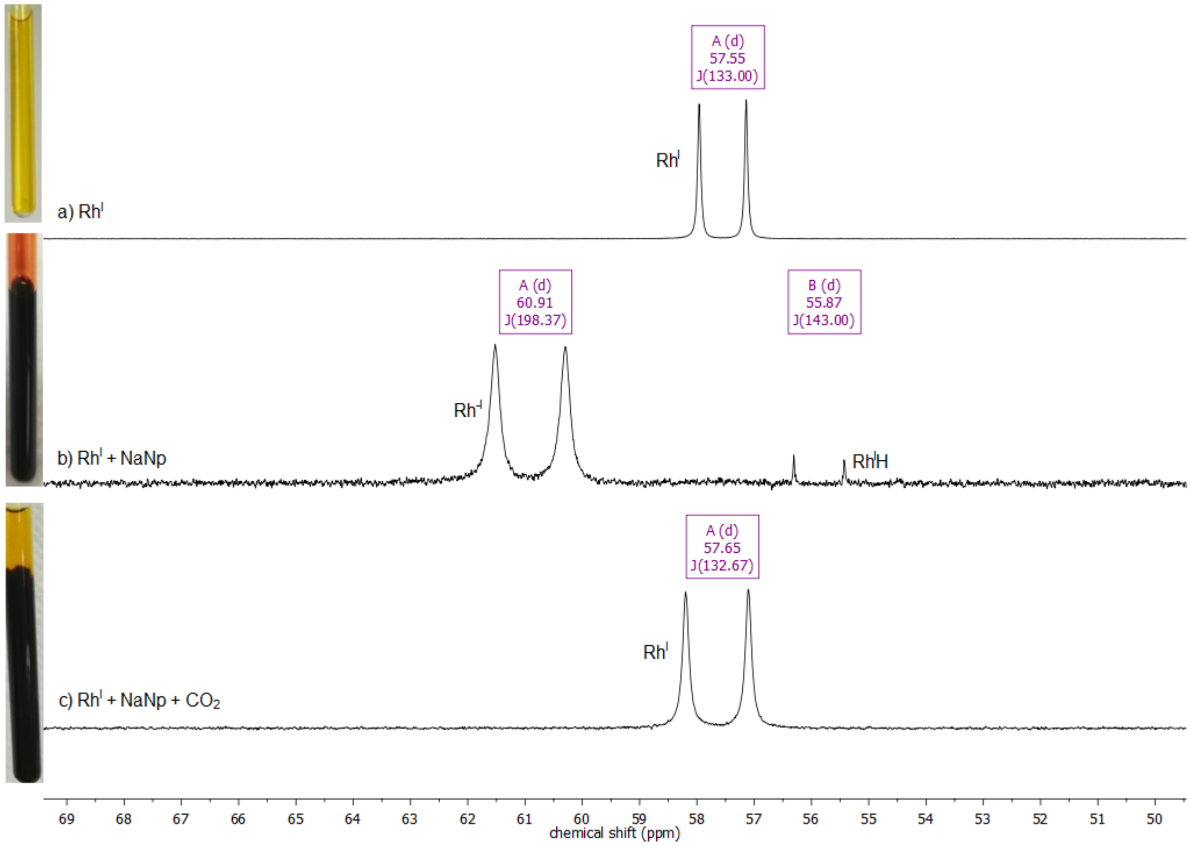

Figure S 45. ^{31}P NMR spectra (162 MHz, THF-d_8) of the reaction mixture of a) the **Rh^{I}** starting complex, b) after reduction with sodium naphtalene and c) further contacting with CO_2. NMR spectra were recorded in THF-d_8 at 162 MHz. Corresponding photos of the reaction mixture are shown to the left of the respective spectra: a) yellow solution of **Rh^{I}**, b) dark red solution of **Rh^{-I}**, c) red solution of **Rh^{-I}** turning yellow upon addition of CO_2.

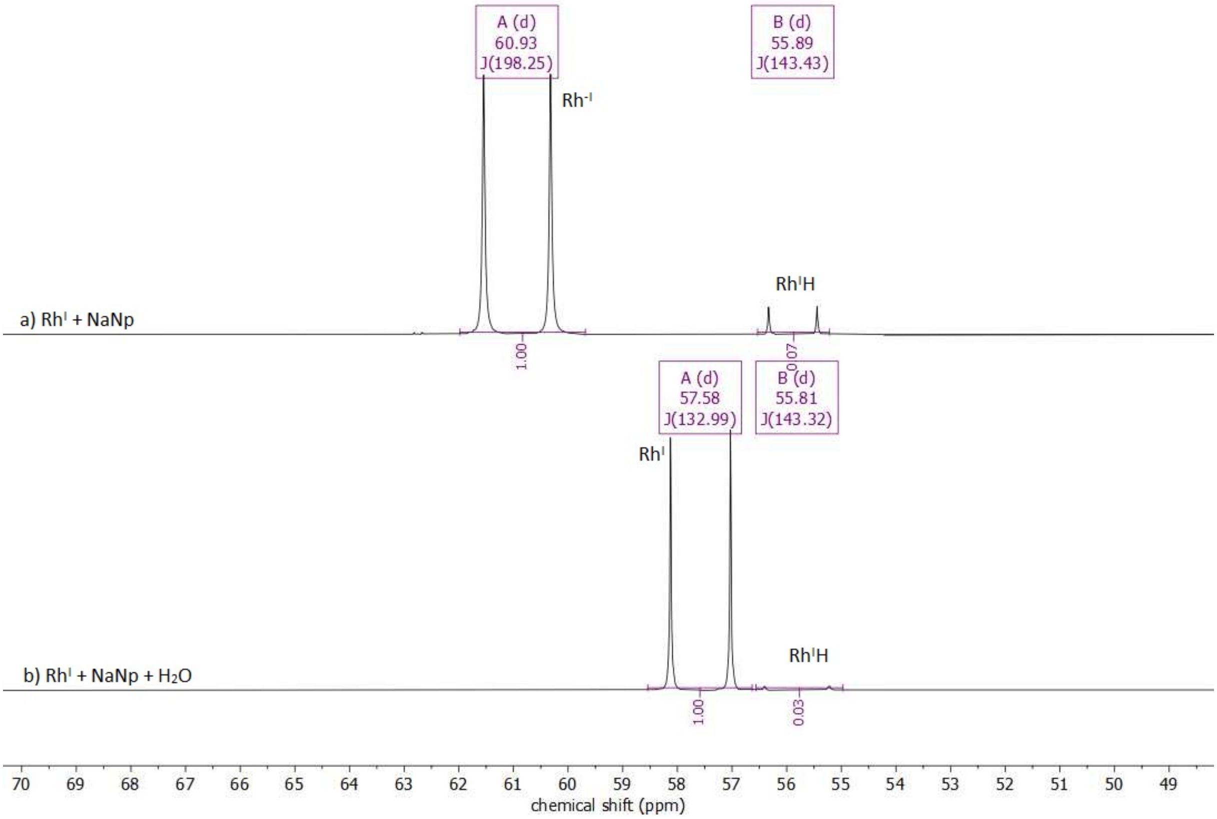

Figure S 46. ^{31}P NMR spectra (162 MHz, THF-d_8) of a) the reduction of **Rh^I** with sodium naphtalene and b) further addition of H_2O.

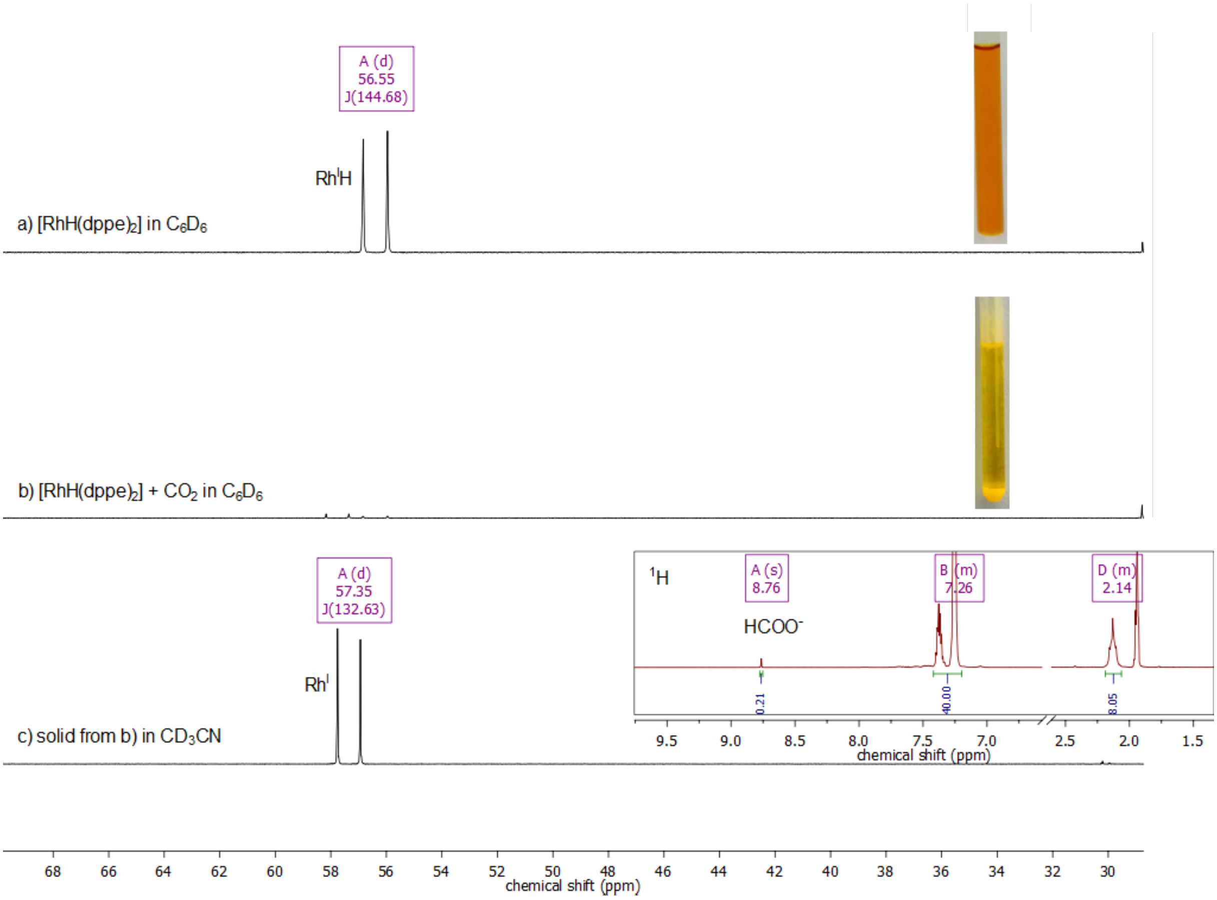

Figure S 47. ^{31}P NMR spectra (162 MHz, C_6D_6) and corresponding photos of the reaction mixtures of a) **Rh^{I}H** and b) following addition of CO_2. c) ^{31}P and excerpt from ^1H NMR spectra (400 MHz) of the solid formed in b) dissolved in CD_3CN.

14. Supporting Figures: Density Functional Theory Computations

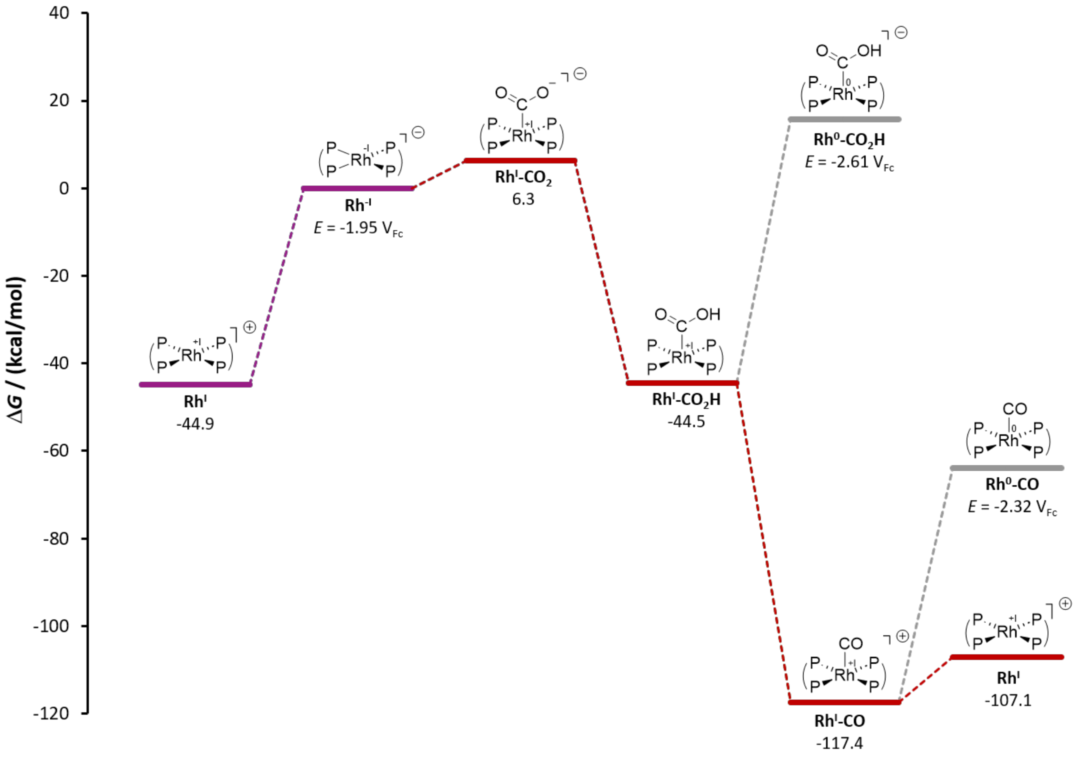

Figure S 48. Energy profile in the electrocatalytic reduction of CO_2 following the ET_M (red) pathway. Only energies of intermediates are depicted. High-energy reduction steps are shown in grey.

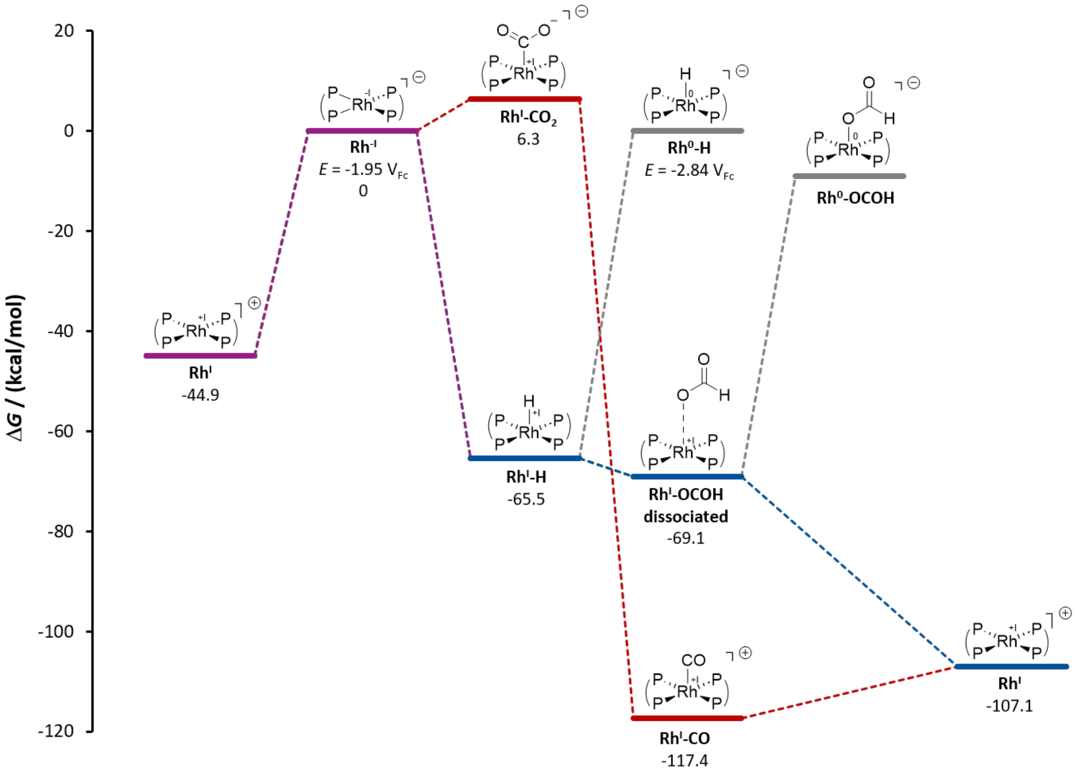

Figure S 49. Energy profile in the electrocatalytic reduction of CO_2 following the ET_H (red) pathway. Only energies of intermediates are depicted. High-energy reduction steps are shown in grey.

15. Supporting Figures: DigiElch Simulation Data

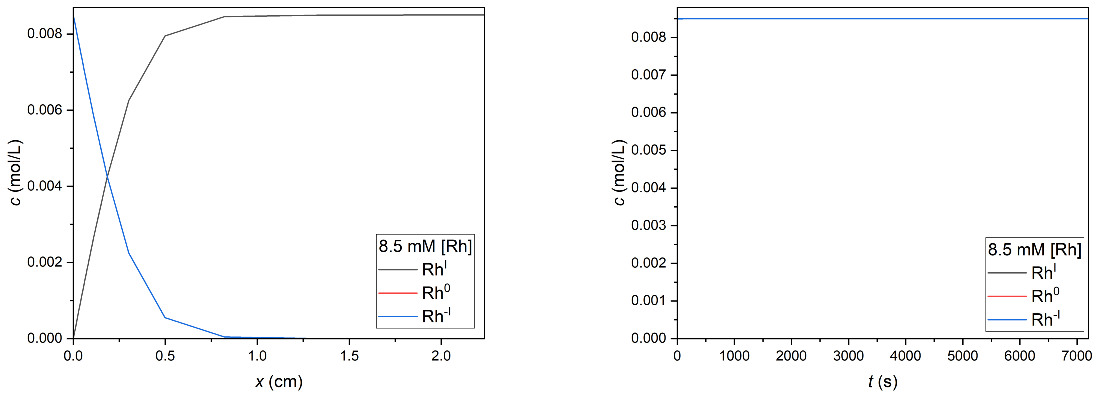

Figure S 50. Simulated concentration profiles of **Rh$^{I/0/-I}$** during controlled potential electrolysis of **Rh^{I}** under inert conditions after 120 min. Left: concentration profiles extending from the electrode surface (x = 0) into the bulk. Right: Time-dependence of surface concentrations. The bulk and surface concentration of **Rh^{0}** as well as the surface concentration of **Rh^{I}** never exceed 1.3 10^{-4} M and are invisible on the scale of the y-axis.

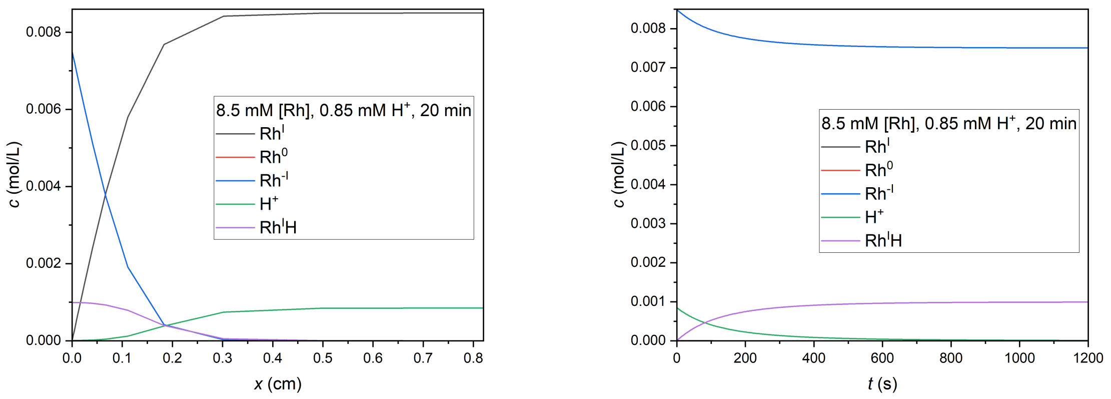

Figure S 51. Simulated concentration profiles of **Rh$^{I/0/-I}$** during controlled potential electrolysis of **Rh^{I}** under Ar in the presence of low proton concentrations (0.85 mM) after 20 min. Left: concentration profiles extending from the electrode surface (x = 0) into the bulk. Right: Time-dependence of surface concentrations. The bulk and surface concentration of **Rh^{0}** as well as the surface concentration of **Rh^{I}** never exceed 1.3 10^{-4} M and are invisible on the scale of the y-axis.

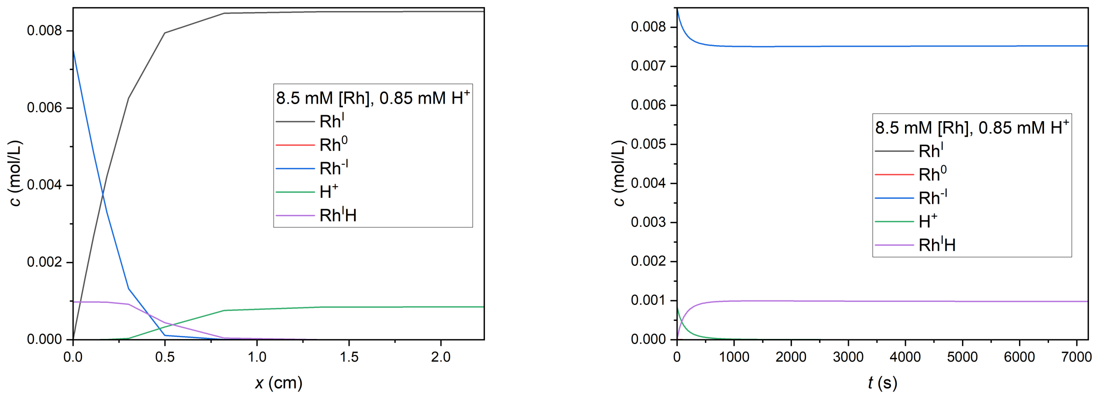

Figure S 52. Simulated concentration profiles of **Rh$^{I/0/-I}$** during controlled potential electrolysis of **Rh^{I}** under Ar in the presence of low proton concentrations (0.85 mM) after 120 min. Left: concentration profiles extending from the electrode surface (*x* = 0) into the bulk. Right: Time-dependence of surface concentrations. The bulk and surface concentration of **Rh^{0}** as well as the surface concentration of **Rh^{I}** never exceed 1.3 10^{-4} M and are invisible on the scale of the y-axis.

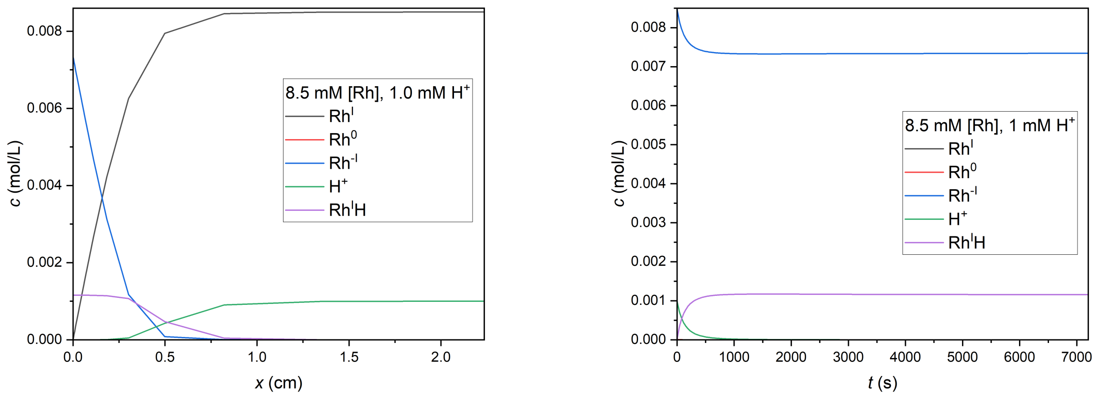

Figure S 53. Simulated concentration profiles of **Rh$^{I/0/-I}$** during controlled potential electrolysis of **Rh^{I}** under Ar in the presence of low proton concentrations (1 mM) after 120 min. Left: concentration profiles extending from the electrode surface (*x* = 0) into the bulk. Right: Time-dependence of surface concentrations. The bulk and surface concentration of **Rh^{0}** as well as the surface concentration of **Rh^{I}** never exceed 1.3 10^{-4} M and are invisible on the scale of the y-axis.

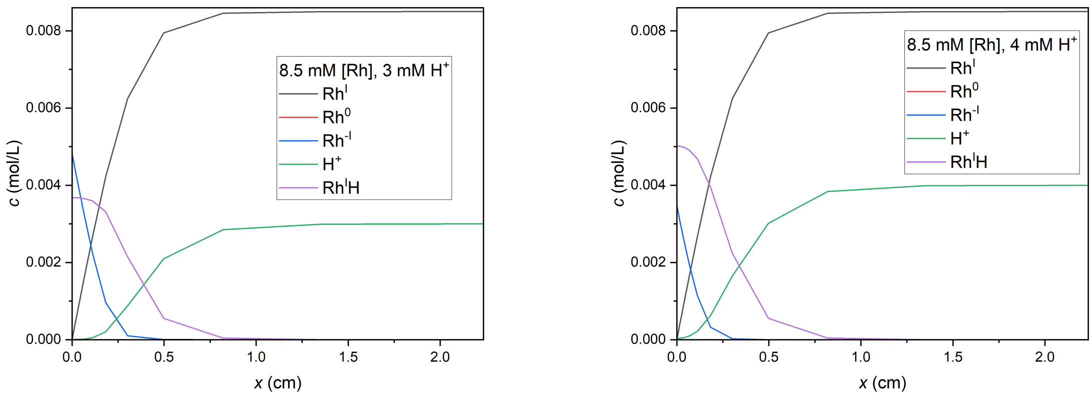

Figure S 54. Simulated concentration profiles of $\mathbf{Rh^{I/0/-I}}$ extending from the electrode surface (x = 0) into the bulk during controlled potential electrolysis of $\mathbf{Rh^{I}}$ under Ar in the presence of low proton concentrations after 120 min. Left: $[H^+]$ = 3 mM. Right: $[H^+]$ = 4 mM. The bulk and surface concentration of $\mathbf{Rh^{0}}$ as well as the surface concentration of $\mathbf{Rh^{I}}$ never exceed 1.3 10^{-4} M and are invisible on the scale of the y-axis.

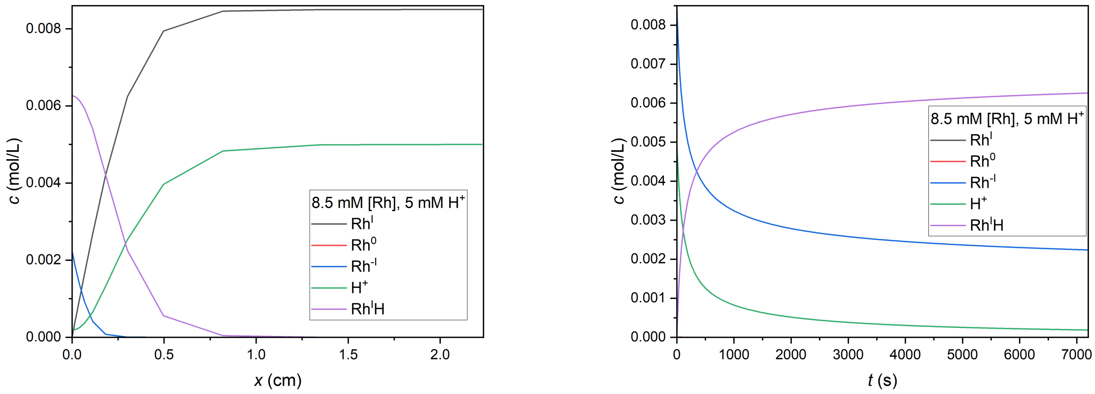

Figure S 55. Simulated concentration profiles of $\mathbf{Rh^{I/0/-I}}$ during controlled potential electrolysis of [$\mathbf{Rh^{I}}$] under Ar in the presence of low proton concentrations (5 mM) after 120 min. Left: concentration profiles extending from the electrode surface (x = 0) into the bulk. Right: Time-dependence of surface concentrations. The bulk and surface concentration of $\mathbf{Rh^{0}}$ as well as the surface concentration of $\mathbf{Rh^{I}}$ never exceed 1.3 10^{-4} M and are invisible on the scale of the y-axis.

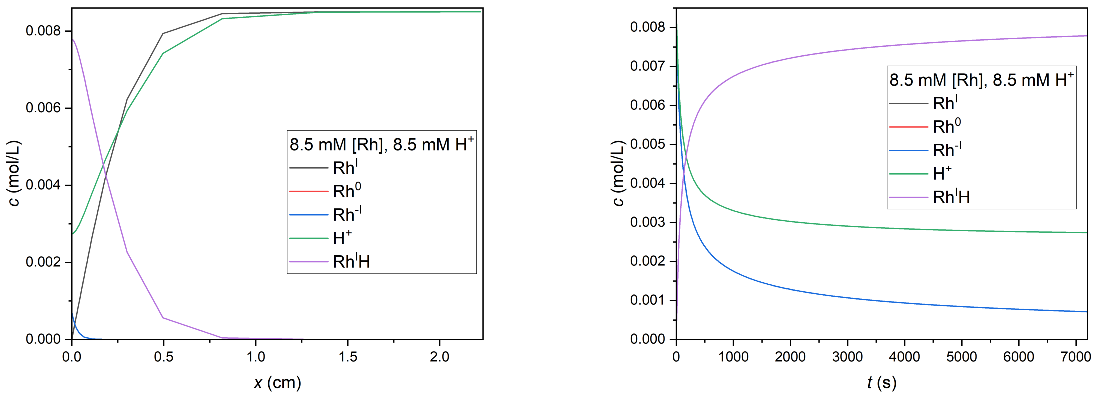

Figure S 56. Simulated concentration profiles of $\mathbf{Rh^{I/0/-I}}$ during controlled potential electrolysis of [$\mathbf{Rh^I}$] under Ar in the presence of low proton concentrations (8.5 mM) after 120 min. Left: concentration profiles extending from the electrode surface (x = 0) into the bulk. Right: Time-dependence of surface concentrations. The bulk and surface concentration of $\mathbf{Rh^0}$ as well as the surface concentration of $\mathbf{Rh^I}$ never exceed 1.3 10^{-4} M and are invisible on the scale of the y-axis.

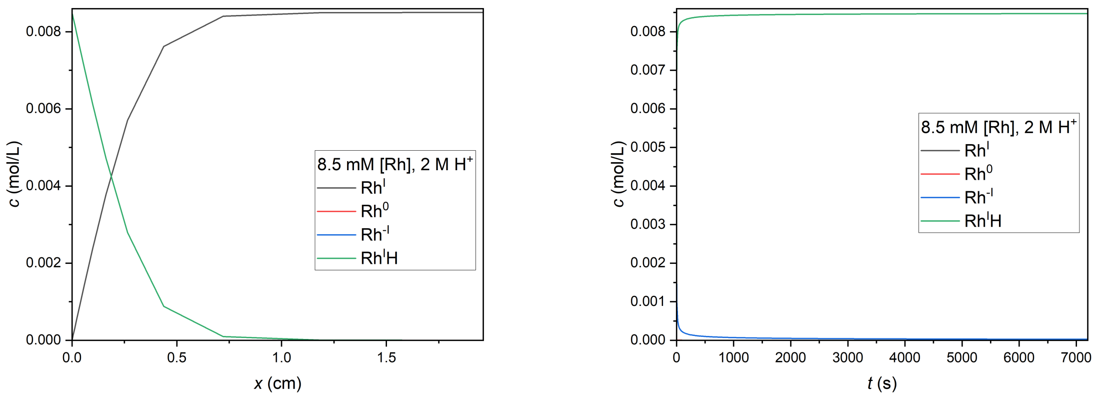

Figure S 57. Simulated concentration profiles of $\mathbf{Rh^{I/0/-I}}$ during controlled potential electrolysis of [$\mathbf{Rh^I}$] under Ar in the presence of high proton concentrations (2 M) after 120 min. Left: concentration profiles extending from the electrode surface (x = 0) into the bulk. Right: Time-dependence of surface concentrations. The bulk and surface concentration of $\mathbf{Rh^0}$ as well as the surface concentration of $\mathbf{Rh^I}$ never exceed 1.3 10^{-4} M and are invisible on the scale of the y-axis.

Table S 2. Ratio of final $\mathbf{Rh^IH}$ to $\mathbf{Rh^{-I}}$ concentrations (c_f) determined from relative integrals (in %) of experimental SEC-^{31}P NMR peaks after two hours (entry 1) or from absolute integrals (in 10^{-4} mol·cm·L^{-1}) of simulated concentration profiles (entries 2-5) at varied proton concentration (1 to 5 mM) after 120 minutes.

Entry		Conditions	c_f($\mathbf{Rh^IH}$)	c_f($\mathbf{Rh^{-I}}$)	c_f($\mathbf{Rh^IH}$): c_f($\mathbf{Rh^{-I}}$)
1	Experimental	^{31}P SEC-NMR after 2 h	34.2	6.1	5.6
2	Simulated, [H^+] (mM):	1	5.89	13.4	0.4
3		3	13.5	5.78	2.3
4		4	16.0	3.25	4.9
5		5	17.7	1.55	11.4

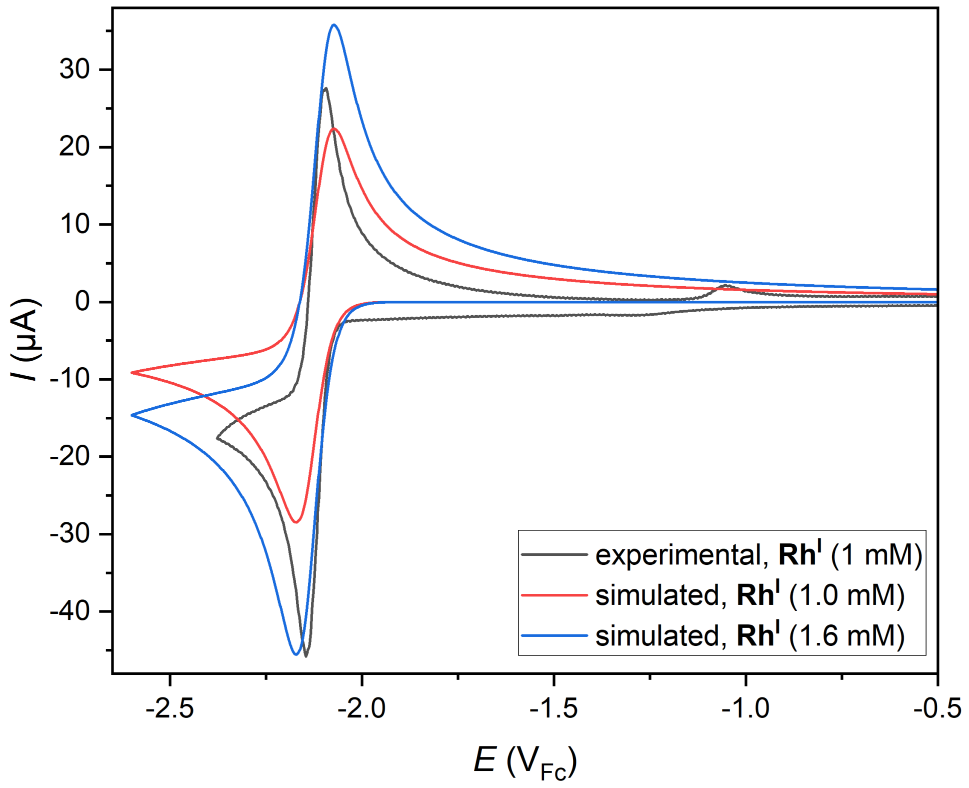

Figure S 58. Experimental (black) and simulated (coloured) cyclic voltammograms of **Rh**^{I} under inert conditions (black) and in the presence of protons (1 M) (coloured). The deviation in peak currents of experimental and simulated data at [**Rh**^{I}] = 1.0 mM and approximation of simulated to experimental values at [**Rh**^{I}] = 1.6 mM is within experimental boundaries, respectively scale (calibration) accuracy.

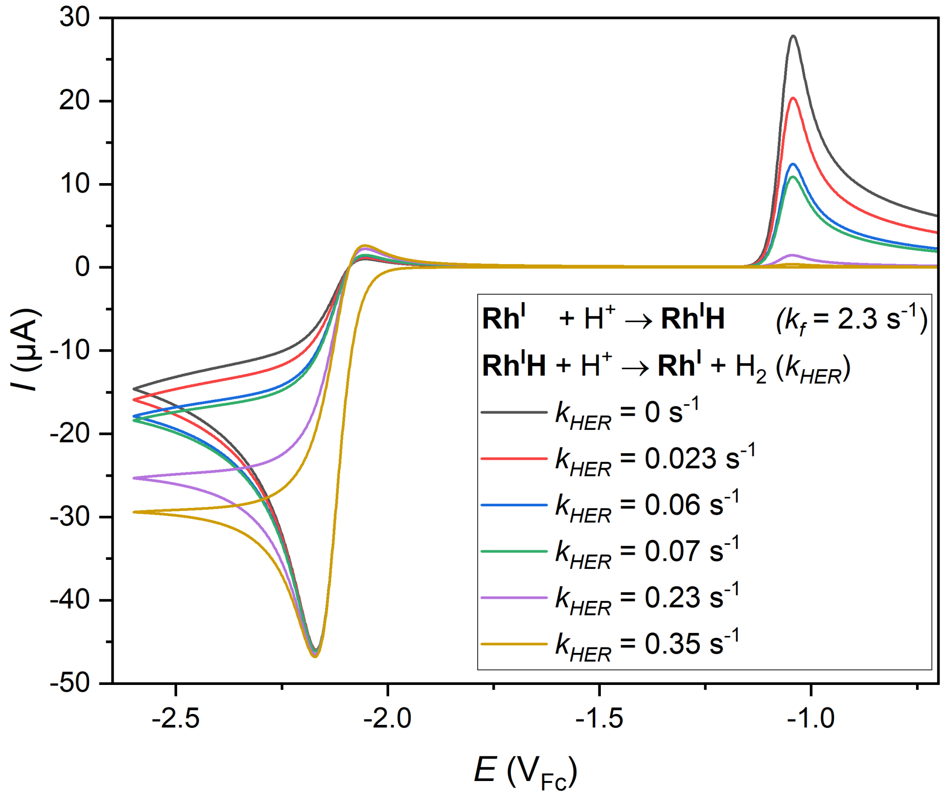

Figure S 59. Simulated cyclic voltammograms of **Rh^{I}** (1.6 mM) in the presence of protons (1 M). Considered reactions are formation of **Rh^{I}H** at the experimental rate of k_f = 2.3 s^{-1} without (black) and with subsequent hydrogen evolution at varied k_{HER} (coloured).

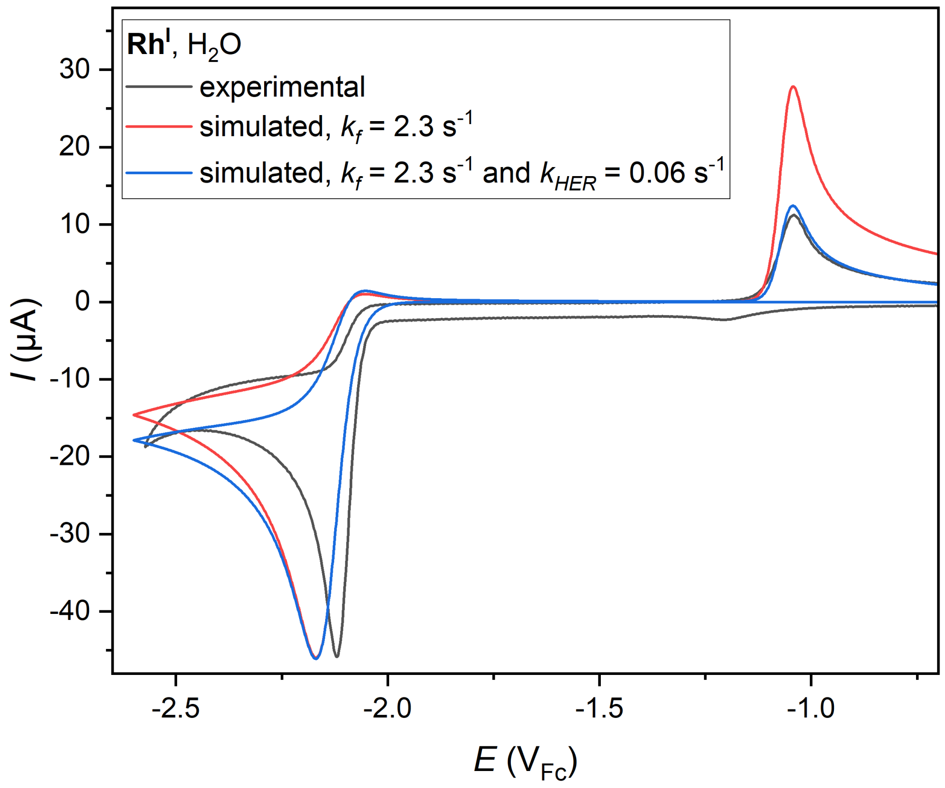

Figure S 60. Experimental (black) and simulated cyclic voltammograms of **Rh^I** (1 mM and 1.6 mM respectively) in the presence of protons (1 M). Considered reactions are formation of **Rh^IH** at the experimental rate of k_f = 2.3 s^{-1} without (red) and with subsequent hydrogen evolution at k_{HER} = 0.06 s^{-1} (blue). For better comparability between experimental and simulated CVs (see Figure S58), [**Rh^I**] was increased to 1.6 mM in simulations. This deviation is within experimental boundaries.

16. References

1. A. C. Kick, T. Weyhermuller, M. Holscher, N. Kaeffer and W. Leitner, *Angew. Chem. Int. Ed.*, 2024, **63**, e202408356.

2. N. Ahmad, J. J. Levison, S. D. Robinson, M. F. Uttley, E. R. Wonchoba and G. W. Parshall, in *Inorganic Syntheses*, **1990**, pp. 81–83.

3. M. Arisawa, T. Ichikawa and M. Yamaguchi, *Chem. Commun.*, 2015, **51**, 8821–8824.

4. A. J. Kunin, E. J. Nanni and R. Eisenberg, *Inorg. Chem.*, 1985, **24**, 1852–1856.

5. J. A. Sofranko, R. Eisenberg and J. A. Kampmeier, *J. Am. Chem. Soc.*, 1979, **101**, 1042–1044.

6. S. Jovanovic, P. P. M. Schleker, M. Streun, S. Merz, P. Jakes, M. Schatz, R. A. Eichel and J. Granwehr, *Magn. Reson.*, 2021, **2**, 265–280.

7. P. F. d. Silva, B. F. Gomes, C. M. S. Lobo, L. H. K. Queiroz Júnior, E. Danieli, M. Carmo, B. Blümich and L. A. Colnago, *Microchem. J.*, 2019, **146**, 658-663.

8. G. Pilloni, G. Zotti and M. Martelli, *Inorg. Chem.*, 1982, **21**, 1283–1284.

9. H. Schmidt and J. Noack, *Z. Anorg. Allg. Chem.*, 1958, **296**, 262–272.

10. P. Krtil, L. Kavan and P. Novák, *J. Electrochem. Soc.*, 1993, **140**, 3390–3395.

11. J. A. Sofranko, R. Eisenberg and J. A. Kampmeier, *J. Am. Chem. Soc.*, 1980, **102**, 1163–1165.

12. G. W. T. M. J. Frisch, H. B. Schlegel, G. E. Scuseria, J. R. C. M. A. Robb, G. Scalmani, V. Barone, H. N. G. A. Petersson, X. Li, M. Caricato, A. V. Marenich, B. G. J. J. Bloino, R. Gomperts, B. Mennucci, H. P. Hratchian, A. F. I. J. V. Ortiz, J. L. Sonnenberg, D. Williams-Young, F. L. F. Ding, F. Egidi, J. Goings, B. Peng, A. Petrone, D. R. T. Henderson, V. G. Zakrzewski, J. Gao, N. Rega, W. L. G. Zheng, M. Hada, M. Ehara, K. Toyota, R. Fukuda, M. I. J. Hasegawa, T. Nakajima, Y. Honda, O. Kitao, H. Nakai, K. T. T. Vreven, J. A. Montgomery, Jr., J. E. Peralta, M. J. B. F. Ogliaro, J. J. Heyd, E. N. Brothers, K. N. Kudin, T. A. K. V. N. Staroverov, R. Kobayashi, J. Normand, A. P. R. K. Raghavachari, J. C. Burant, S. S. Iyengar, M. C. J. Tomasi, J. M. Millam, M. Klene, C. Adamo, R. Cammi, R. L. M. J. W. Ochterski, K. Morokuma, O. Farkas, J. B. Foresman and D. J. Fox, Gaussian, Inc., Wallingford CT, **2016**.

13. Y. Zhao and D. G. Truhlar, *J. Chem. Phys.*, 2006, **125**, 194101.

14. F. Weigend and R. Ahlrichs, *Phys. Chem. Chem. Phys.*, 2005, **7**, 3297-3305.

15. A. Schäfer, H. Horn and R. Ahlrichs, *J. Chem. Phys.*, 1992, **97**, 2571–2577.

16. A. Schäfer, C. Huber and R. Ahlrichs, *J. Chem. Phys.*, 1994, **100**, 5829–5835.

17. A. V. Marenich, C. J. Cramer and D. G. Truhlar, *J. Phys. Chem. B*, 2009, **113**, 6378–6396.

18. R. L. Martin, P. J. Hay and L. R. Pratt, *J. Phys. Chem. A*, 1998, **102**, 3565–3573.

19. F. Weigend, *Phys. Chem. Chem. Phys.*, 2006, **8**, 1057-1065.

20. K. Rohmann, M. Hölscher and W. Leitner, *J. Am. Chem. Soc.*, 2016, **138**, 433-443.

21. A.-C. Kick, T. Weyhermüller, M. Hölscher, N. Kaeffer and W. Leitner, *Angew. Chem. Int. Ed.*, 2024, **n/a**, e202408356.

22. F. P. Lees and P. Sarram, *J. Chem. Eng. Data*, 1971, **16**, 41-44.

23. K. Sporka, J. Hanika, V. Růžička and M. Halousek, *Collect. Czechoslov. Chem. Commun.*, 1971, **36**, 2130-2136.

24. K. Sporka, J. Hanika and V. Růžička, *Collect. Czechoslov. Chem. Commun.*, 1969, **34**, 3145-3148.

25. R. J. Littel, G. F. Versteeg and W. P. M. Van Swaaij, *J. Chem. Eng. Data*, 1992, **37**, 42-45.

26. H. Mayr and A. R. Ofial, *Acc. Chem. Res.*, 2016, **49**, 952-965.

27. R. H. Morris, *J. Am. Chem. Soc.*, 2014, **136**, 1948-1959.

28. K. T. Mueller, A. J. Kunin, S. Greiner, T. Henderson, R. W. Kreilick and R. Eisenberg, *J. Am. Chem. Soc.*, 1987, **109**, 6313–6318.
